# A Comprehensive Inter-Tissue Crosstalk Analysis Underlying Progression and Control of Obesity and Diabetes

**DOI:** 10.1038/srep12340

**Published:** 2015-07-23

**Authors:** Pawan Samdani, Meet Singhal, Neeraj Sinha, Parul Tripathi, Sachin Sharma, Kamiya Tikoo, Kanury V. S. Rao, Dhiraj Kumar

**Affiliations:** 1 Department of Biochemical Engineering and Biotechnology, Indian Institute of Technology, Hauz Khas, New Delhi-110016; 2 Immunology Group, International Centre for Genetic Engineering and Biotechnology, Aruna Asaf Ali Marg, New Delhi-110067.

## Abstract

Obesity is a metabolic state associated with excess of positive energy balance. While adipose tissues are considered the major contributor for complications associated with obesity, they influence a variety of tissues and inflict significant metabolic and inflammatory alterations. Unfortunately, the communication network between different cell-types responsible for such systemic alterations has been largely unexplored. Here we study the inter-tissue crosstalk during progression and cure of obesity using multi-tissue gene expression data generated through microarray analysis. We used gene expression data sets from 10 different tissues from mice fed on high-fat-high-sugar diet (HFHSD) at various stages of disease development and applied a novel analysis algorithm to deduce the tissue crosstalk. We unravel a comprehensive network of inter-tissue crosstalk that emerges during progression of obesity leading to inflammation and insulin resistance. Many of the crosstalk involved interactions between well-known modulators of obesity and associated pathology like inflammation. We then used similar datasets from mice that in addition to HFHSD were also administered with a herbal concoction known to circumvent the effects of HFHSD in the diet induced model of obesity in mice. We propose, the analysis presented here could be applied to understand systemic details of several chronic diseases.

Obesity has emerged as a global epidemic and lies at the forefront of a vast repertoire of metabolic/life style diseases in developing and developed world^1^. Obesity is manifested by an increased body mass index (BMI) presumably as a result of excessive calorie uptake accompanied with reduced physical activity^2^,^3^. Obesity is strongly implicated in the recent surge in chronic diseases like type2 diabetes, insulin resistance and cardiovascular diseases^2^. While certain genetic associations on susceptibility for developing obesity among individuals have emerged^4^, in most cases obesity and related illness remain a life-style disease associated with an enhanced positive energy balance^2^.

The positive energy balance leads to accumulation of stored fats in the adipose tissues across the body^5^. While the adipose tissues serve as a major site for storage of excessive fat, the physiological effects of obesity are much more widespread, affecting nearly all the organs of the organism. Recent studies propose obesity as a low-grade chronic inflammatory disease^6^. It is known that adipose tissues in addition to storing excessive fats can also secrete cytokines, hormones and adipokines, which act as mediators of inflammation influencing a variety of target cells, tissues or organs^7^. Hormonal, cytokine or adipokine mediated inflammation has been greatly studied in the past to gain cellular and molecular level understanding of the disease^6^. Intriguingly the physiological communication between different tissues and organs as a consequence of energy surplus is now considered crucial for the systemic entrenchment of disease manifestation^2^. Despite this understanding however, a systematic physiological framework of obesity intertwining different organs and tissues at the organismal level has largely been unexplored.

Several studies implicate changes in the food habits due to surplus availability of food and a sedentary life-style as a major factor behind the surge in obesity and obesity related illness. Consequently animal models of diet-induced obesity have been the major experimental model to achieve better understanding of the disease physiology and possible intervention strategies^8^. Here we report studies on the mouse model of diet-induced obesity, leading to the unravelling of unprecedented inter-tissue crosstalk that gets estabished on the course of disease progression and treatment. We analysed gene expression analysis by microarray of ten different tissues from diet-induced obese mice including adipose tissues, infiltrating macrophages, liver and skeletal muscle. In parallel, we also used an ethno-botanical formulation with established corrective effect in the same model^9^. The comparative analysis of tissue crosstalk between the two conditions revealed novel insights on inter-tissue communications during disease progression. The proposed approach will provide novel insights on disease physiology in the context of inter-organ interactions and targets for interventions to control this global epidemic.

## Materials and Methods

### Ethics statement

All animal studies were carried out at BIONEEDS Laboratory Animals & Preclinical Services, Bangalore, India, and approved by Institutional animal ethics committee (IAEC). All experimental protocols were performed in accordance with the approved national and international guidelines. BIONEEDS is approved by the committee for the purpose of control and supervision of experiments on animals (CPCSEA), Ministry of Forests and Environments, Government of India.

### Animals, diets, treatments

Animals and diet, treatment with Kal-1, tissue isolation, RNA isolation and microarray experiments has been described our earlier report and are briefly explained in the suplementary informations^9^,^10^.

### Data Analysis

The list of extracellular (GO:0005615) and cell surface (GO:0009986) mouse gene products was downloaded from the AmiGO gene ontology browser (http://amigo1.geneontology.org/cgi-bin/amigo/go.cgi). The protein-protein interactions between the extracellular (secreted) and cell surface (receptor) gene products were extracted from major expert-curated databases (APID and InnateDB) in an automated manner using the web service PSICQUIC (https://code.google.com/p/psicquic/)^11^. A Perl script was written to access and query the databases. The curation of interaction data was done manually using Microsoft Excel. The expression data for the genes, in the curated interactions list, was extracted for all 10 tissues at all the time points using a Java program and the interactions along with the expression data of interacting genes was stored in a SQL database for downstream analysis.

### Tissue crosstalk (static analysis)

For each secreted-receptor interaction, from the curated interactions list, a tissue interaction matrix (10 tissues for secreted by 10 tissues for receptor) was constructed by assigning the values 0 or 1 to each cell. Value of 1 was assigned to a cell only if both the secreted and receptor genes, for the corresponding tissues, were up-regulated (expression value > 1). Value 0 was assigned to all other cells. SQL queries were written to obtain the tissue interaction matrix for each secreted-receptor interacting pair at each time point. For each of the 100 possible tissue interaction all the 1 s, from each tissue interaction matrix, were summed (interaction count) at each time point. Along with it, the sum of secreted genes up-regulated (sec count) and the sum of receptor genes up-regulated (rec count) was extracted for each tissue at each time point using SQL queries. Networks were constructed at each time point to visualize the tissue crosstalk, with rec count and sec count as node attributes and interaction count as edge attribute, using Cytoscape^12^.

### Tissue crosstalk (dynamic analysis)

#### Time-course trend

Time-course trend was generated for each secreted and receptor genes by assigning either of the three values: −1, 0 or 1 (trend values) at each of the time points, based on the difference between the expression value at that time point and the baseline expression (the expression value of the closest previous time point for which the trend value is not 0, if it exists or else the expression value of the first time point). If the difference is greater than or equal to 1, the value 1 is assigned. If the difference is less than or equal to −1, the value −1 is assigned. If the difference is between −1 to 1, the value 0 is assigned. Following script was used to calculate the trend:


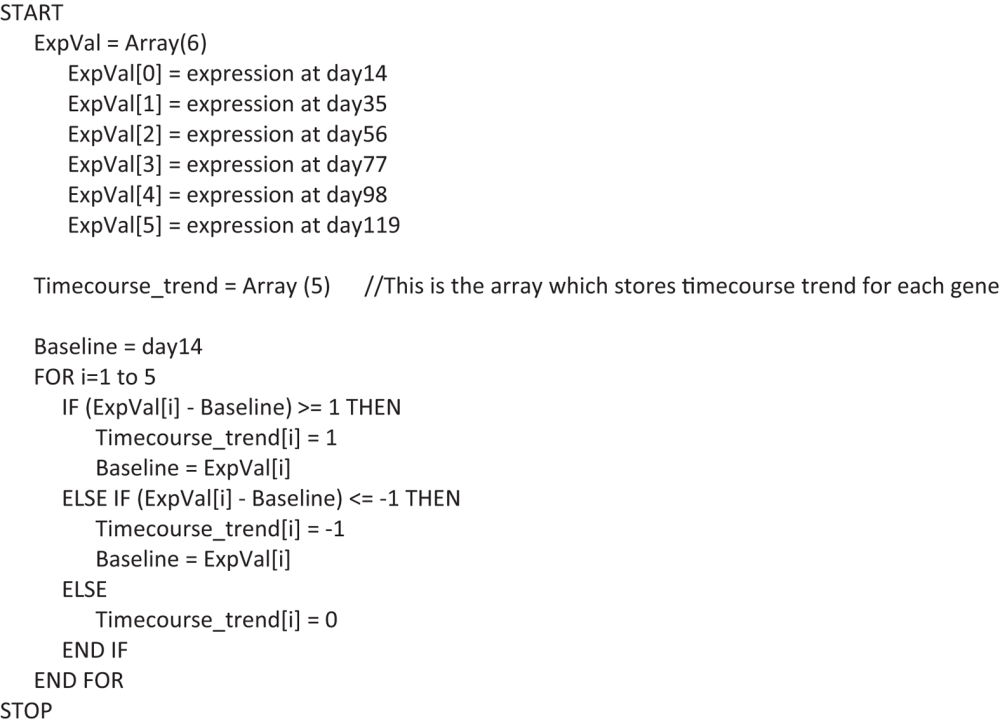


### Dynamic tissue crosstalk

The time-course trend of secreted gene and receptor gene, of a secreted-receptor interaction pair, is generated for all tissues using a Java program. A tissue interaction matrix was generated (see Tissue crosstalk for static analysis) and the value 1 was assigned only if the time-course trend of the secreted and receptor gene matched exactly for the corresponding tissues otherwise value 0 was assigned. Tissue crosstalk was visualized using network generated with Cytoscape (see Tissue crosstalk for static analysis)^12^. In a separate analysis, we also selected those interactions where time-course trend of the secreted and receptor genes was exactly opposite.

### Expression regulation in dynamic tissue crosstalk

For each gene, the expression at each of the time points was normalized with respect to the expression at the first time point and then the area under the graph (expression area), of the plotted normalized expression value, was determined using a Java program. A cut-off had to be designed in order to classify the genes as up-regulated or down-regulated based on the expression area. The cut-off for classifying up-regulation was defined as the expression area obtained from a hypothetical gene which has an expression value of 1 at exactly one time point and 0 at all the other time points. The cut-off for classifying down-regulation was defined as the expression area obtained from a hypothetical gene which has an expression value of −1 at exactly one time point and 0 at all the other time points. The dynamic interactions were classified into four groups (Sec Up - Res Up, Sec Up - Res Down, Sec Down - Res Up, Sec Down - Res Down) based on the regulation of the sec and rec genes involved in the interaction.

### Statistics

Statistical significance testing was carried out by performing hypothesis testing of the difference between two population proportions. We calculated Z-value, standard error and the corresponding P-value for each data point in Microsoft Excel.

## Results

### The schematic flow chart to study tissue crosstalk during establishment of obesity

**Figure 1:**
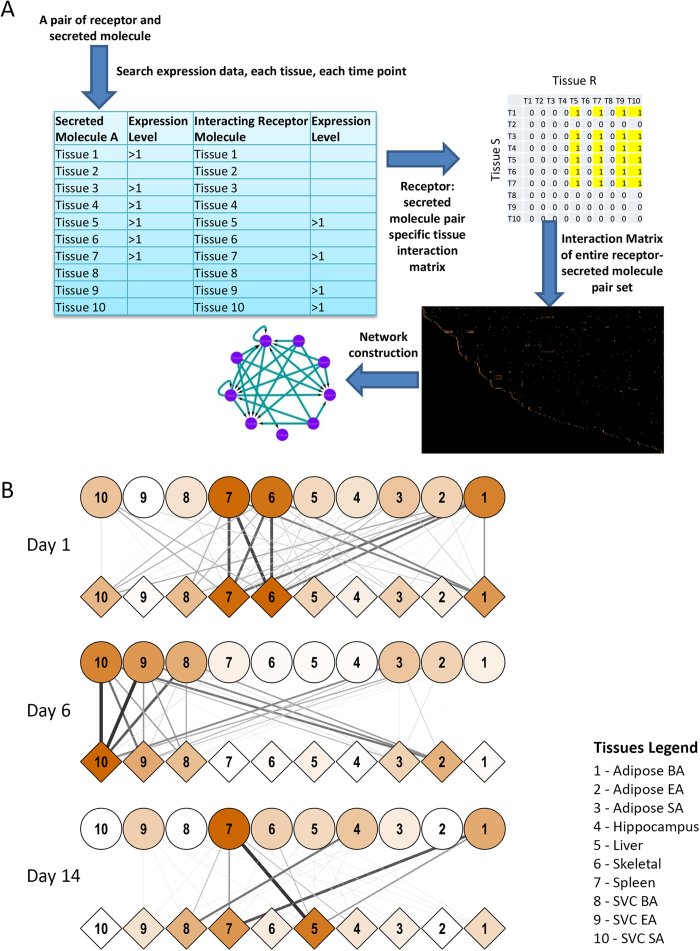
Schematic flow chart to study tissue-cross talkand Early tissue crosstalk network. The flow-chart in panel A represents the basis of identifying tissue crosstalk in this study. For a pair of receptor and secreted molecule (ligand), their expressions across all the ten tissues were compared. Any pair of tissues, where one showed increased expression of the ligand while the other for the receptor were considered to crosstalk at that particular time-point. Similar exercise was performed for all receptor-ligand pairs and all 100 possible tissue pairs (including self-interactions). For panel B, the inter-tissue crosstalk, was calculated at 1, 6 and 14 days post putting mice on HFHSD. The microarray analysis from across the 10 tissues were analysed to find possible interactions between the tissues. Circles represent tissues expressing ligands while diamonds represent tissues expressing receptors. Colour of diamond or circle describe number of interactions that they participate in (dark colour→more interactions). Thickness and colour of connections depicts frequency of receptor-ligand interactions taking place between a pair of tissues.

In order to study the crosstalk between various tissues during the establishment of obesity/diabetes in an unbiased manner, we previously reported changes in the expression level of genes across 10 different tissues across multiple time points while being fed on high fat, high sugar diet (HFHSD)^9^. The list of tissues taken and the time-course of experimentation is provided in supplementary table S1. The rationale behind the selection of the panel of the tissues was derived from the relevant literature and is described in the supplementary information^5^,^6^,^13^,^14^,^15^. The details of experimental strategy were recently reported by our group and is briefly described in supplementary information^9^,^10^. To study crosstalk between different tissues, we developed a novel analytical approach. We first downloaded the list of mouse gene products associated with the gene ontology category extracellular space (GO: 0005615) from the Amigo database. This provided us a list of 2445genes (Table S2). Next we downloaded the list of mouse gene products associated with the gene ontology category cell surface (GO: 0009986). We obtained a list of 3416 genes in this category (Table S2). Next, we queried the databases APID and InnateDB using PSICQUIC to list all possible interactions known between the secreted and cell surface bound molecules^11^. The PSIQUIC analysis revealed 2139 total interactions between these gene sets. Up till this point, the interactions could not distinguish whether it represented intercellular interaction (i.e. secreted molecule from same or different cell interacting with receptors at the cell membrane) or intracellular (i.e. interactions between the secreted and cell surface molecule within the same cell, for example in case of molecular interaction with the cytoplasmic region of a receptor). The latter, of course, could not be considered as a means of tissue crosstalk. Therefore to filter-out all these intracellular interactions, we manually curated all the interactions by scanning published literature and retained only those interactions which could be confirmed as intercellular. There were several obstacles initially as some of the well-known receptors also have isoforms that are secreted, thereby complicating the exclusion and inclusion criteria. In such cases, we classified them based on the broadly accepted function of those molecules. After manually filtering out, we were left with a set of 974 interactions that comprised of 357 receptors and 437 secreted molecules (Table S3). Further analysis of all these molecules in the expression data suggested that they were distinctly regulated between different tissues and displayed a wide variation in the expression dynamics over the course of the experiment (Fig S1). Next to understand possible tissue crosstalk, we applied a novel approach which is schematically shown in Fig. 1A. For each receptor-secreted molecule pair, we scanned the entire microarray data sets. Wherever both the receptor and secreted molecule were up-regulated (i.e. increased expression by two fold, compared to the control) at the same time point, the corresponding tissues were considered interacting at that time point. Depending on the tissues in question, the interactions were either autocrine or paracrine. As we monitored 10 different tissues, at any given time 100 distinct interaction pairs could be possible for one rec-sec pair. The entire set of res-sec interactions was thus analyzed to generate a comprehensive matrix of tissue crosstalk during the onset of obesity and diabetes in the mice (Table S4).

### Immediate early tissue crosstalks in animals fed on HFHSD identify molecular interactions previously known to be associated with obesity

The earliest time point comprised of 1, 6 and 14 days after the mice were put on HFHSD. We transformed the tissue crosstalk matrix into a network as shown in Fig. 1B (see methods and table S4 and S5). Intriguingly, within 24 hours of putting mice on HFHSD, a major regulatory cluster involving skeletal muscle, splenic macrophages and brown adipose tissues emerged. The three tissues showed remarkable crosstalk as well as significant autocrine regulations at day 1. At molecular levels, rec-sec pairs that contributed most to these interactions involved natriuretic peptides at the top with nearly 54 and 45 tissue pairs showing interaction involving NPPB to NPR2 and NPPB to NPR3, respectively. Although NPPB has been associated with cardiovascular homeostasis^16^, there are reports suggesting a strong negative correlation between serum natriuretic peptide and obesity and diabetes^16^. Since several tissues showed significantly enhanced expression of NPPB, it could very well qualify as a potential corrective measure getting activated early on upon consuming high calorie diet. This line of hypothesis gets further support from our subsequent analysis of the later time points where no significant NPPB mediated interaction was visible (Table S5). Other sec molecules showing very high crosstalk at day 1 were tenascin C (TNC), apolipoprotein B (APOB), Wnt5A, PNOC-ligand for the opioid receptor OPRL1 and NRG1. Each of these molecules has established clinical manifestations in case of obesity and diabetes^17^,^18^,^19^,^20^,^21^. Thus intriguingly through an unbiased method of studying tissue crosstalk in the diet induced obesity model, we were able to identify key regulators of systemic behaviour alterations at very early stages. Similarly, at day 6, infiltrating macrophages from subcutaneous, epididymal and brown adipose (SVC-SA, SVC-EA and SVC-BA) directed the majority of tissue crosstalk (Fig. 1B). Splenic macrophages, adipose BA and hippocampus were the major driving force of crosstalk at Day14. Interestingly, irrespective of the time points and the tissue combinations that were analyzed, most of the ligand-receptor pairs identified had close association with obesity and diabetes.

### Modulation of tissue crosstalk by corrective therapy using Kal-1 formulation

**Figure 2:**
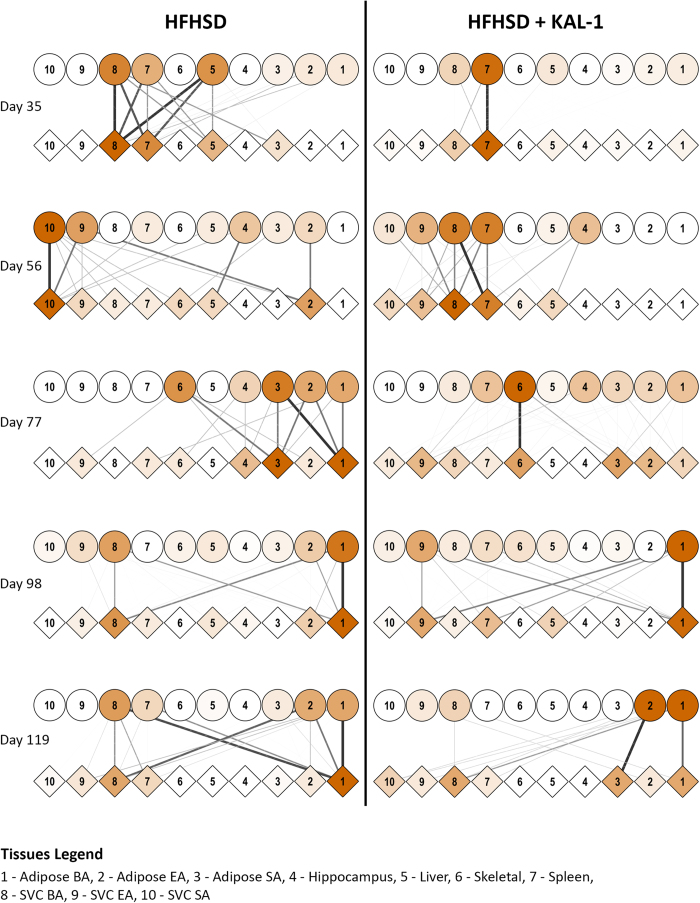
Tissue crosstalk at later stages of HFHSD and during rescue by Kal-1. Tissue crosstalk network, as described earlier, was generated during the later stages of putting mice on HFHSD. Kal-1 treatment was started 2 weeks after feeding mice on HFHSD. Since then, data were collected every three weeks (wk3, wk6, wk9, wk12 and wk15). However since experiment started 2 weeks before kal-1 treatment, the time points mentioned here include those 2wk as well. Thus 3wks became day 35, 6 wks day 56 and so on.

The efficacy of Kal-1 in controlling diet induced obesity and diabetes has been recently demonstrated by our group^9^. Kal-1 treatment was started 14 days after starting animals on HFHSD^9^. At every three weeks interval, microarray data from each of the above mentioned tissues under both HFHSD alone or in combination with Kal-1 was generated. We then repeated the same exercise as discussed above in Fig. 1B to assess the tissue crosstalk at later stages of diet induced obesity and its alterations upon corrective therapy with Kal-1 treatment. Figure 2 shows the comprehensive tissue crosstalks at 3, 6, 9, 12 and 15 weeks in mice fed on either HFHSD alone or in combination with Kal-1. At most time points HFHSD alone elicited more intricate tissue-tissue interactions compared to when Kal-1 was co-administered (Fig. 2). Taken together, our results suggest that the key inter-tissue communications were perturbed as a result of Kal-1 administration.The entire list of tissue interactions, counted for each pair of res-sec molecules, is provided in the table S4. A comparison of receptor-ligand pairs showing most frequent crosstalk is provided in the table S6. While some of these interactions were common between the two, a few were condition-specific. Thus MDK-LRP2, FGF2-FGFR2 and NRG1-ERBB3 were most frequent ligand-receptor pairs across most of the time points in both HFHSD and HFHSD+Kal1 animals (Table S6). LRP2 is a member of the low-density lipoprotein receptor family known for its association with familial hypercholesterolemia^22^,^23^. While apolipoproteins are their common ligand and believed to be associated with obesity and hyperlipidaemia, there are also several reports suggesting key role played by this family of receptors in regulating neural regeneration process^22^. MDK or midkine or neurite growth promoting factor 2 is a yet another ligand which have been putatively shown to interact with LRP2, possibly regulating its function in neuronal regeneration^24^. Thus, whereas MDK2:LRP2 interaction has high frequency in both HFHSD and HFHSD+Kal1 animals, APOB:LRP2 interaction figures at high frequency only among the HFHSD animals (Table S6). Another sec molecule that interacted with LRP2 in HFHSD was plasminogen activator urokinase or PLAU. LRP2 has been shown to interact with PLAU when in complex with plasminogen activator inhibitor (PAI1)^24^. Interestingly increased PAI1 levels have been shown to be associated with thrombosis, fibrosis as well as obesity and insulin resistance^25^. This particular interaction therefore highlights yet another inter-tissue communication that could facilitate disease development. Tissue crosstalk for apelin (APLN) and apelin receptor (APLNR) with high frequency in the HFHSD animals was intriguing. It has been shown that APLN can inhibit diet induced obesity by enhancing lymphatic and blood vessel integrity^26^.

### The protective effect of Kal-1 formulation potentially arises through immune modulation

**Figure 3:**
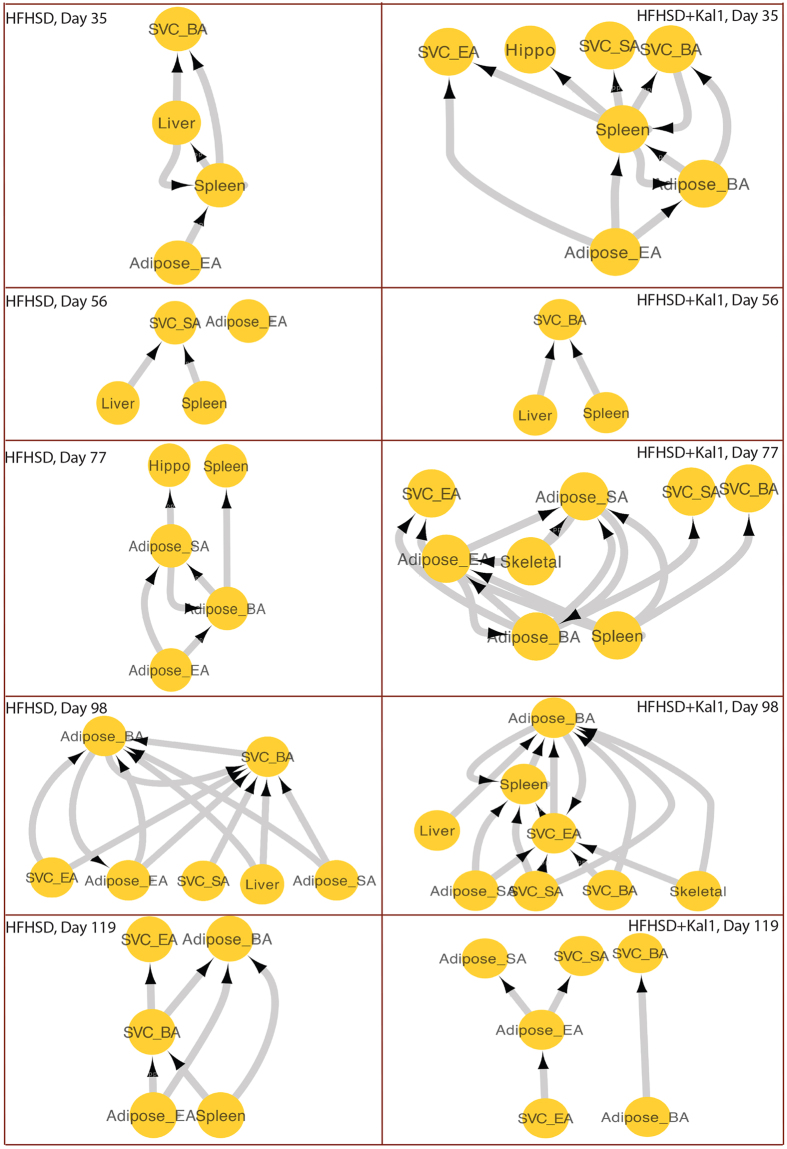
Hierarchical participation of different tissues in the cross talk. Tissues that participated in interactions shown in table S3 were analysed for their contribution as secretory tissue or receptor tissue. The in degree (arrow pointing towards) of different tissue was used to classify whether tissues participated as secretory (regulatory) or receiving (effector) tissue.

We next scrutinized the tissue participation among frequent receptor-ligand interaction pairs. The tissue that expresses “sec” was considered to be regulating the function of those that express “Res”. Thus the receptor-ligand interaction table were transformed on account of participating tissues rather than participating receptor-ligand pairs. The resulting interaction network was then analysed through hierarchical clustering to reveal tissues which received maximum signal (most arrows pointing towards them, at the top in the network) or those which released more sec molecules (arrows pointing away, at the bottom of the network). Several interesting patterns emerged through this analysis (Fig. 3). For example, more intricate tissue crosstalk was involved in HFHSD+Kal1 animals compared to HFHSD alone (83 compared to 67 respectively). Secondly, infiltrating macrophages like SVC_BA as well as SVC_EA and SVC_SA were major target tissues in most crosstalk across the time points in the Kal-1 treated animals (Fig. 3). At cumulative levels, the Adipose_BA was both a major source and most targeted tissue in the case of HFHSD whereas in the Kal-1 treated animals, the splenic macrophages were involved in maximum crosstalk^9^. Taken together it was apparent that the Kal-1 treated HFHSD animals recruited macrophage population from spleen as well as infiltrating macrophages from various adipose tissues in an intricate manner, raising the possibility that the protective effects of Kal-1 possibly arises through its immunomodulatory activities. This observation was in line with the results where it was shown that treatment with Kal-1 shifts the balance towards anti-inflammatory responses in the animals fed on HFHSD^9^. This could help control the chronic inflammatory state, typically associated with obesity and diabetes^9^.

### Regulation of inflammatory and metabolic related receptor-ligand genes

**Figure 4:**
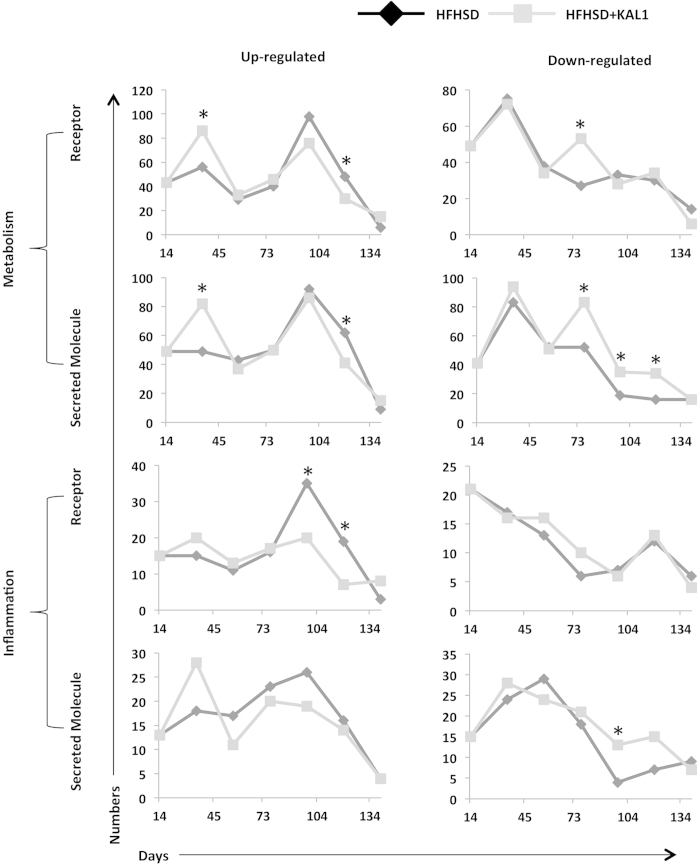
classification of receptor and ligand genes into metabolic and inflammation function. The list of genes from receptor and secretory molecules were classified into those which regulate metabolism or those affecting immune/inflammation using the standard gene ontology classification. At each time point, number of genes belonging to each of the categories up- or down-regulated is plotted in the figure. Astersiks represent significant differences (p-value < 0.05) in the statistical test comparing two population proportions (Table S7).

Having observed the distinct involvement of macrophages from spleen and adipose tissues in inter-tissue crosstalk, we asked how the res and sec genes studied here are distributed between these two biological function classes. Many of these molecules can be easily classified into immune regulators like cytokine and chemokine while some may regulate metabolism due to their influence on central metabolic pathways and anabolism. We classified res and sec genes into the two broad gene ontology classes: GO:0008152 for metabolism and GO:0006954 and GO:0006955 for immune and inflammation. Expression of all these genes across different tissues and time points was checked in the expression data to infer overall perturbation to these two functional classes (Fig S2). We then compared res and sec molecules regulation (up- or down-regulation) across the time points between HFHSD and HFHSD+Kal1 animals. As shown in Fig. 4, both HFHSD and HFHSD+Kal1 showed nearly identical profile under each category with little deviation. In HFHSD+Kal1 animals, more metabolic related res and sec genes were up-regulated at earlier time compared to HFHSD animals (Fig. 4). Interestingly, at later time points more metabolic receptor-ligand genes were down-regulated in HFHSD+Kal1 animals (Fig. 4). On the contrary, more inflammation related receptor-ligand genes were up-regulated in HFHSD animals at later time points (Fig. 4). On the similar note, more inflammation related receptor-ligand genes were down-regulated in HFHSD+Kal1 animals at later time points. Together these results hinted at the gradual tapering of inflammatory state in Kal1 treated animals, which therefore in addition to controlling weight gain also helped check the associated chronic inflammation.

### Dynamic analysis of expression data reveals tissue specific time-course trends

**Figure 5:**
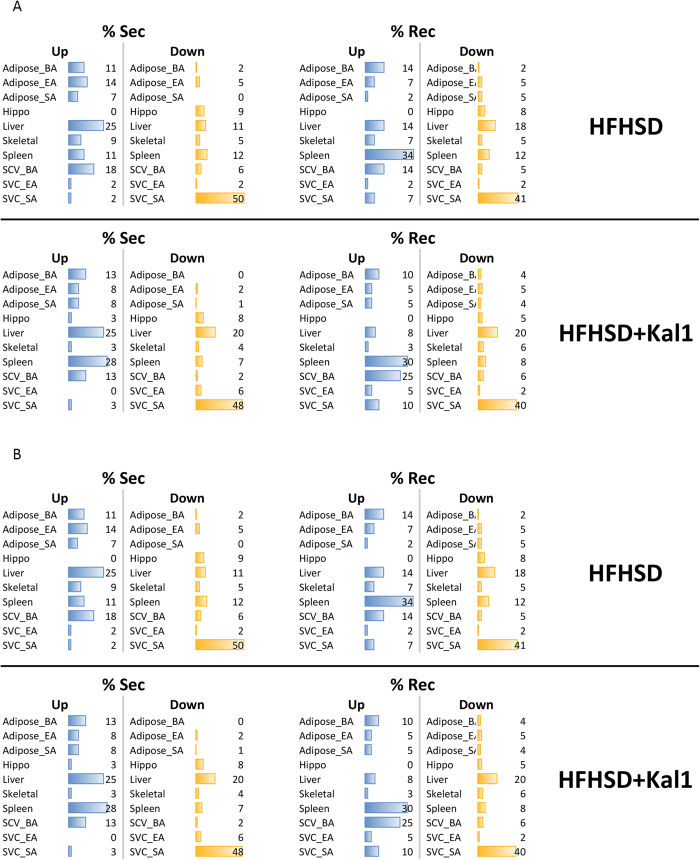
Dynamic tissue contribution. Dynamic tissue contribution in the expression of receptor and secretory molecules was calculated as described in the methods. The percentage tissue-wise contribution is represented in 5A. Corresponding analysis for those interactions where trends were opposite between Res and Sec molecules is shown in 5B. Table S8 shows p-values for the statistical test comparing two population proportions.

So far, our crosstalk analyses were time-point specific and independent of expression pattern at other time points during the study. We next decided to check the time course trend for expression of res and sec genes. The time-course trends took into account the changes in expression of a gene at any given time with respect to the preceding and following time points. Any pair was considered dynamically interacting when they followed exactly similar time-course trend (see methods for detail). We developed a simple algorithm to calculate the net change for each receptor-ligand pair and tissue participation in this interaction was catalogued (experimental methods). Figure 5 represents the percent res and sec genes that are up- or down-regulated during the entire experimental time-course. Through the dynamic analysis we observed, in HFHSD animals the liver showed increased expression of secretory molecule while the spleen showed highest receptor up-regulation (Fig. 5A). Interestingly, in the HFHSD+Kal1 animals too liver showed increased expression of secretory molecules (Fig. 5A). However unlike in HFHSD animals, spleen emerged as another tissue with more number of up-regulated secretory molecules in HFHSD+Kal1 animals (Fig. 5A). The tissue SVC-SA consistently showed most number of down-regulated genes in both res and sec categories and HFHSD or HFHSD+Kal1 treated animals (Fig. 5A). In the dynamic analysis we also considered Res-Sec pairs that showed opposing trend across the time course. Tissue distribution of such interactions under both combinations (Res-up; Sec-down and Res-down; Sec-up) are shown in Fig. 5B. The tissue distribution of opposing dynamic trend, signifying down-regulation or loss of interaction, was notably different between HFHSD and HDHSD+Kal1 animals. Specifically, higher participation of liver, SVC_SA and spleen in case of HFHSD animals was complemented by higher representation of adipose_BA and SVC_BA in the Kal-1 treated animals (Fig. 5B).

### Dynamic tissue crosstalk

**Figure 6:**
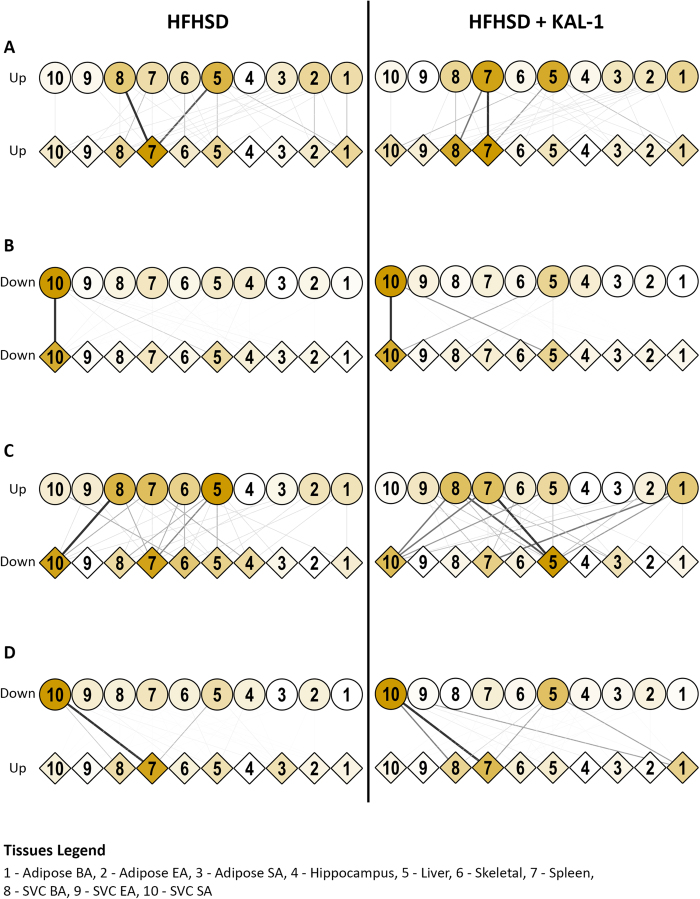
Dynamic tissue crosstalk during progression of obesity and control. Figure 6A shows dynamic tissue crosstalks established in HFHSD or HFHSD+Kal-1 groups as described in the text. Tissues expressing ligands are shown as circle while those expressing receptor are shown as diamond. Colour of nodes and thickness and colour of edges describe relative contribution/frequency in the tissue-crosstalk (see text). Tissue crosstalk where atleast one or both partners of res-sec pair were downregulated is shown in 6B, C and D as depicted.

The analysis above allowed us to extend the tissue crosstalk network in a dynamical manner. Thus those tissue pairs which showed exactly same time-course trend for sec and res respectively were considered to be communicating with each other. All such observed interactions between all the ten tissues are represented as tissue crosstalk network in Fig. 6A. It is important to note that the interactions mentioned here involved only those receptor-ligand pairs which had net up-regulation in the time-course trend. It emerged, under both the conditions, liver was the major regulator of tissue crosstalk, showing maximum number of sec genes getting up-regulated (Fig. 6A). In case of HFHSD, infiltrating macrophages from SVC_BA were yet another significant regulator of tissue crosstalk. The splenic macrophage received maximum crosstalk (high res up-regulation) in both the cases (Fig. 6A). However in the case of HFHSD+Kal1, the spleen was both the largest source as well as target of tissue crosstalk. Intriguingly, treatment with Kal1 showed infiltrating macrophages from SVC_BA as the major receptor expressing tissue (Fig. 6A). Thus Liver→spleen signal was constant in both cases while SVC_BA→spleen was specific for HFHSD. Moreover Spleen→Spleen and Spleen →SVC_BA were typical of HFHSD+Kal1. We initially thought down-regulated pairs of receptor-ligand should not be considered as interacting. However they provided intriguing opportunity to identify specific interactions that were lost or down-regulated during disease progression and control. We looked at the cases where Res was up while Sec was down or vice versa or where both res and sec were down. Tissues crosstalk for these conditions is shown in Fig. 6B–D. Interestingly crosstalk that were enriched in HFHSD animals like SVC_SA→spleen in Sec down Res up set, SVC_BA→SVC_SA in Sec up Res set and SVC_SA→ SVC_SA in Sec down Res down set were also present in HFHSD+Kal1 animals (Fig. 6B–D). However several other crosstalk were selectively enriched in the Kal-1 group alone like liver → adipose_BA in Sec down Res up set, SVC_BA→liver and spleen→liver in the Sec up Res down set (Fig. 6B,C). Similarly in the Sec down Res down set, two interactions (SVC_SA→liver and liver→SVC_SA) were highly enriched in only HFHSD+Kal1 group (Fig. 6D).

### Dynamic molecular crosstalk

**Figure 7:**
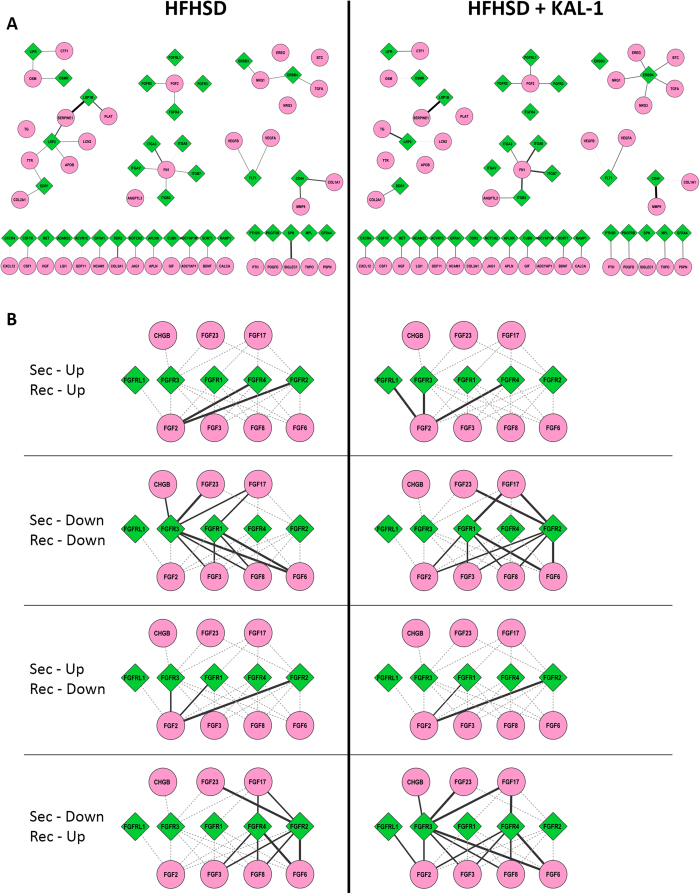
Dynamic molecular crosstalk. The molecular interactions contributing to the tissue crosstalk are depicted in 7A. Diamonds (green) are receptors while circles (pink) are secreted molecules. Thickness of the connections corresponds to number of tissues showing interaction involving the pair of receptor and secreted molecules. Figure 7B combines dynamic cross talk for the FGF signaling modules across conditions where eithr or both of Res and Sec genes were up- or down-regulated. Here too diamonds (green) are receptors, circles are secreted molecules and thickness of connections correspond to number of tissues showing mentioned interaction pattern involving the pair.

While tissue crosstalk patterns seemed intriguing, we went on to identify dynamic molecular crosstalks that were differentially regulated between the two conditions. The interactions lists were used to generate a network file which incorporated all the interactions in both the conditions. Then condition specific network was extracted to compare similarity and differences between the two conditions. Figure 7A shows dynamic receptor-ligand interactions that were up-regulated in HFHSD or HFHSD+Kal1. Some interactions were present in both cases like LRP1B-Serpine1, ITGA3-FN1, ERRB4-NRG1, FGFR2-FGF, LIFR-CTF1, CD44-MMP9, APLNR-APLN, SORT1-BDNF and GFRA1-NCAM1 (Fig. 7A). While interactions like LRP2-SERPINE1, LIFR-OSM, OSMR-OSM etc. were only seen in HFHSD animals (Fig. 7A). Similarly interactions like ITGA8-FN1, ITGB3-ANGPTL3, CXCR4-CXCL12 etc were specific to HFHSD+Kal1 only (Fig. 7A). The selectivity in crosstalk was intriguing, mostly involving receptor or ligand with known association to obesity or diabetes, however further mechanistic involvement could not be derived from these results.

We also looked at the interactions that were lost/down-regulated in HFHSD and Kal1 treated animals. The complete molecular crosstalk network covering all possible combintions of Res and Sec expression profiles are shown in figure S3. One key module that showed intriguing variability across various expression groups representing FGF signaling is shown in Fig. 7B. The myriad regulation of FGF signaling during obesity development and control is clearly demonstrated in this figure.

## Discussions

The present study was planned keeping in view the inclusive nature of obesity that affects several organs of the individuals resulting in insulin resistance and other metabolic and cardiovascular illness^2^. Adipocytes, which are at the centre of obesity studies, have emerged to have endocrine function-they secrete hormones and adipokines which have largely inflammatory influence and also effect overall lipid homeostasis in the body^6^,^7^. The molecular and cellular regulation of adipocyte physiology has therefore been investigated in detail. However mostly such studies fail to provide an integrative picture of how different tissues in the organism communicate with each other during disease progression^2^. For example, in individuals with excessive calorie uptake, how metabolic signal reaches the adipose tissues. Once adipose tissues start accumulating lipids, they secrete hormones like leptins and other cytokines which eventually lead to insulin resistance in skeletal muscle and elsewhere leading to development of diabetes. The recruitment of macrophages at the site of adipogenesis, the behavioural control of food uptake etc. are all intergral components of vital steps in the disease progression and involve communication between different cell-types^2^. However there is no such atlas available till date that captures all these communication chains between different tissues during the progression to obesity. In this study, we decided to generate the inter-tissue communication channels in a mouse model of diet-induced obesity. We captured changes in gene expression across ten different tissues which together represent vast majority of cell types involved in manifestation of obesity and associated pathology. In order to study inter-tissue crosstalk, we first developed a database of pairs of secreted molecules (ligands) versus cell surface molecules (receptor) that may interact inter-cellularly. Then through an indigenous algorithm we identified possible crosstalk between different tissues involving various ligand-receptor pairs. Such interactions are presumed to modulate the target tissue physiology which together with all such interactions across all the tissues manifests as obesity. The most intriguing aspect of this study was that the top interacting ligand-receptor pairs emerging through this unbiased analysis were all known regulators of diet-induced obesity associated physiology, thereby automatically confirming the validity of this approach. Interestingly, in addition to inter-tissue crosstalk our analysis also revealed considerable autocrine interactions (cognate res and sec expression going up in the same tissue) in tisssues like skeletal muscle, splenic macrophages, SVC_SA, Adipose_BA etc.

Inclusion of Kal-1 treated group in the study allowed further filtering of interactions that were specific to the disease progression^9^. There were more metabolic related interacting molecules expressed at early time points as compared to the untreated controls. Surprisingly this trend reversed at later time points, with Kal-1 treatment leading to down-regulation of metabolism related genes. Similarly HFHSD animals showed much higher expression of receptors associated with inflammation, again confirming to observed chronic inflammatory state in these animals^7^. The selectivity of interacting genes in the dynamic analysis revealed further insights. Thus Liver was the major source of secreted molecules/ligands, clearly highlighting the central metabolic role of this organ. While splenic macrophages expressed most receptor genes in both the conditions, higher receptor gene expression in SVC_BA in Kal-1 treated animals look fascinating. Role of brown adipose in energy dissipation is well documented^27^,^28^. While earlier mostly considered as a source of thermogenesis in neonates, it has been recently shown to be present in healthy adults^28^, thereby generating immense interest in its regulation which could provide means for controlling diet induced obesity. This result potentially provide basis for how in Kal-1 treated animals, both gain of mass and inflammation may be controlled. Increased involvement of macrophages infiltrating brown adipose tissue may significantly alter the overall lipid homeostasis in the organism. Finally, differential tissue crosstalk between the two conditions identified some key regulatory hubs and many of them like LRP2, FGFR, ITGA, ERBB etc. are already known to be involved in lipid homeostasis and obesity^4^.

In summary, we report here a comprehensive and dynamic inter-tissue crosstalk that gets estabished in the diet induced model of obesity in mouse. Using an unbiased approach we were able to filter-out specific inter- and intra-tissue crosstalk during progression of obesity and diabetes. Functional analysis of the observed crosstalk reiterated significant role played by inflammatory pathways in regulating the pathologies associated with obesity. The corrective therapy in addition to controlling the weight gain in animals, also mitigated the inflamamtory signaling reaffirming the importance of this pathway. Identification of differential tissue participation, specifically selective regulations in the brown adiposes and corresponding infiltrating macrophages certainly warrants further investigations as they could potentially provide oportunities for novel and unconventional intervention strategies.

## Additional Information

**How to cite this article**: Samdani, P. *et al.* A Comprehensive Inter-Tissue Cross-talk Analysis Underlying Progression and Control of Obesity and Diabetes. *Sci. Rep.* **5**, 12340; doi: 10.1038/srep12340 (2015).

## Supplementary Material

Supplementary Information
