# Supplementary material for: A Comprehensive Inter-Tissue Crosstalk Analysis Underlying Progression and Control of Obesity and Diabetes
Source: Sci Rep. 2015 Jul 23;5:12340. doi: 10.1038/srep12340 (PMC4511953; doi:10.1038/srep12340)
Supplement: Supplementary Information [file srep12340-s1.pdf]

Supplementary information for the manuscript

"A Comprehensive Inter-Tissue Cross-talk Analysis Underlying Progression and Control of Obesity and Diabetes"

Pawan Samdani[1], Meet Singhal[1], Neeraj Sinha[2], Parul Tripathi[2], Sachin Sharma[2], Kamiya Tikoo[2], Kanury VS Rao[2] and Dhiraj Kumar[2]*

[1] Department of Biochemical Engineering and Biotechnology, Indian Institute of Technology, Hauz Khas, New Delhi-110016

[2] Immunology Group, International Centre for Genetioc Engineering and Biotechnology, Aruna Asaf Ali Marg, New Delhi-110067

*Correspondence: dhiraj@icgeb.res.in

Serial No.	Supplementary Information	Title
1	Supplementary Table S1	List of tissues, experimental groups and time points
2	Supplementary Table S2	List of Receptors, Ligands and interactions among them
3	Supplementary Table S3	Manually curated list of receptors, ligands and interactions
4	Supplementary Table S4	Tissue level cross talk list
5	Supplementary Table S5	Molecular cross-talk list
6	Supplementary Table S6	List of receptor-ligand interactions occurring at high frequency
7	Supplementary Table S7	Test of significance for figure 4
8	Supplementary Table S8	Test of significance for figure 5A and 5B
10	Supplementary Figure S1	Expression heat-map of genes across tissues and time points
11	Supplementary Figure S2	Expression heat-map of genes belonging to inflammation and metabolism category
12	Supplementary Figure S3	Differential dynamic tissue cross-talk

Supplementary text:

Rationale behind selection of the tissues for this study

The list of tissues taken and the time-course of experimentation is provided in supplementary table S1. The rationale behind selection of the panel of the tissues was derived from available literature in the field. Thus adipose tissues, both white and brown were obvious tissues to monitor [1]. As obesity and associated conditions are believed to emerge due to low level of chronic inflammation which is partly attributed to the macrophages that infiltrate the adipose tissues, we also included infiltrating macrophages from each of the adipose tissues considered [2, 3]. Further, splenic macrophages were selected to understand systemic inflammatory status of the macrophages, liver samples were considered for their significant influence on metabolism and homeostasis. Skeletal muscle show diverse responses under conditions of obesity and obesity induced insulin resistance [4] whereas hippocampus was selected because of the role this part of the brain plays in food habits and cognition [5].

Supplementary methods:

Animals and diet

The details of experimental procedures were reported earlier [6, 7]. Briefly, all animal experiments were performed at BIONEEDS (Laboratory animals & Preclinical services) Bangalore, India, and approved by institutional animal ethics com- mittee (IAEC). BIONEEDS is approved by committee for the purpose of control and supervision of experiments on animals (CPCSEA), Ministry of forests and environments, Government of India. We used C57BL/6 J mouse as a model for diet induced diabetes and obesity. Mice were kept at 12:12 hr light: dark photoperiod with ad libitum access to food and water. Weaned mice at the age of 3-4 weeks were divided randomly into groups (each contains 30 animals). One group was fed on low fat diet (LFD, D12492, Research diets Inc.USA) with 10% of calories coming from fat. Another group was fed with HFHSD with 60% of calories coming from fat (D03062301, Research diets Inc.USA), and yet another group of mice were fed on HFHSD and supplemented with an ethano-botanical formulation, KAL-1 (75 µg of KAL-1). The mice were periodically monitored over an eighteen weeks period. At the interval of every 3 weeks, five animals from each group were sacrificed and different tissues were extracted and frozen in liquid nitrogen prior to RNA extraction.

Kal-1 treatment

Briefly, animals were fed on either normal diet/LFD or HFHSD. After two weeks of feeding animals on HFHSD, one group of animals were put on the herbal concoction named Kal-1 [7] while being fed on

HFHSD. At 0, 3, 6, 9, 12 and 15 weeks post Kal-1 treatment, animals were euthanized and a panel of tissues were harvested and quickly snap frozen in liquid nitrogen for further processing. From these samples, total RNA was isolated and microarray experiment was performed to profile transcriptional changes in these tissues as a function of time under HFHSD or HFHSD+Kal-1 treatment condition[7]. As an additional control to ensure the specificity of the effects of Kal-1 treatment, we had gavage controls for both the low-fat diet group and the high-fat diet group animals. The gavage control did not affect the phenotype of animals from either of the groups [7].

Tissue isolation, RNA isolation and microarray

Table S1 shows the list of tissues isolated and processed. In brief, Hippocampus, Liver, and Skeletal Muscle tissues were isolated and immediately snap frozen for subsequent RNA isolation. Additionally, Splenic macrophages, Brown Adipose (BA), White Adipose Epididymis (EA), White adipose Subcutaneous (SA), Infiltrating macrophages from SVC BA, SVC EA and SVC SA, respectively were also isolated using protocol described earlier[3]. Briefly SVC macrophages and adipocyte tissues were further processed separately using following protocols- adipose tissues (BA, EA and SA regions) were isolated, weighed and then collagenase solution was added (3 ml/g of the tissue). Tissues were homogenized and the tissue solution was kept in shaking water bath at 37°C for 45 minutes. After centrifugation at 3600 rpm for 20 minutes, the pellet was treated with erythrocyte lysis buffer and adipocytes layer was saved for further processing. The treated pellet was centrifuged at 3500rpm/10 min and the resulting pellet was dissolved in 1x PBS as SVC. The adipocytes layer obtained was further processed with collagenase and incubated at 37°C for 30 minutes in shaking water bath. After centrifugation at 3600 rpm for 20 min pellet was obtained and was kept as adipocytes. The SVC macrophages and splenic macrophages were isolated by magnetic separation using anti-CD11b antibody. The SVC macrophages and adipocytes for all the tissues were frozen and sent for RNA extraction. For every tissue and time points, microarray experiment was performed from three different animal independentally.

Microrray data analysis

The overall strategy of data normalization and finally getting a non-redundant set of genes that were differentially regulated has been described in our recent study [6]. Fold changes calculated for any gene in HFHSD or HFHSD+Kal1 animal tissues were normalized against corresponding expression values in the LFD mice. This helped filter out age related changes in gene expression and thereby allowing analysis of only those genes that were directly perturbed during obesity and diabetes progression.

Supplementary Table S1: List of tissues, experimental groups and time points

LFC: Low Fat Control, HFHSD: High Fat High Sugar Diet, Kal1: ethno-botanical formulation.

Supplementary Table S2: List of Receptors, Ligands and interactions among them

This table contains list of molecules (Sheet1 and Sheet2) that were downloaded from the databases and interactions (Sheet 3) among them. Many interactions in this table represented intra-cellular interactions.

Supplementary Table S3: Manually curated list of receptors, ligands and interactions

To filter-out only genuine interactions between ligands and cell surface receptors, we manually searched for each interaction in different databases and experimental details.

Supplementary Table S4: Tissue level cross talk list

Tissue cross-talk and participating receptor-ligand pairs are provided here in different worksheets (named accordingly). Please see methods for the details.

Supplementary Table S5: Molecular cross-talk list

For the number of tissue pairs, a given receptor-ligand (Res-Sec) interaction was observed through our analysis is represented here across the time points of the study. Since there are 10 different tissues, for any given res-sec pair, a maximum of 10^2 i.e. 100 possible interactions are possible.

Supplementary Table S6: List of receptor-ligand interactions occurring at high frequency

Receptor-ligand pairs that showed interaction at three or more than three time-points are listed in the table under HFHSD and HFHSD+Kal-1 conditions. Those highlighted in yellow are common between both the conditions.

Supplementary Table S7: Test of significance for the data in figure 4

As desribed in the methods, hypothesis testing for two population proportion was conducted to test statistical significance in the data presented in figure 4.

Supplementary Table S8: Test of significance for the data in figure 5

As desribed in the methods, hypothesis testing for two population proportion was conducted to test statistical significance in the data presented in figure 5.

Supplementary Figure S1: Expression heat-map of genes across tissues and time points

Heat-maps capturing expression profile of receptors (S1A) and ligands (S1B) in HFHSD and HFHSD+Kal1 (S1C and S1D) group are presented here.

Supplementary Figure S2: Expression heat-map of genes belonging to inflammation and metabolism category

Heat-maps capturing expression profiles of receptors and ligands in HFHSD and HFHSD+Kal1 belonging to inflammatory (S2A-S2D) and metabolism (S2E-S2H) classes are presented here.

Supplementary Figure S3: Differential dynamic tissue cross-talk

For this analysis, along with up-regulated interactions, those interactions were also included where significant down-regulation of receptor, secreted molecule or both takes place in HFHSD+Kal-1 as compared to HFHSD. Here too, green diamonds represent receptors and pink circles represent secreted molecules. Solid lines suggest increased cross-talk in HFHSD+Kal1 as compared to HFHSD alone. Dotted lines represent decreased cross-talk in HSHD+Kal1 as compared to HFHSD alone.

Supplementary References

1. Rosen, E.D. & Spiegelman, B.M. Adipocytes as regulators of energy balance and glucose homeostasis. *Nature* **444**, 847-853 (2006).
2. Gregor, M.F. & Hotamisligil, G.S. Inflammatory mechanisms in obesity. *Annu Rev Immunol* **29**, 415-445 (2011).
3. Weisberg, S.P. et al. Obesity is associated with macrophage accumulation in adipose tissue. *The Journal of clinical investigation* **112**, 1796-1808 (2003).
4. Eckardt, K., Taube, A. & Eckel, J. Obesity-associated insulin resistance in skeletal muscle: role of lipid accumulation and physical inactivity. *Rev Endocr Metab Disord* **12**, 163-172 (2011).
5. Martin, A.A. & Davidson, T.L. Human cognitive function and the obesogenic environment. *Physiol Behav* (2014).
6. Sinha, N., Sharma, S., Tripathi, P., Negi, S.K., Tikoo, K., Kumar, D., Rao, K. and Chatterjee, S. Molecular Signatures for Obesity and Associated Disorders Identified Through Partial Least Square Regression Models. *BMC Systems Biology* **In Press** (2014).
7. Tikoo, K., Misra, S., Rao, K.V., Tripathi, P. & Sharma, S. Immunomodulatory Role of an Ayurvedic Formulation on Imbalanced Immunometabolics during Inflammatory Responses of Obesity and Prediabetic Disease. *Evid Based Complement Alternat Med* **2013**, 795072 (2013).

Figure S1–A : Heatmap of receptors (HFHSD)

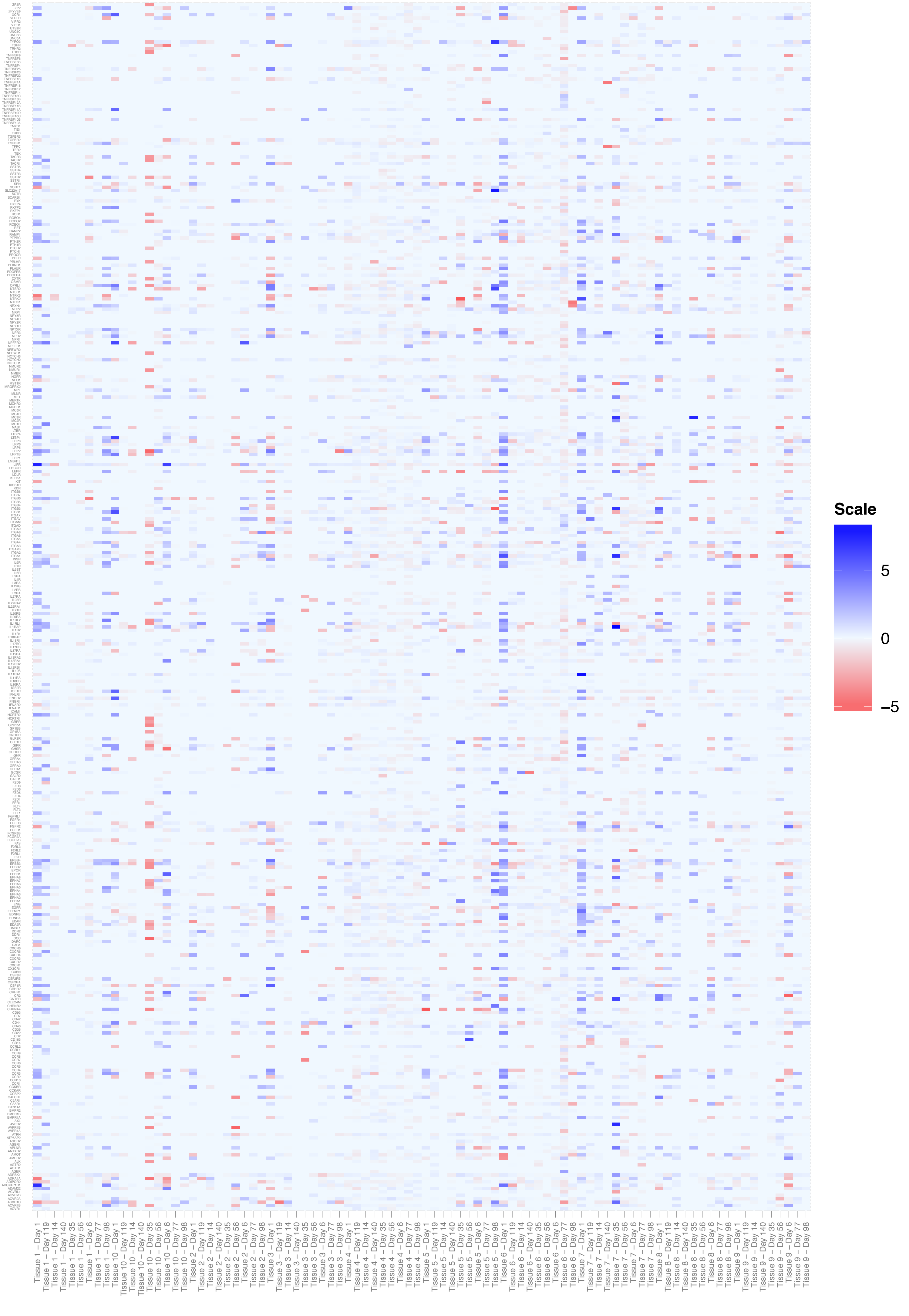

Figure S1−B : Heatmap of secretory (HFHSD)

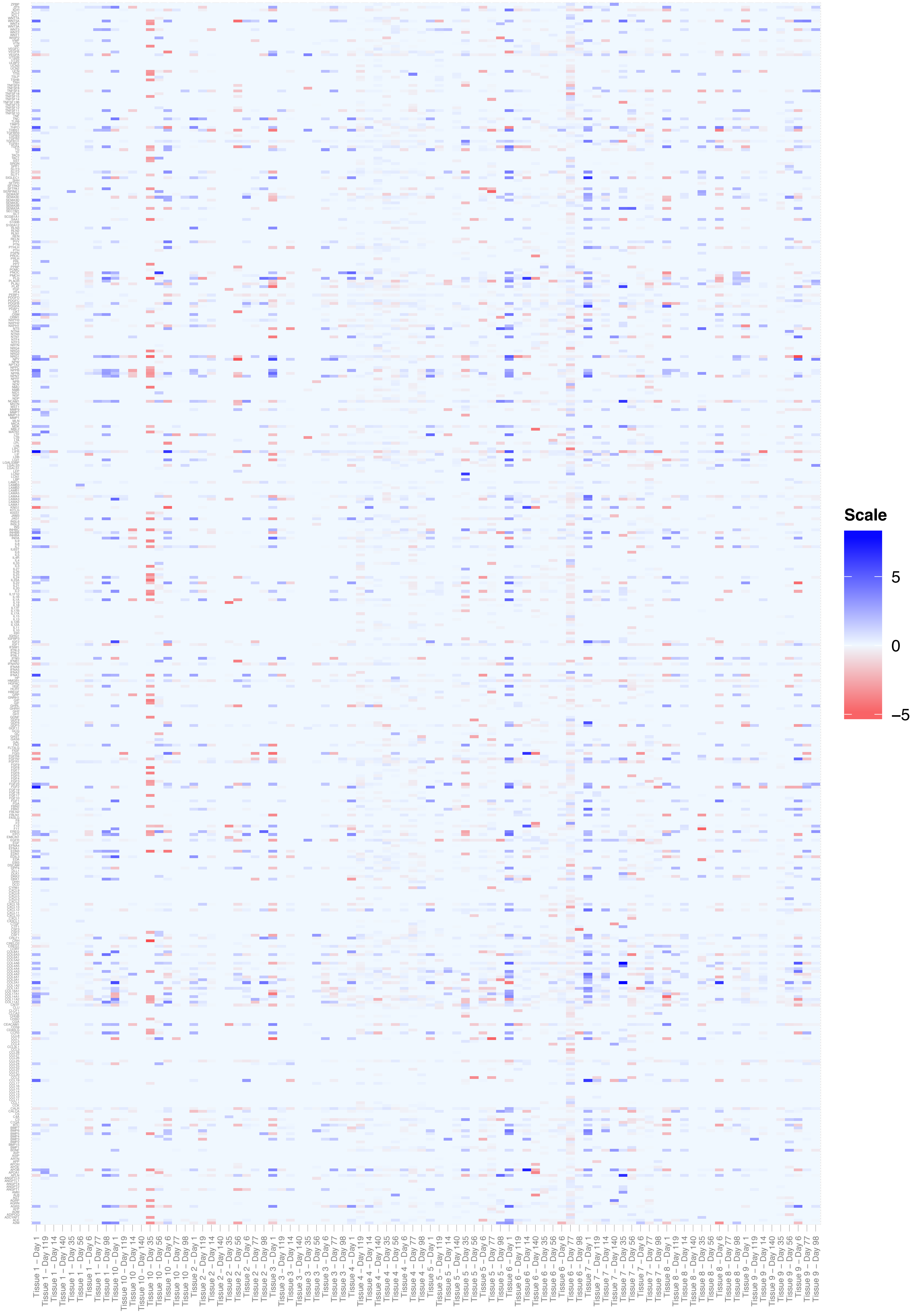

Figure S1−C : Heatmap of receptor (HFHSD+Kal1)

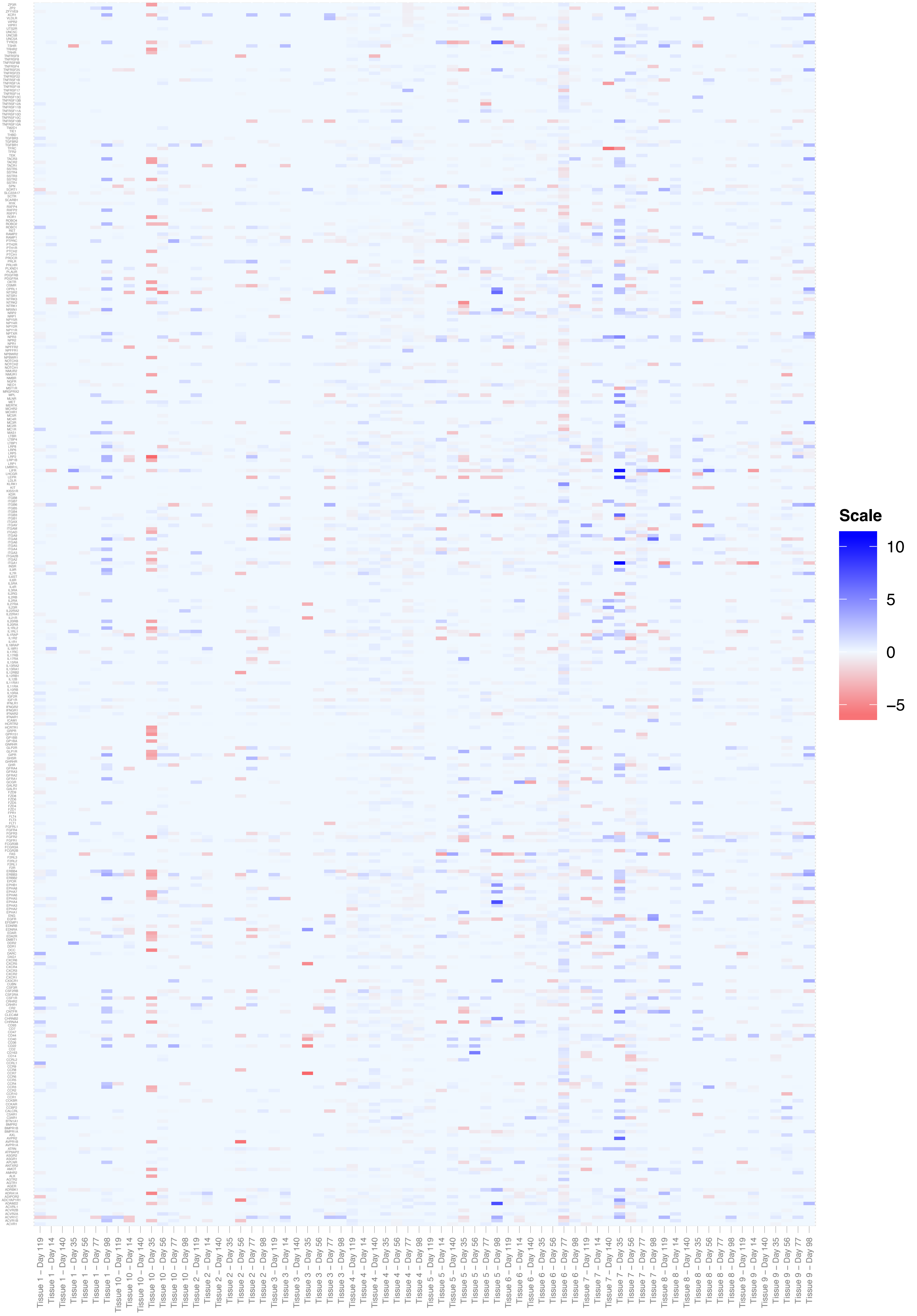

Figure S1−D : Heatmap of secretory (HFHSD+Kal1)

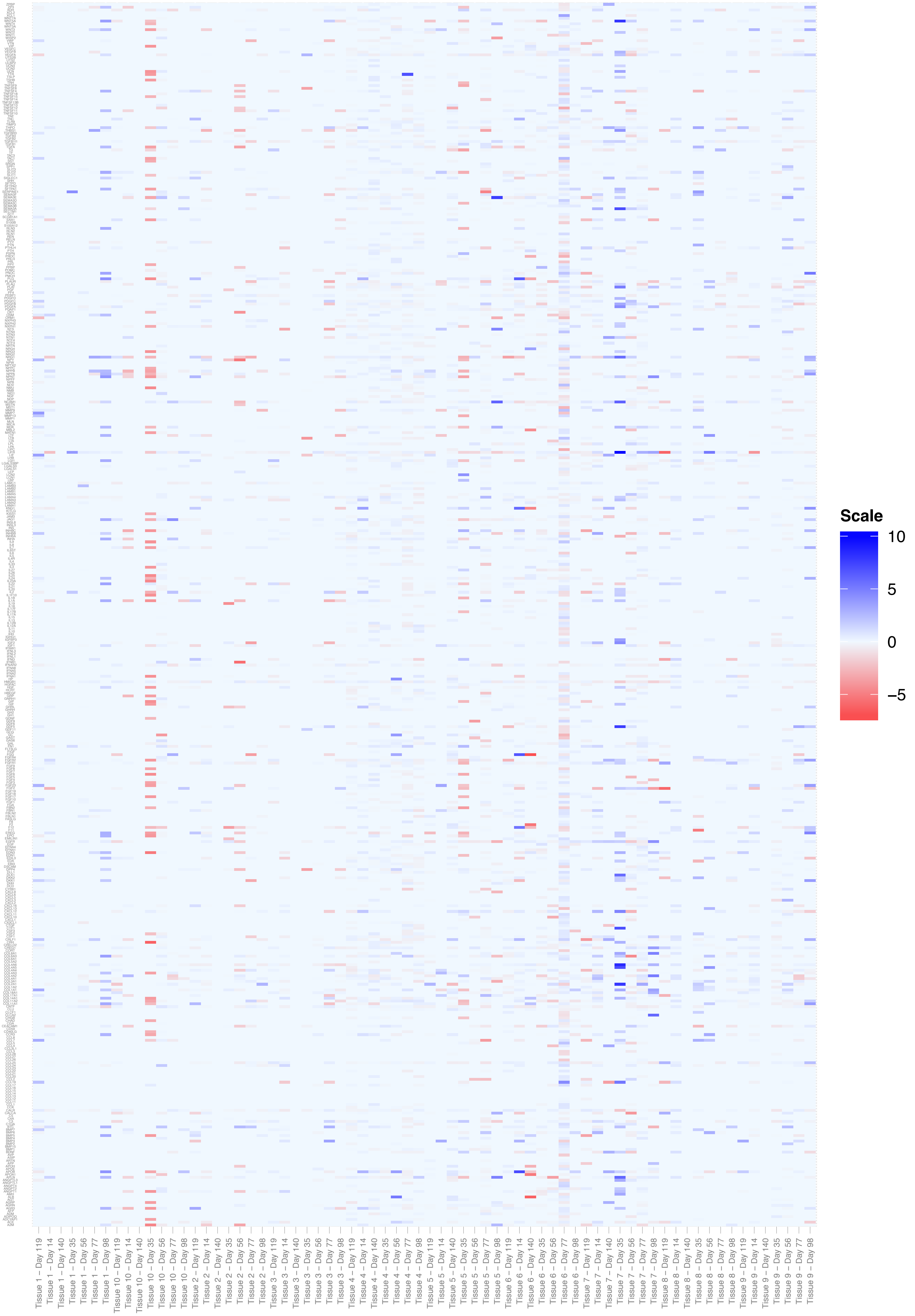

Figure S2–A : Heatmap of inflammatory receptor (HFHSD)

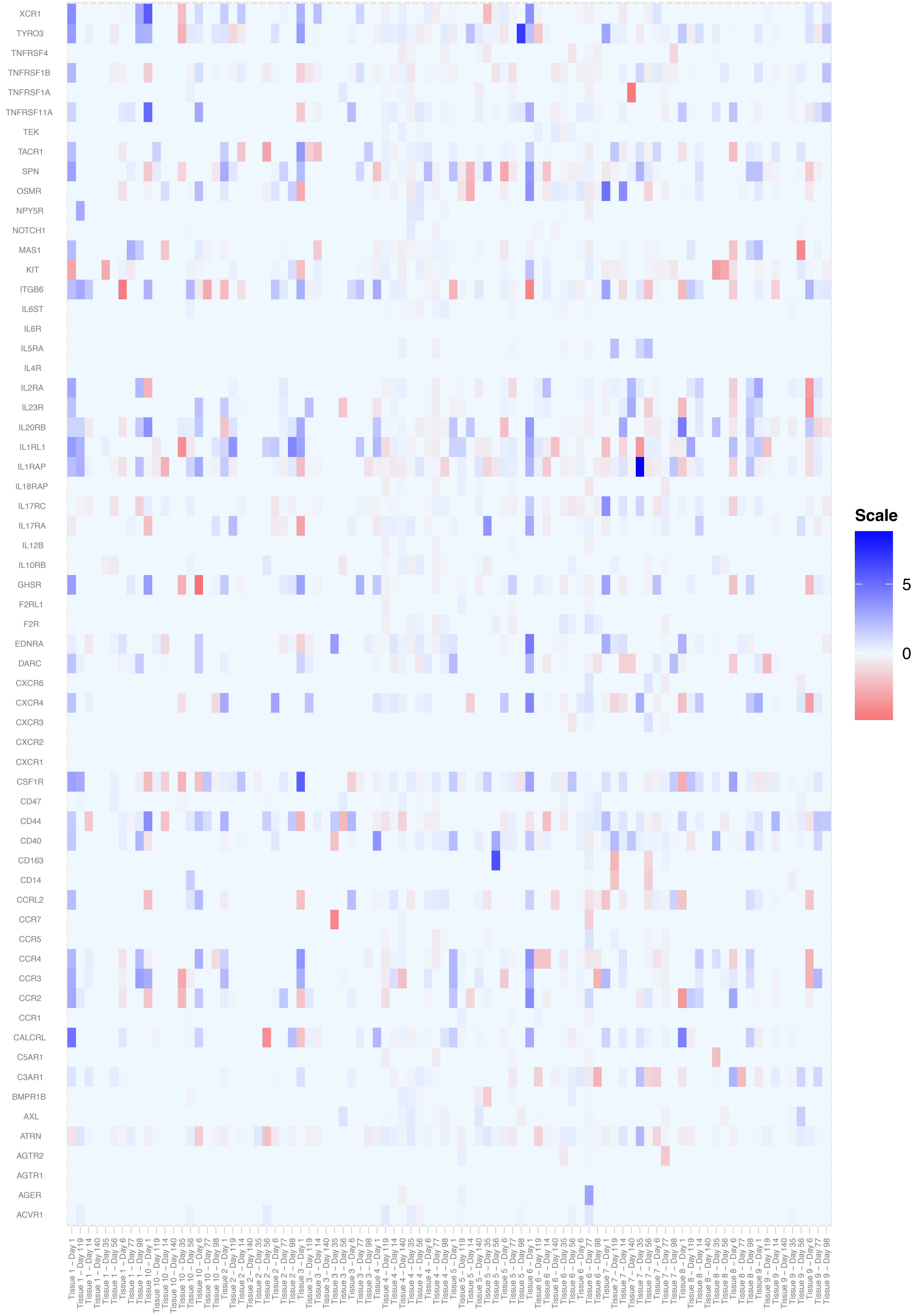

Figure S2–B : Heatmap of inflammatory secretory (HFHSD)

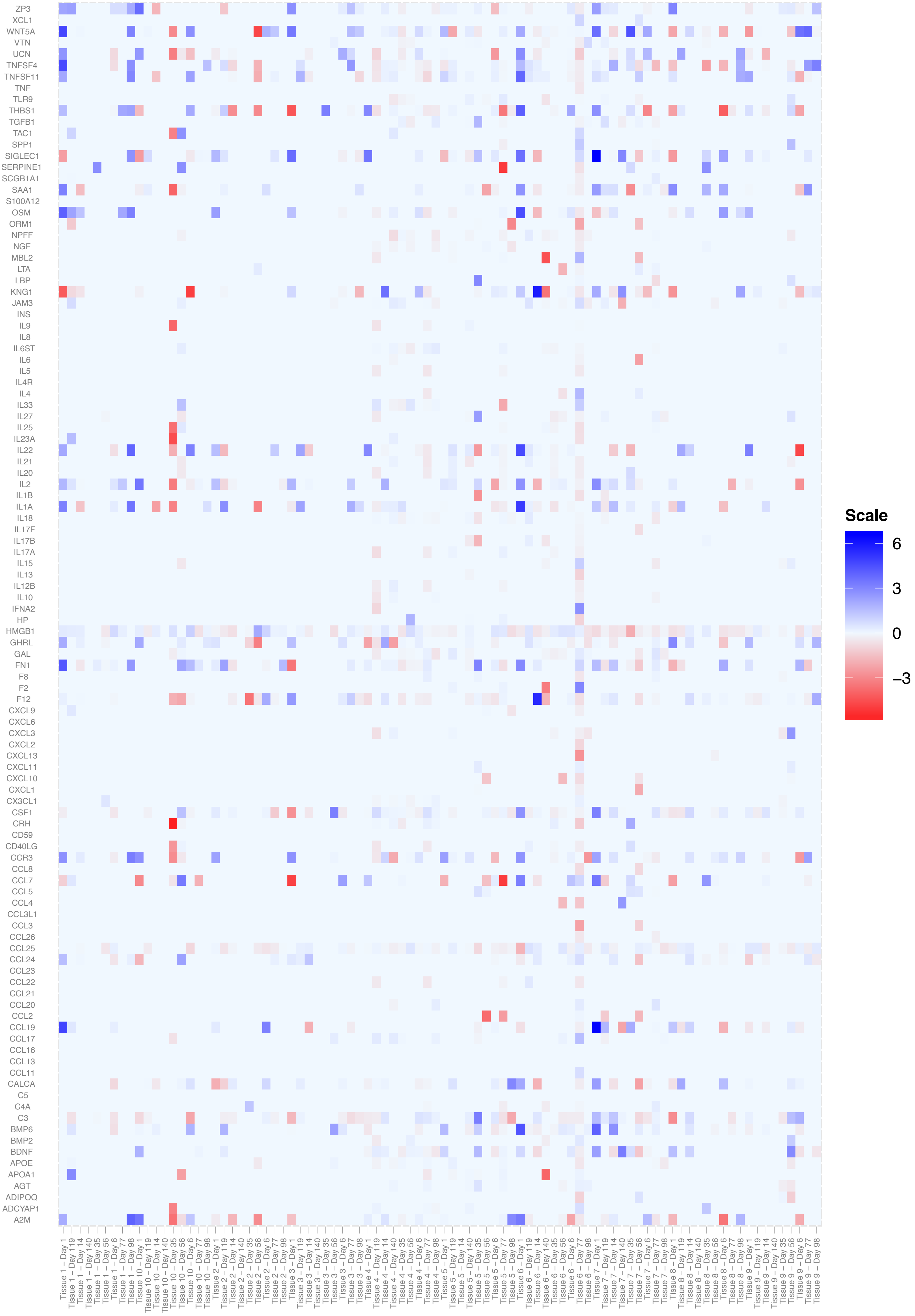

Figure S2−C : Heatmap of inflammatory receptor (HFHSD+Kal1)

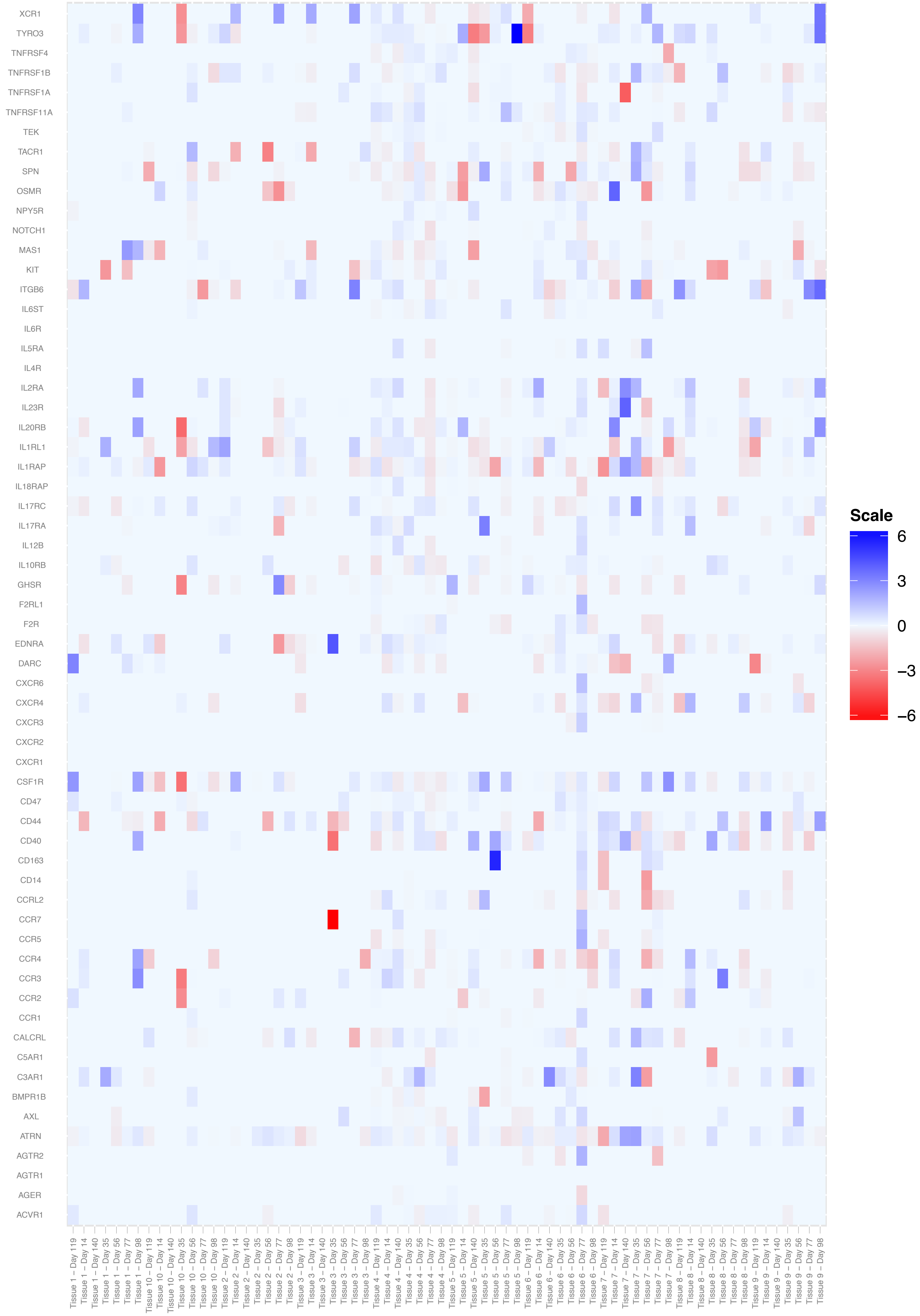

Figure S2-D : Heatmap of inflammatory secretory (HFHSD+Kal1)

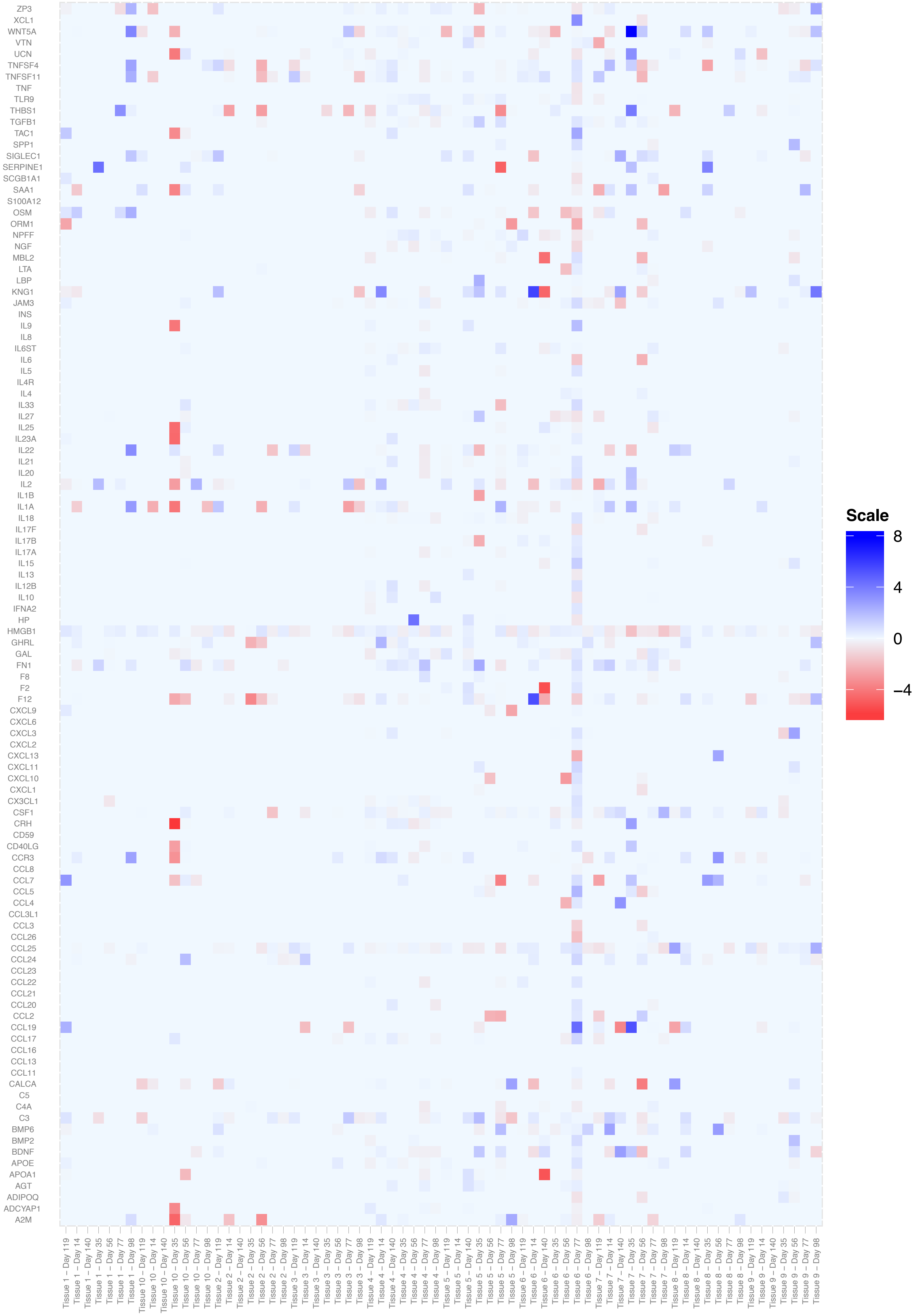

Figure S2−E : Heatmap of metabolism receptor (HFHSD)

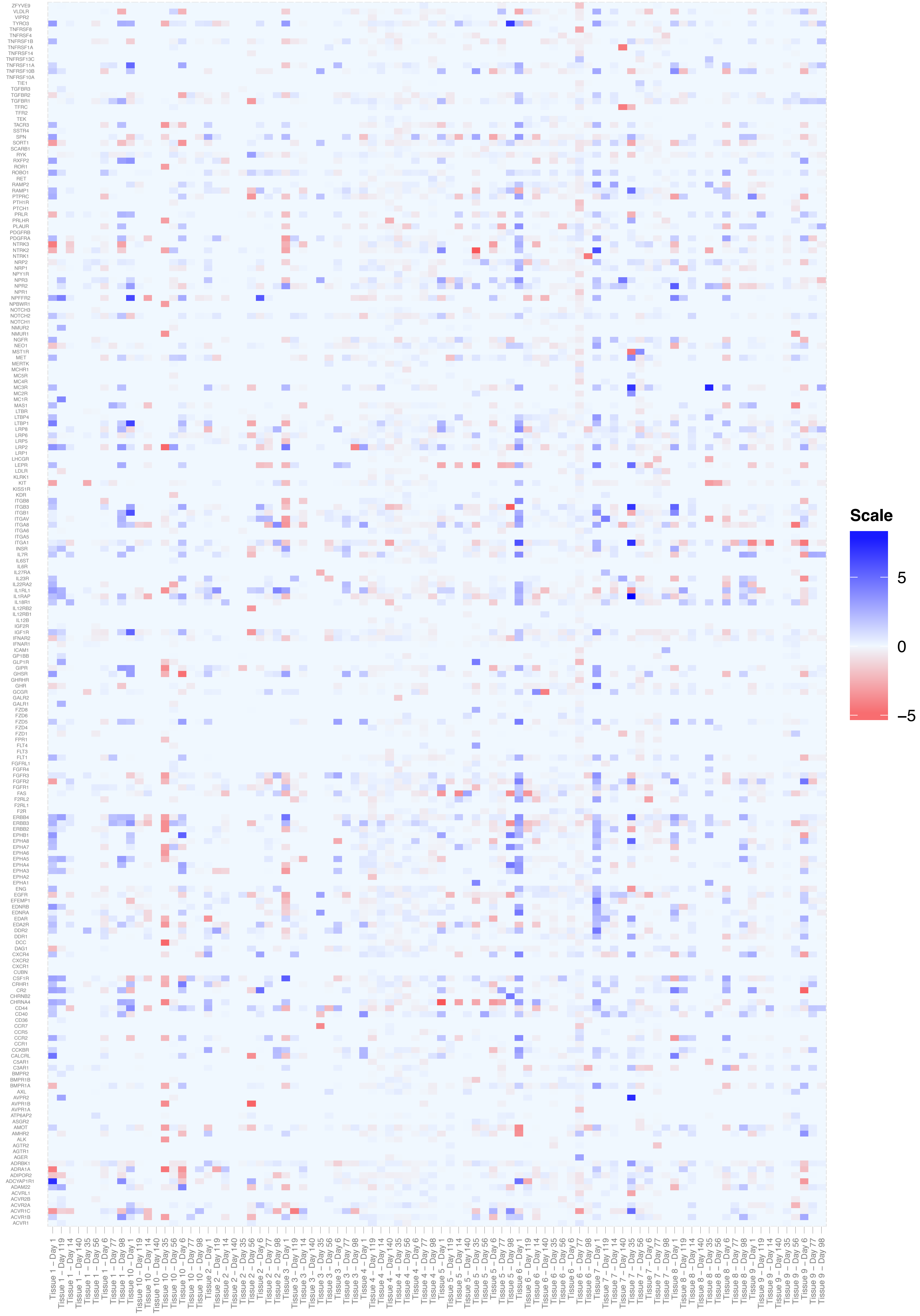

Figure S2−F : Heatmap of metabolism secretory (HFHSD)

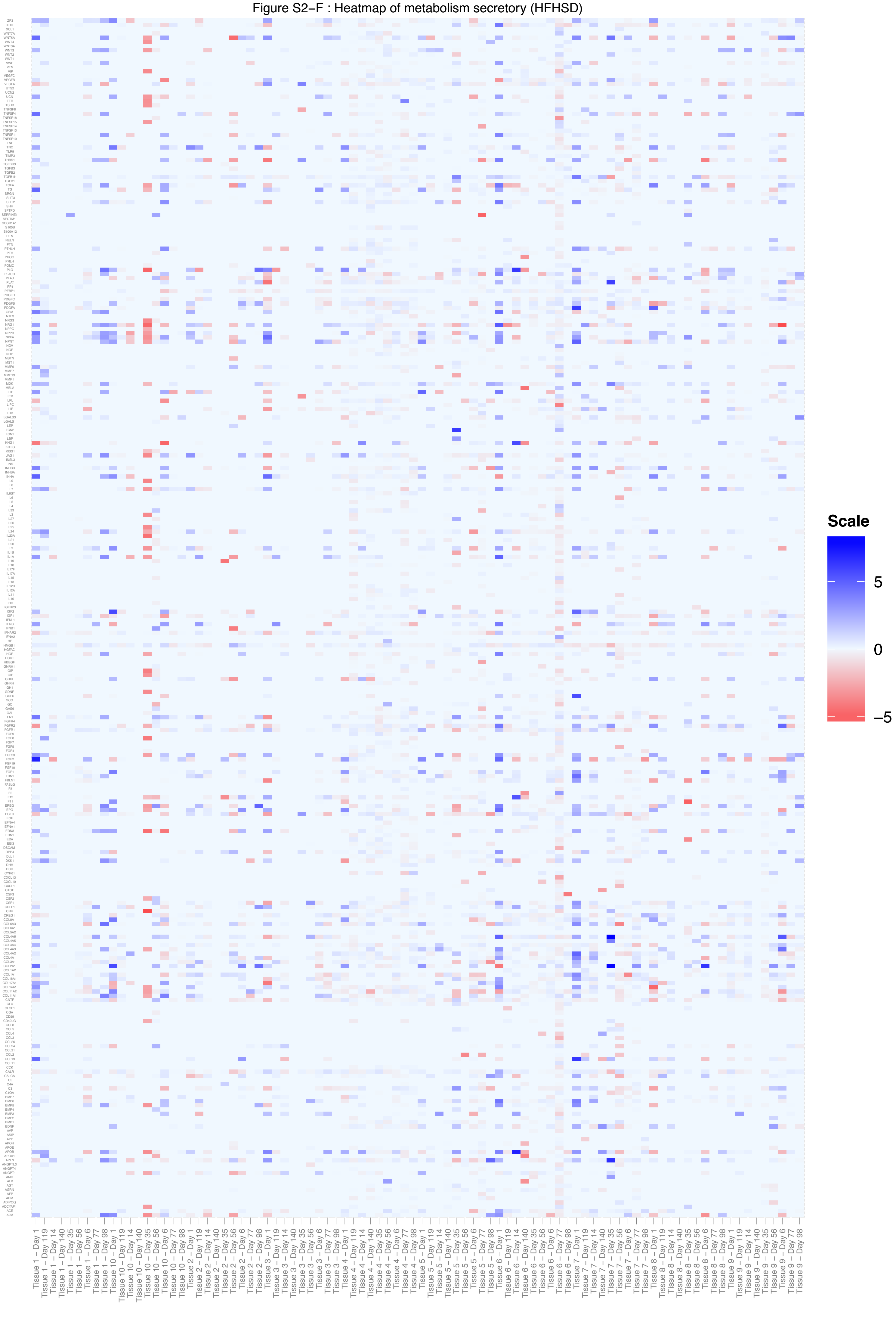

Figure S2−G : Heatmap of metabolism receptor (HFHSD+Kal1)

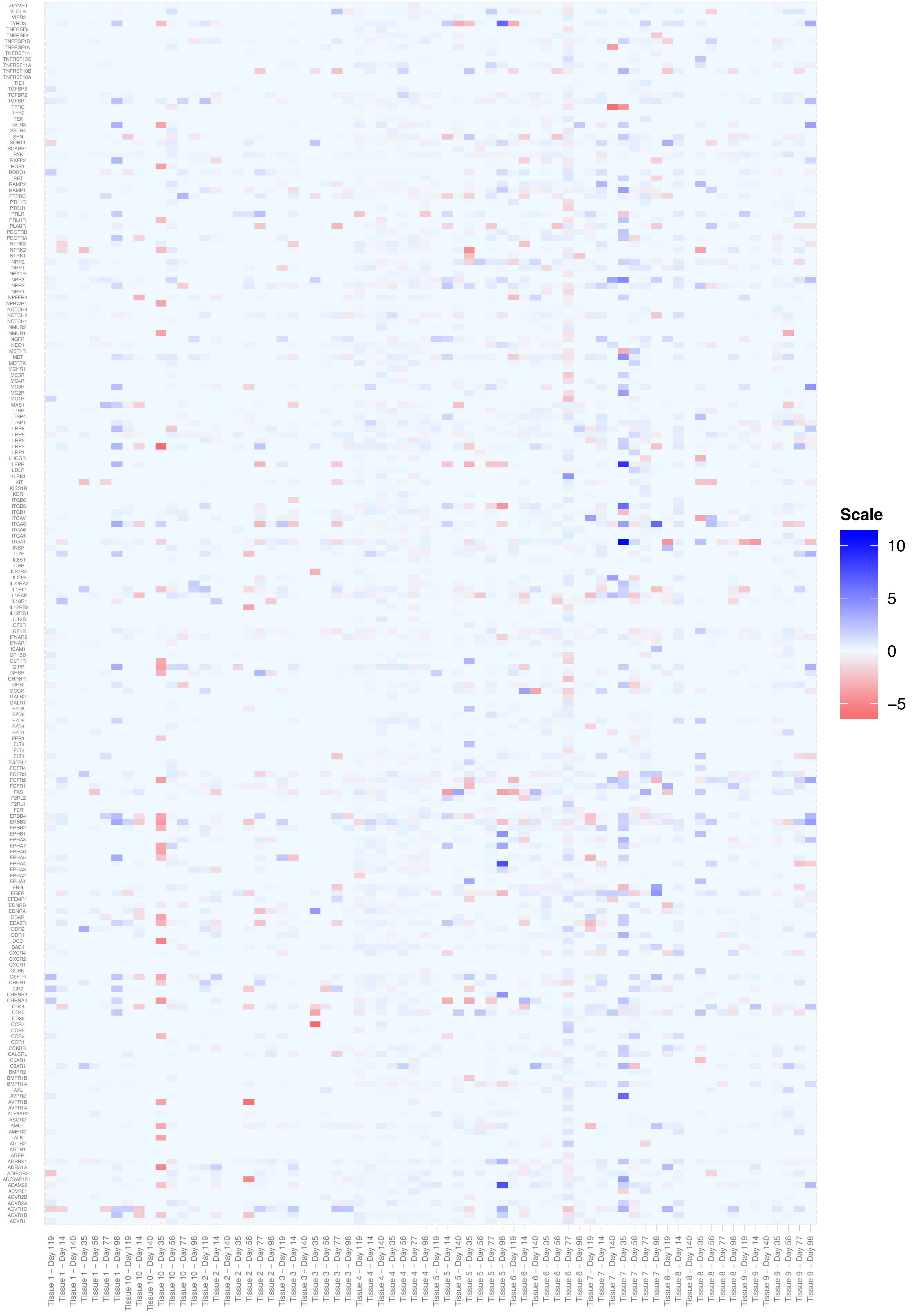

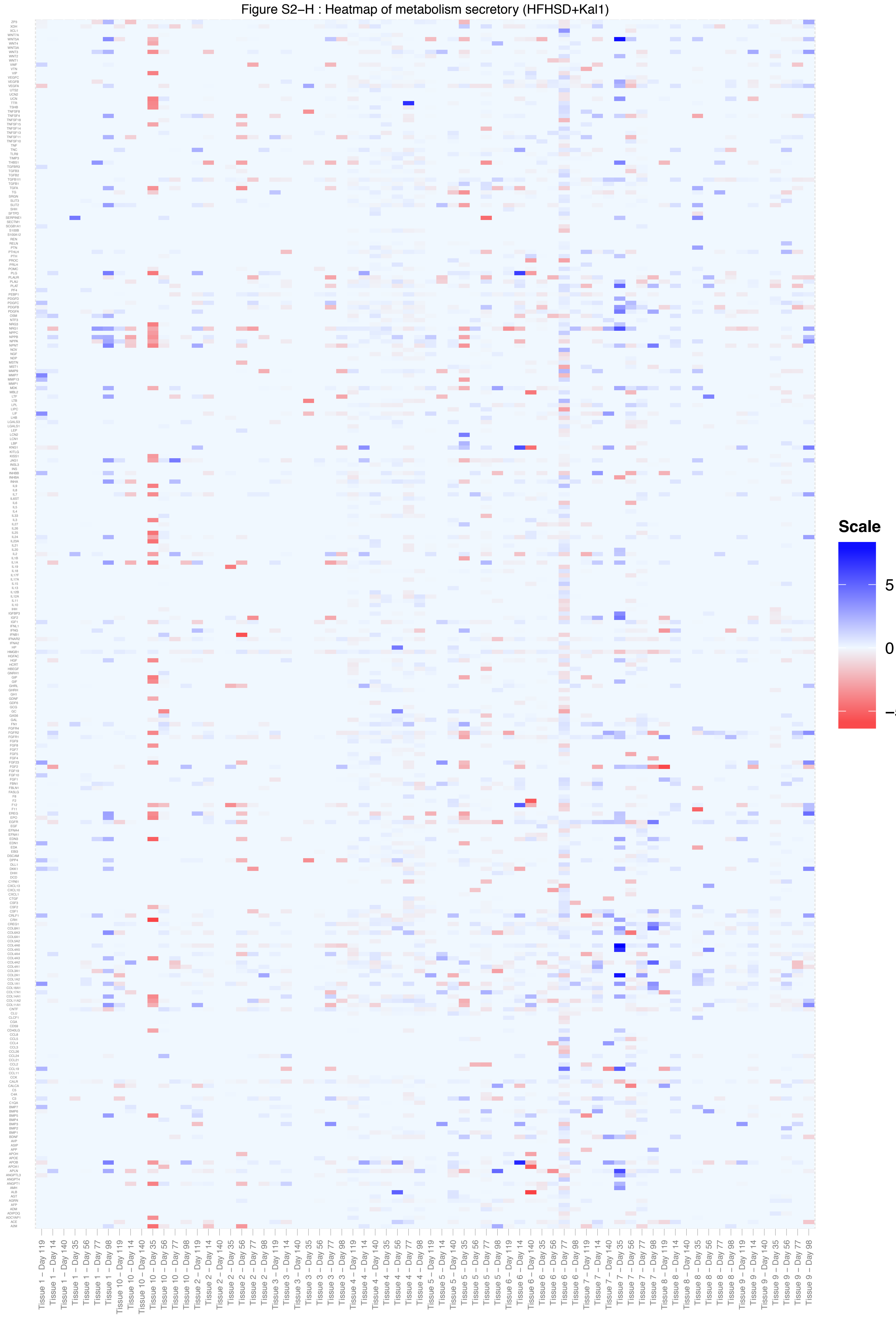

Figure S2−H : Heatmap of metabolism secretory (HFHSD+Kal1)
Scale
5
0
−5

Supplementary Figure S3A

HFC: Sec-Down, Res-Down

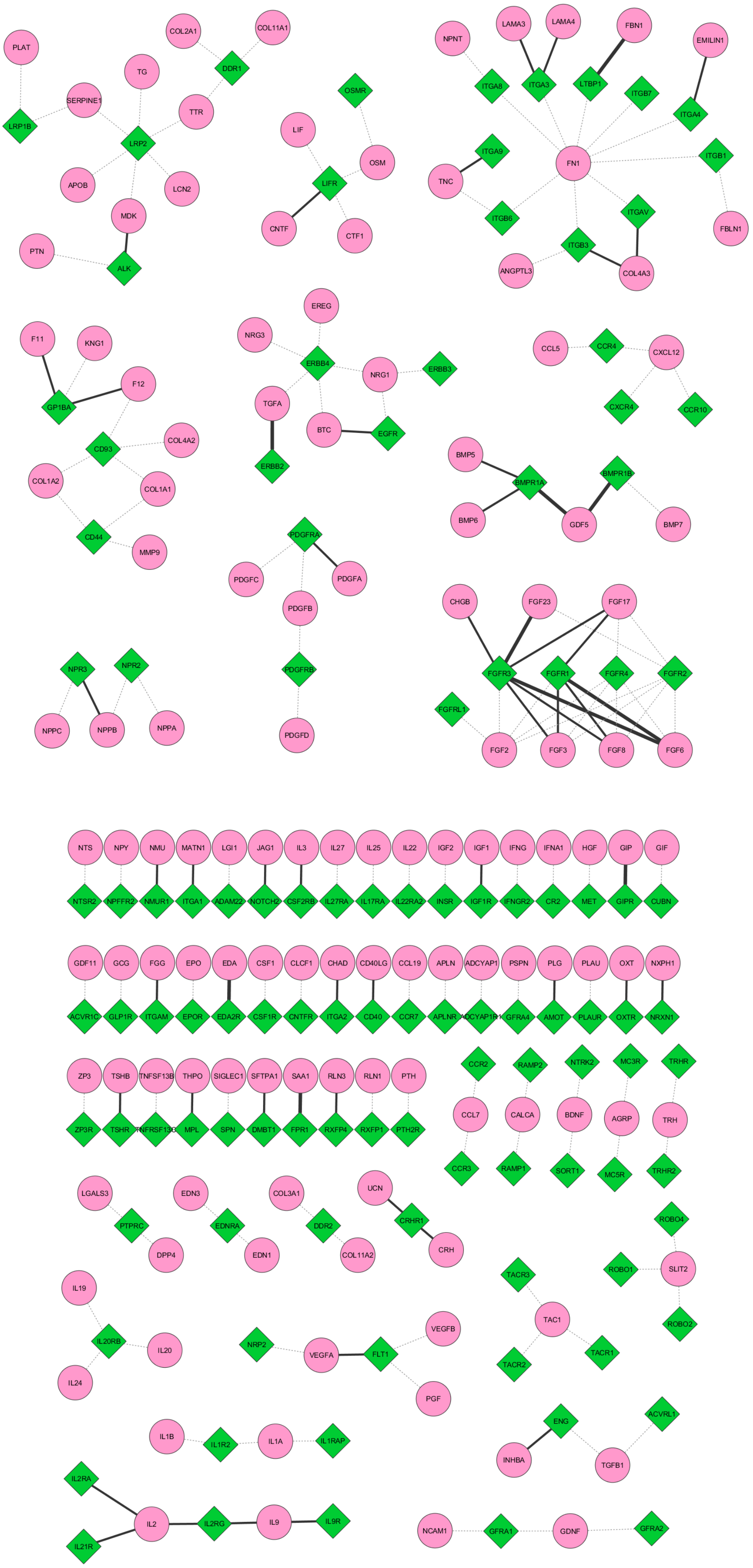

Supplementary Figure S3B

HFC: Sec-Down, Res-Up

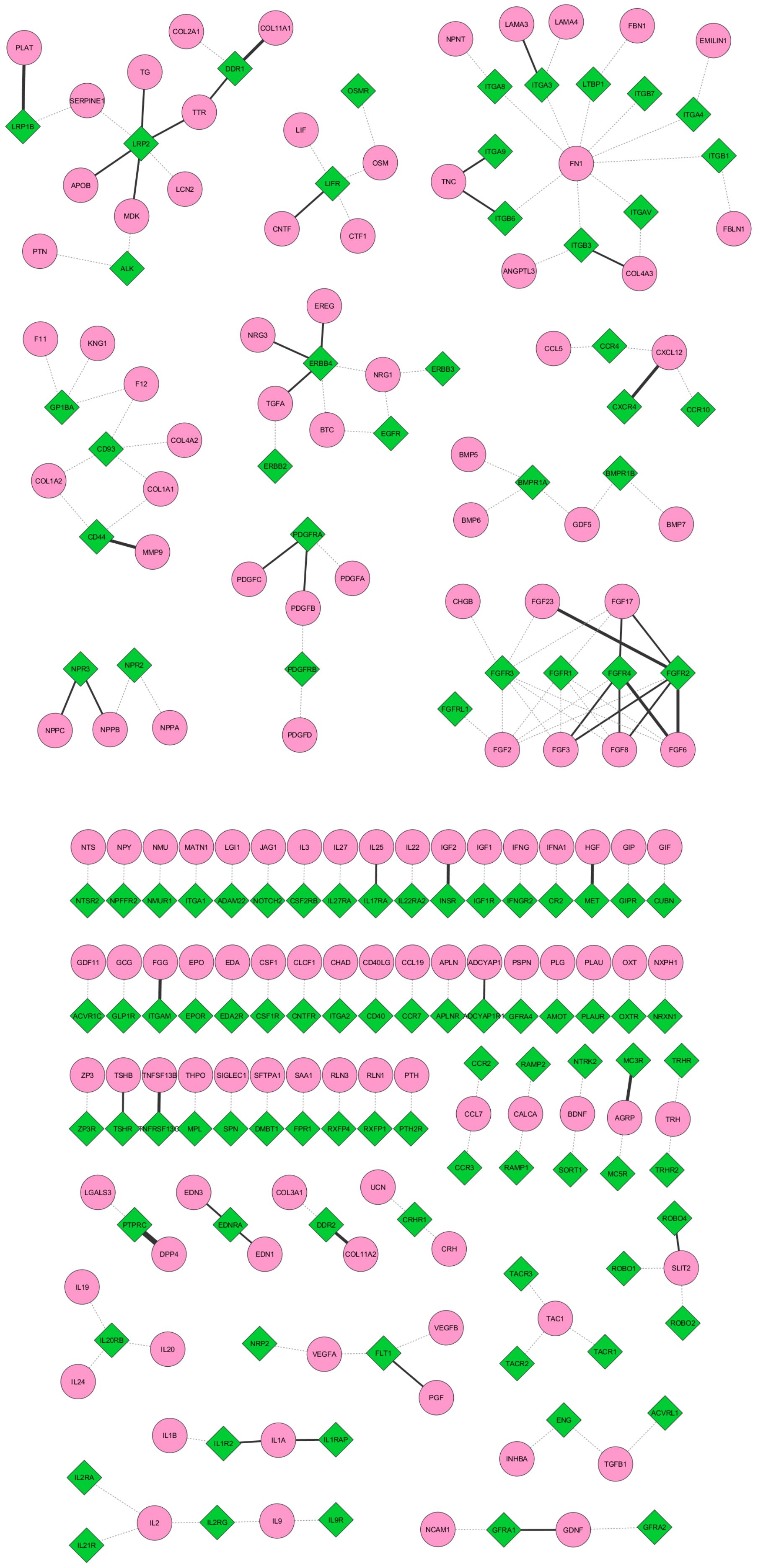

Supplementary Figure S3C

HFC: Sec-Up, Res-Down

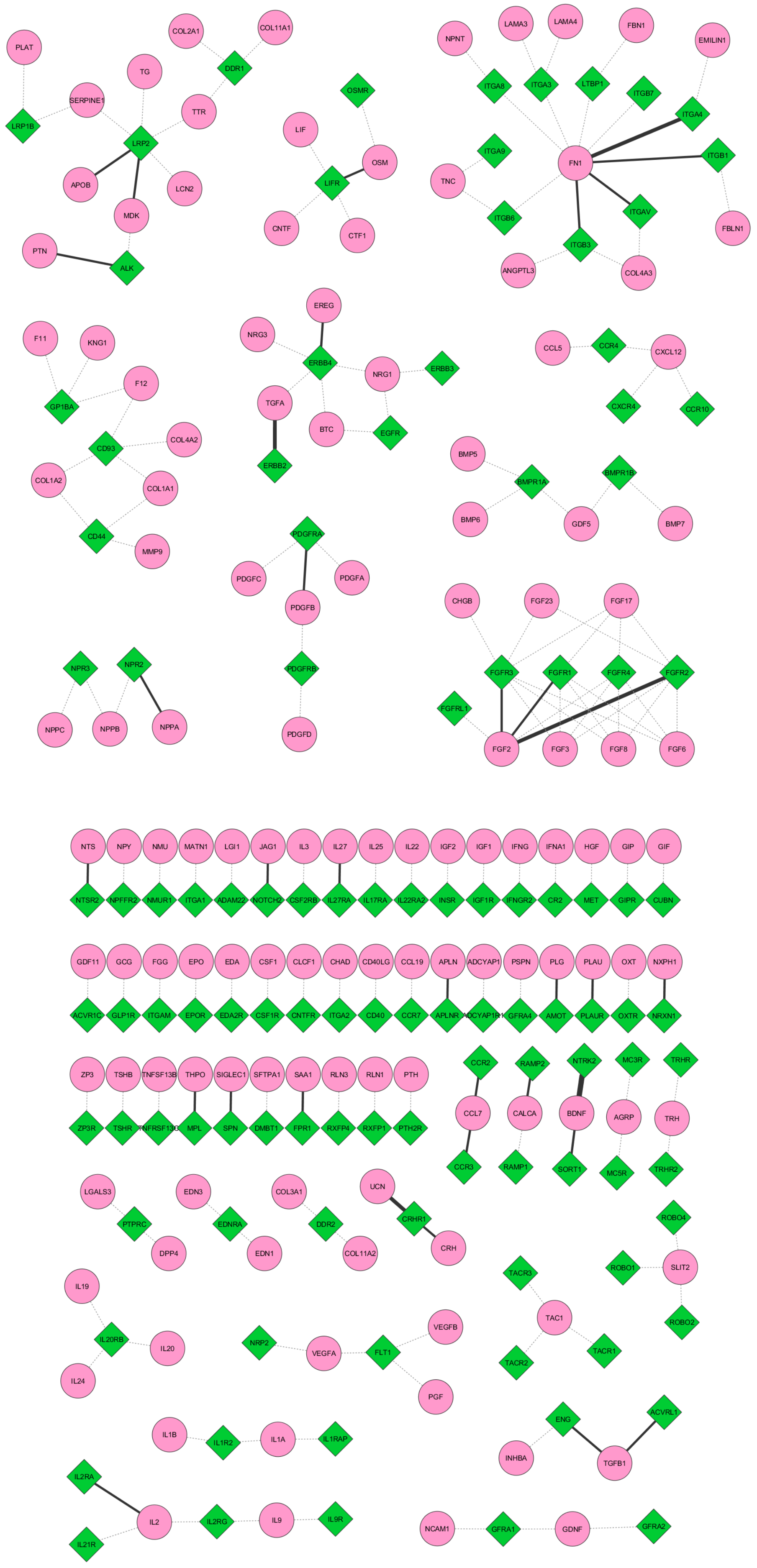

Supplementary Figure S3D

HFC: Sec-Up, Res-Up

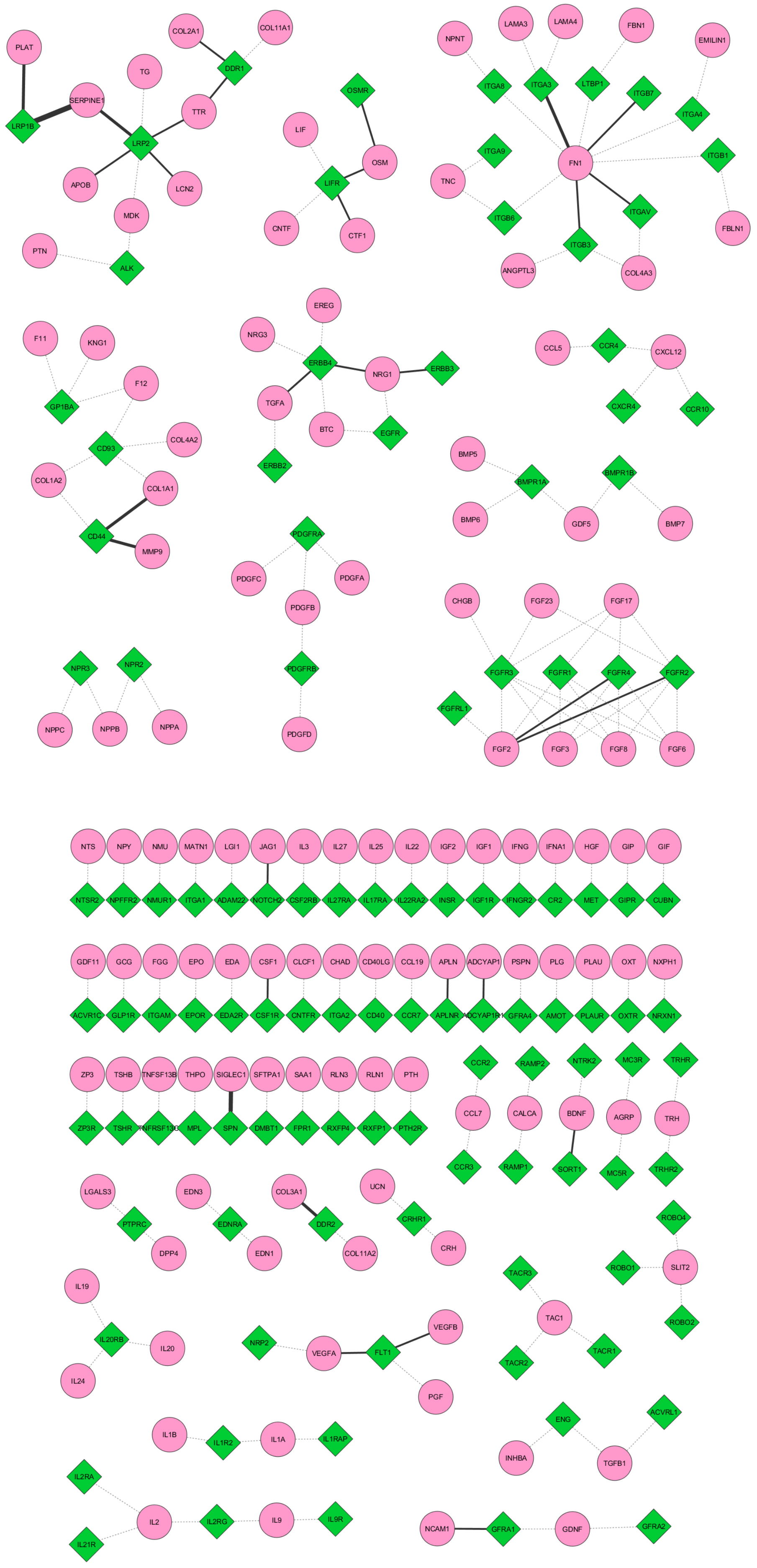

Supplementary Figure S3E

HFC+Kal1: Sec-Down, Res-Down

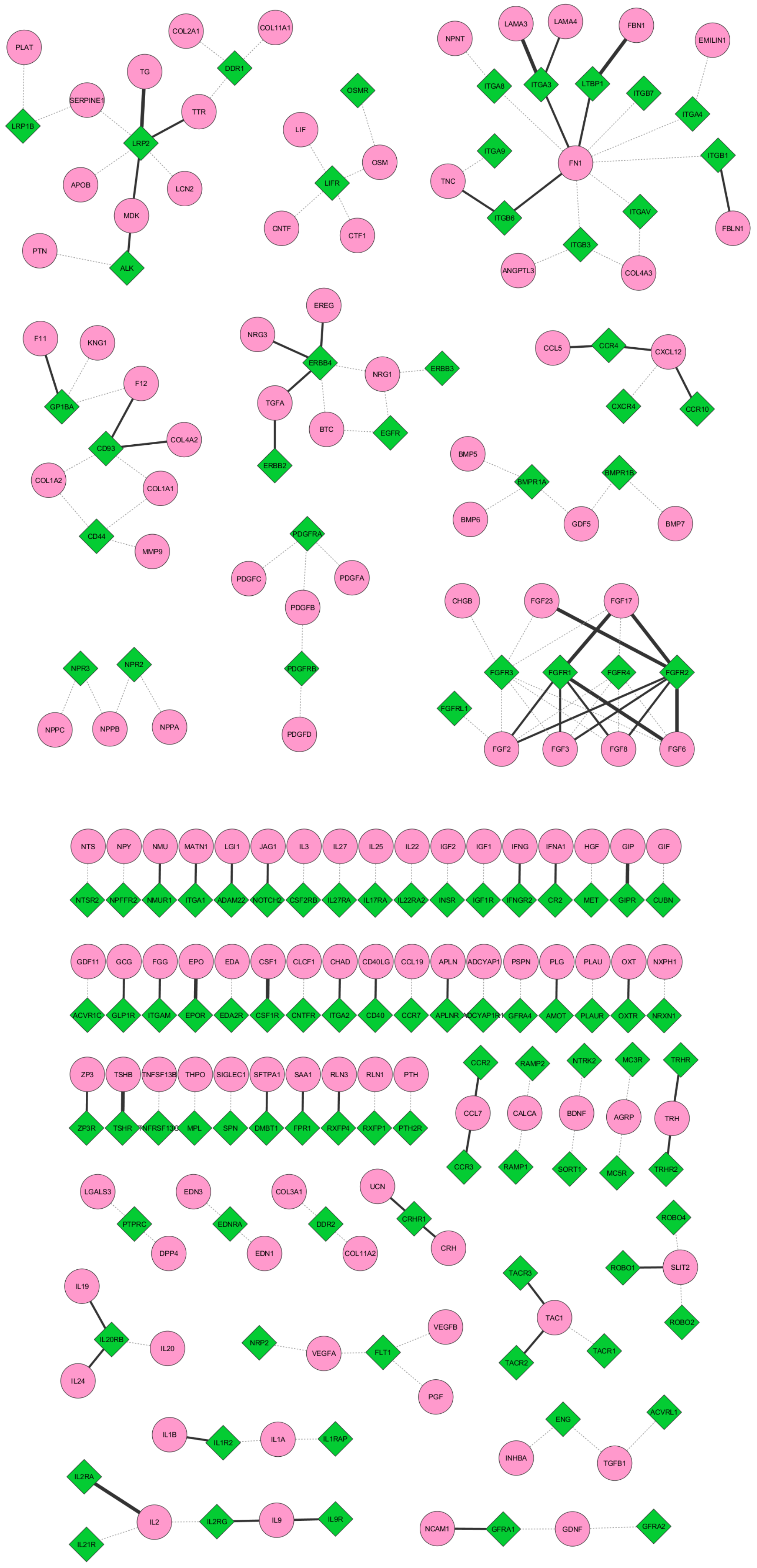

Supplementary Figure S3F

HFC+Kal1: Sec-Down, Res-Up

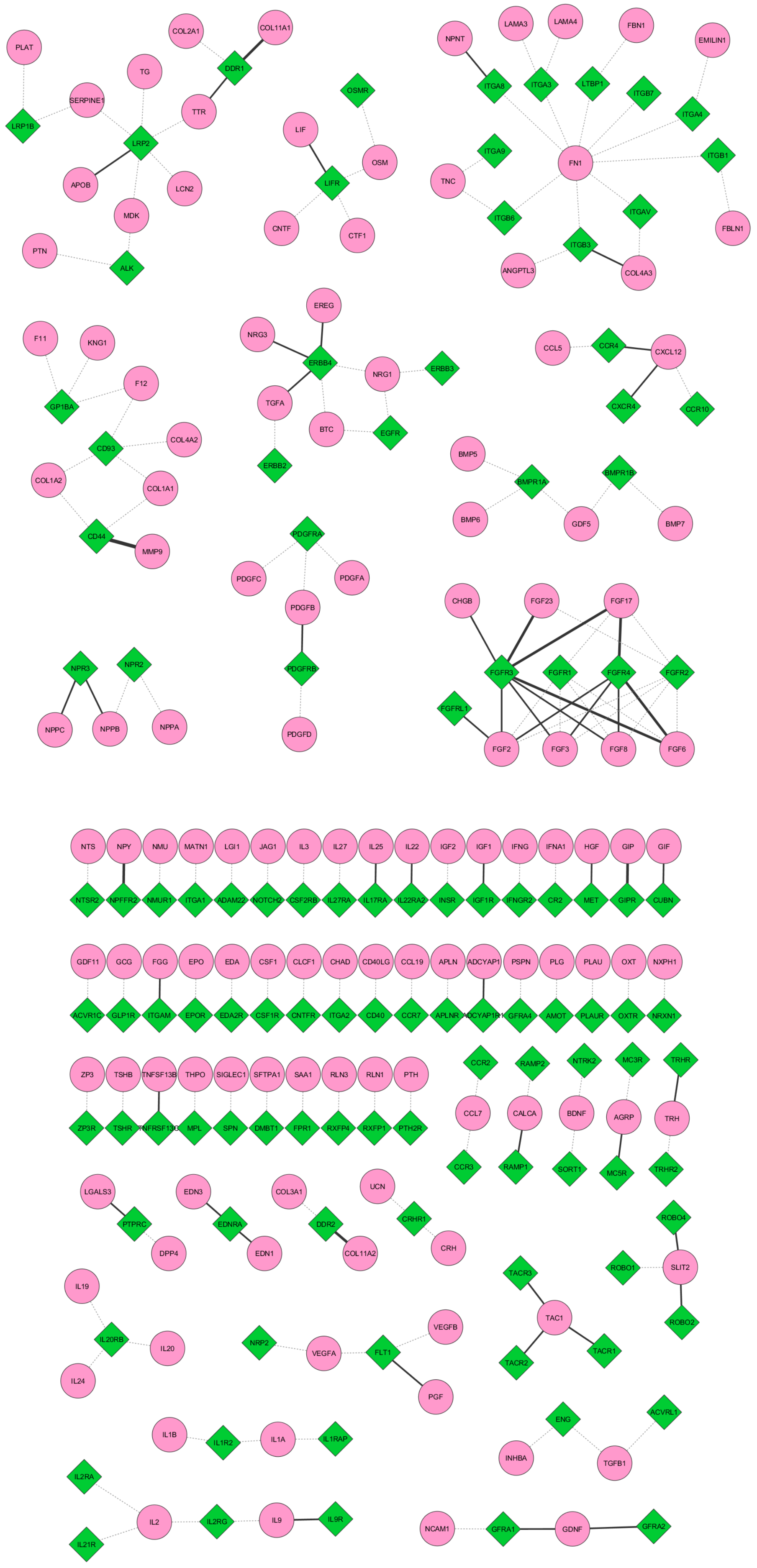

Supplementary Figure S3G

HFC+Kal1: Sec-Up, Res-Down

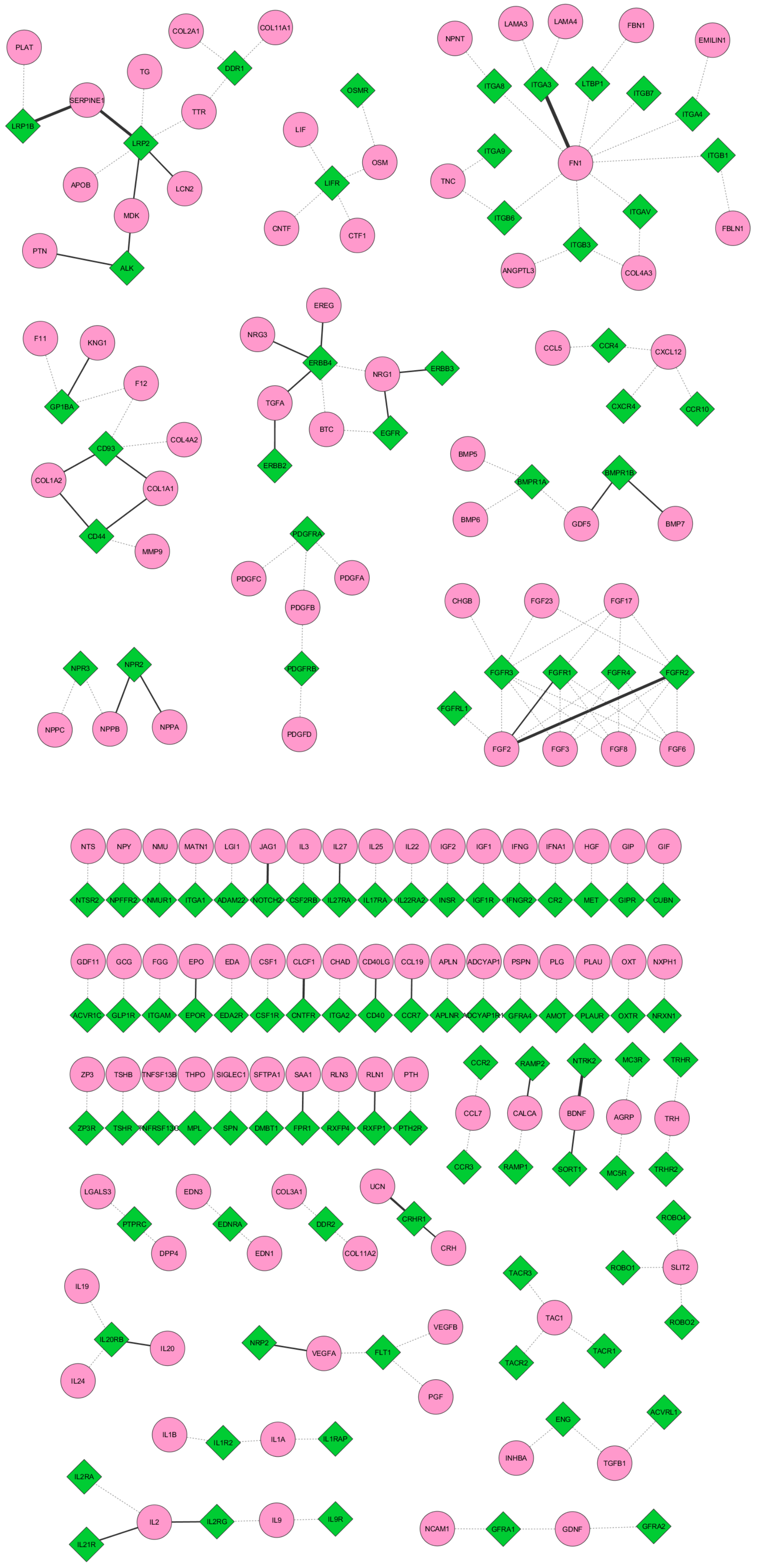

Supplementary Figure S3H

HFC+Kal1: Sec-Up, Res-Up

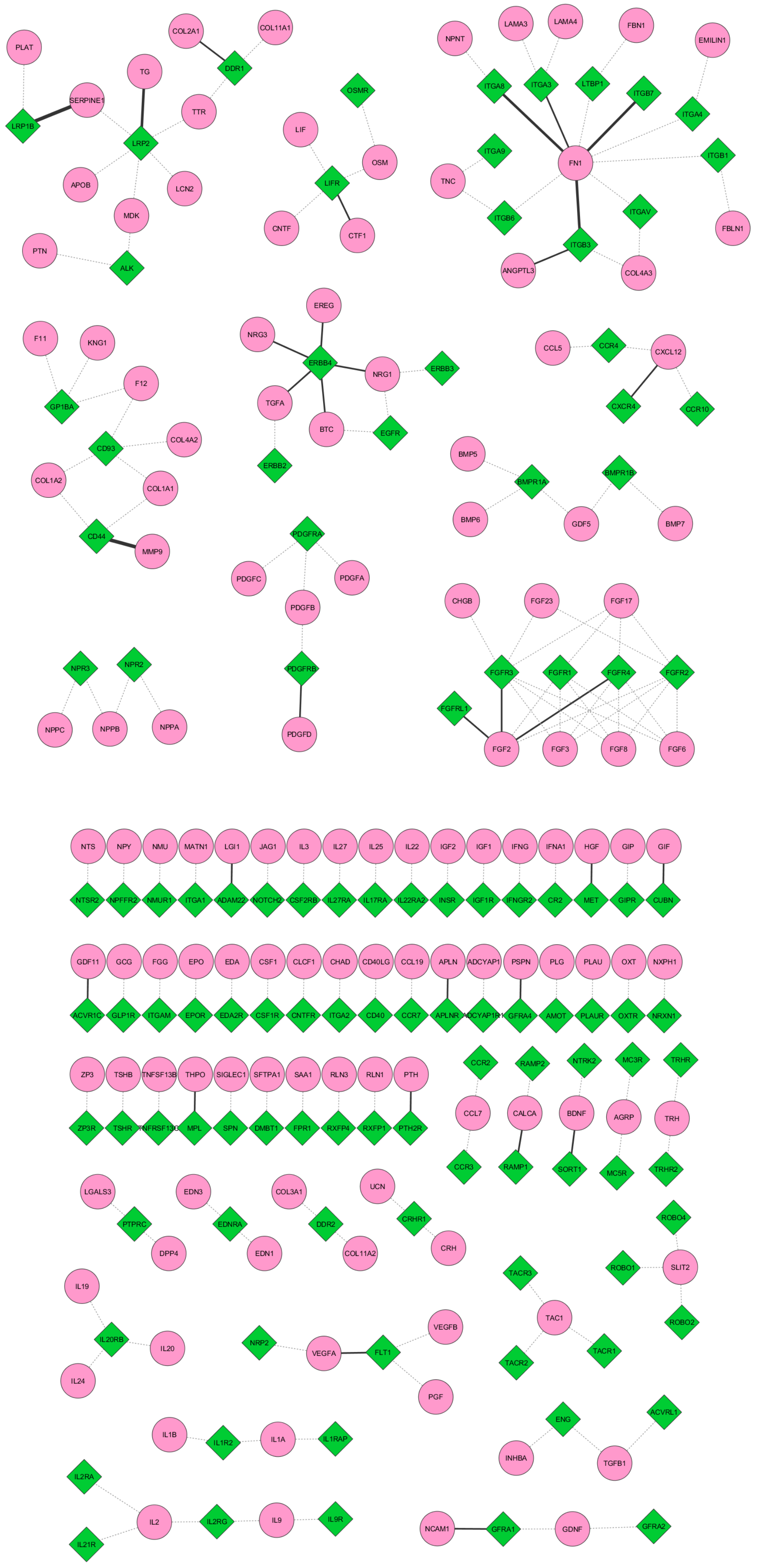

Supplementary Table S1

Tissues	Time points	Groups
Hippocampus	Days 1, 6, 14, 35, 56, 77, 98 and 119	LFC, HFHSD and HFHSD+Kal75
Splenic macrophages	Days 1, 6, 14, 35, 56, 77, 98 and 119	LFC, HFHSD and HFHSD+Kal75
Liver	Days 1, 6, 14, 35, 56, 77, 98 and 119	LFC, HFHSD and HFHSD+Kal75
Skeletal Muscle	Days 1, 6, 14, 35, 56, 77, 98 and 119	LFC, HFHSD and HFHSD+Kal75
Brown Adipose (BA)	Days 1, 6, 14, 35, 56, 77, 98 and 119	LFC, HFHSD and HFHSD+Kal75
White Adipose, Epididymis (EA)	Days 1, 6, 14, 35, 56, 77, 98 and 119	LFC, HFHSD and HFHSD+Kal75
White adipose, Subcutaneous (SA)	Days 1, 6, 14, 35, 56, 77, 98 and 119	LFC, HFHSD and HFHSD+Kal75
Infiltrating macrophages from SVC BA	Days 1, 6, 14, 35, 56, 77, 98 and 119	LFC, HFHSD and HFHSD+Kal75
Infiltrating macrophages from SVC EA	Days 1, 6, 14, 35, 56, 77, 98 and 119	LFC, HFHSD and HFHSD+Kal75
Infiltrating macrophages from SVC SA	Days 1, 6, 14, 35, 56, 77, 98 and 119	LFC, HFHSD and HFHSD+Kal75

Supplementary Table S2

Secretory Molecules	Receptors	Interactions	
		Secretory	Receptor
1100001G20Rik	abca1		
1110058L19Rik	abcc8	STX1A	ABCC9
1190007I07Rik	abcc9	AGT	ACE2
1300010F03Rik	abl1	GHRL	ACE2
1500015O10Rik	ace2	NTS	ACE2
1600002K03Rik	acvr1	BMP2	ACVR1
1600012H06Rik	acvr1b	BMP7	ACVR1
1700007K09Rik	acvr1c	ENG	ACVR1
1700029I15Rik	acvr2a	GDF5	ACVR1
1700034O15Rik	acvr2b	INHBA	ACVR1
1700040I03Rik	acvrl1	INHBB	ACVR1
1700066M21Rik	adam22	INHBC	ACVR1
1810030J14Rik	adcyap1r1	PLEK	ACVR1
2300002M23Rik	adipor1	CFC1	ACVR1B
2300005B03Rik	adipor2	GDF11	ACVR1B
2300009A05Rik	adora1	INHBA	ACVR1B
2310033E01Rik	adora2a	INHBB	ACVR1B
2310044H10Rik	adora2b	INHBC	ACVR1B
2310057J18Rik	adora3	TDGF1	ACVR1B
2410131K14Rik	adra1a	GDF11	ACVR1C
2610109H07Rik	adra1b	INHBB	ACVR1C
2610507B11Rik	adra1d	TDGF1	ACVR1C
3110057O12Rik	adra2a	BMP2	ACVR2A
4930486L24Rik	adra2b	BMP3	ACVR2A
4930503L19Rik	adra2c	BMP6	ACVR2A
4930572J05Rik	adrb1	BMP7	ACVR2A
4930578C19Rik	adrb2	ENG	ACVR2A
4931414P19Rik	adrb3	ERBB2IP	ACVR2A
4933434I20Rik	adrbk1	GDF5	ACVR2A
5730469M10Rik	af251705	GDF9	ACVR2A
6030468B19Rik	ager	INHA	ACVR2A
8030411F24Rik	agtr1	INHBA	ACVR2A
9030224M15Rik	agtr1a	INHBB	ACVR2A
9930032O22Rik	agtr1b	INHBC	ACVR2A
a	agtr2	TGFBR3	ACVR2A
A130022J15Rik	agtrap	BMP2	ACVR2B
A1bg	ahr	BMP3	ACVR2B
A2m	alk	BMP6	ACVR2B
A2ML1	amfr	BMP7	ACVR2B
A630095E13Rik	amhr2	ENG	ACVR2B
A6NC86	amot	ERBB2IP	ACVR2B
A6NDD2	anpep	GDF11	ACVR2B
A6NF02	antxr1	GDF5	ACVR2B
A6NG13	antxr2	INHBA	ACVR2B

A6NJS3	antxrl	INHBB	ACVR2B
A8MT79	anxa9	INHBC	ACVR2B
A8MTW9	aplnr	MSTN	ACVR2B
A8MUN3	apobr	TDGF1	ACVR2B
A8MXB1	ar	ENG	ACVRL1
A930038C07Rik	arntl	TGFB1	ACVRL1
AADACL2	asgr1	TGFB3	ACVRL1
Abhd15	asgr2	TGFBR3	ACVRL1
Abi3bp	atp5b	GRIN2B	ADAM22
Abo	atp6ap2	LGI1	ADAM22
ABP1	atrn	ADCYAP1	ADCYAP1R1
Abpa	au023871	ADIPOQ	ADIPOR2
Abpb	avpr1a	ADA	ADORA1
Abpg	avpr1b	GNAS	ADORA1
Abpz	avpr2	ACTN1	ADORA2A
Acan	ax2r	ACTN2	ADORA2A
Ace	axl	ACTN4	ADORA2A
ACE2	b430306n03rik	ADA	ADORA2A
Ache	b7z485	ADA	ADORA2B
Acp5	bai1	NTN1	ADORA2B
Acp6	bai2	BMP1	ADRA1A
Acpl2	bai3	ALB	ADRA1B
Acpp	bc006779	IGHG1	ADRA1B
Acrbp	bcam	IL1B	ADRB2
ACTN1	bcap29	GNAS	ADRB3
ACTN2	bcap31	EGFR	ADRBK1
ACTN4	bdkrb1	PEBP1	ADRBK1
Ada	bdkrb2	SNCA	ADRBK1
ADAM12	bgr	HMGB1	AGER
Adam17	bmp2	S100A12	AGER
Adam23	bmpr1a	S100B	AGER
ADAM28	bmpr1b	TLR9	AGER
Adam9	bmpr2	TTR	AGER
Adamdec1	bmx	AGT	AGTR1
Adamts1	brd8	EGFR	AGTR1
Adamts10	brs3	ACE	AGTR2
Adamts12	btla	AGT	AGTR2
Adamts13	btn1a1	ERBB2IP	AGTR2
Adamts14	bzrap1	ERBB3	AGTR2
Adamts15	c10orf54	TIMP3	AGTR2
Adamts16	c12orf76	HSP90AA1	AHR
ADAMTS17	c130060k24rik	IL10	AHR
Adamts18	c17orf57	IL21	AHR
Adamts19	c1orf85	IL8	AHR
Adamts2	c3ar1	HSPD1	ALK
Adamts20	c5ar1	INSL6	ALK
ADAMTS3	c6orf138	MDK	ALK

Adamts4	calcr	MEP1B	ALK
Adamts5	calcrl	PTN	ALK
ADAMTS6	casr	RAB35	ALK
Adamts7	ccbp2	GPI	AMFR
Adamts8	cckar	AMH	AMHR2
Adamts9	cckbr	PLG	AMOT
Adamtsl1	ccr1	FCGBP	ANPEP
Adamtsl2	ccr10	MEP1B	ANPEP
ADAMTSL3	ccr1l1	COL4A2	ANTXR2
Adamtsl4	ccr2	COL4A3	ANTXR2
ADAMTSL5	ccr3	COL4A4	ANTXR2
Adck1	ccr4	APLN	APLNR
Adcyap1	ccr5	APOL2	AR
Adipoq	ccr6	CALR	AR
Adm	ccr7	CASP1	AR
Adm2	ccr8	EGFR	AR
ADM5	ccr9	FLNA	AR
Adnp	ccrl1	GSN	AR
Adpgk	ccrl2	HMGB1	AR
Aebp1	cd14	HMGB2	AR
AER61	cd160	HSP90AA1	AR
Afm	cd163	IDE	AR
Afp	cd163l1	IL6ST	AR
AGER	cd180	KLK3	AR
Aggf1	cd1d	TGFB1	AR
AGO61	cd2	TGFB1I1	AR
Agr2	cd200r1	HSP90AA1	ARNTL
Agr3	cd200r1l	HSP90AA1	ASGR1
Agrn	cd200r2	TG	ASGR1
Agrp	cd200r3	F8	ASGR2
Agt	cd200r4	REN	ATP6AP2
Ahsg	cd209	ASIP	ATRN
AI413582	cd209a	AVP	AVPR1A
AI462493	cd209b	AVP	AVPR1B
AIMP1	cd209c	AVP	AVPR2
AKR1B1	cd209d	C1QTNF1	AVPR2
Akr1b3	cd209e	GNAS	AVPR2
Alad	cd22	GAS6	AXL
Alb	cd226	IL15RA	AXL
Aldoa	cd244	TLN1	BAI2
Alkbh7	cd247	LAMA5	BCAM
Alpl	cd27	APP	BCAP31
Ambn	cd274	CASP1	BCAP31
Ambp	cd28	MEP1A	BDKRB1
Amelx	cd300a	ACE	BDKRB2
AMELY	cd300c	BMPER	BMP2
Amh	cd300e	CHRDL2	BMP2

Amtn	cd300lb	COL2A1	BMP2
Amy1	cd300ld	ENG	BMP2
AMY1A	cd300lf	GREM2	BMP2
AMY2A	cd300lg	MGP	BMP2
Amy2a5	cd300lh	NOG	BMP2
AMY2B	cd302	SOSTDC1	BMP2
Ang	cd33	TGFB1	BMP2
Ang4	cd36	TGFB2	BMP2
Angpt1	cd38	BMP2	BMPR1A
Angpt2	cd3d	BMP4	BMPR1A
Angpt4	cd3e	BMP5	BMPR1A
Angptl1	cd3g	BMP6	BMPR1A
Angptl2	cd4	BMP7	BMPR1A
Angptl3	cd40	GDF5	BMPR1A
Angptl4	cd44	GDF6	BMPR1A
ANGPTL5	cd46	GDF9	BMPR1A
Angptl6	cd47	BMP15	BMPR1B
Angptl7	cd48	BMP2	BMPR1B
ANTXR2	cd5	BMP4	BMPR1B
Anxa1	cd55	BMP6	BMPR1B
Anxa2	cd5l	BMP7	BMPR1B
ANXA2P2	cd6	GDF5	BMPR1B
Anxa5	cd69	GDF6	BMPR1B
Aoah	cd7	GDF9	BMPR1B
Aoc3	cd72	ARSA	BMPR2
Apcs	cd74	BMP2	BMPR2
Apln	cd79a	BMP4	BMPR2
APLP1	cd79b	BMP6	BMPR2
Apoa1	cd80	BMP7	BMPR2
Apoa1bp	cd84	C4BPA	BMPR2
Apoa2	cd86	GDF5	BMPR2
Apoa4	cd8a	GDF6	BMPR2
Apoa5	cd8b	GDF9	BMPR2
Apob	cd93	SERPINA3K	BMPR2
APOBR	cd97	PLIN2	BTN1A1
Apoc1	cdhr1	XDH	BTN1A1
Apoc2	ceacam1	PLSCR1	C10ORF54
Apoc3	ceacam2	C3	C3AR1
Apoc4	celsr1	C4A	C3AR1
Apod	celsr2	C5	C5AR1
Apoe	celsr3	FLNA	CALCR
Apof	cfi	MBL2	CALCR
Apoh	chrm1	RAMP1	CALCR
APOL1	chrm2	THBS1	CALCR
APOL2	chrm3	ADM	CALCRL
APOL3	chrm4	CALCA	CALCRL
APOL4	chrm5	RAMP1	CALCRL

APOL5	chrna1	FLNA	CASR
APOL6	chrna10	CCL11	CCBP2
APOLD1	chrna2	CCL12	CCBP2
Apom	chrna3	CCL13	CCBP2
Apon	chrna4	CCL14	CCBP2
APOO	chrna5	CCL19	CCBP2
Apool	chrna6	CCL2	CCBP2
APP	chrna7	CCL21	CCBP2
Aqp1	chrna7-2	CCL25	CCBP2
Areg	chrna9	CCL27	CCBP2
Arg1	chrnb1	CCL28	CCBP2
Arsa	chrnb2	CCL3	CCBP2
ARSF	chrnb3	CCL4	CCBP2
Arsg	chrnb4	CCL5	CCBP2
Arsi	chrnd	CCL7	CCBP2
Arsj	chrne	CCL8	CCBP2
Arsk	chrng	CCR3	CCBP2
Art5	cldn3	CCK	CCKAR
Artn	cldn4	CCK	CCKBR
ASIP	clec12a	GAST	CCKBR
Aspn	clec12b	CCL1	CCR1
Atg4c	clec17a	CCL14	CCR1
ATRN	clec18a	CCL15	CCR1
AU021092	clec1a	CCL16	CCR1
Avp	clec1b	CCL2	CCR1
AY074887	clec2a	CCL23	CCR1
Azgp1	clec2d	CCL26	CCR1
AZU1	clec2g	CCL3	CCR1
B2m	clec2h	CCL3L1	CCR1
B4E171	clec2i	CCL4	CCR1
B4galt1	clec4a	CCL5	CCR1
BAGE	clec4a1	CCL7	CCR1
BAGE2	clec4a3	CCL8	CCR1
BAGE3	clec4a4	CCL19	CCR10
BAGE4	clec4b1	CCL2	CCR10
BAGE5	clec4b2	CCL21	CCR10
BC028528	clec4d	CCL25	CCR10
BC048546	clec4e	CCL27	CCR10
Bcan	clec4f	CCL28	CCR10
Bche	clec4m	CCL7	CCR10
Bdnf	clec5a	CXCL12	CCR10
Bglap	clec7a	CXCL13	CCR10
Bglap2	clec9a	CCL11	CCR2
Bglap-rs1	cmklr1	CCL13	CCR2
Bgn	cnr1	CCL16	CCR2
Bicd1	cnr2	CCL2	CCR2
Bmp1	cntfr	CCL7	CCR2

Bmp10	cntnap1	CCL8	CCR2
Bmp15	colec12	CCL11	CCR3
Bmp2	corin	CCL13	CCR3
Bmp3	cr1	CCL14	CCR3
Bmp4	cr1l	CCL15	CCR3
Bmp5	cr2	CCL2	CCR3
Bmp6	crcp	CCL24	CCR3
Bmp7	crhr1	CCL26	CCR3
Bmp8a	crhr1l	CCL28	CCR3
Bmp8b	crhr2	CCL3	CCR3
Bmper	crim1	CCL3L1	CCR3
Bola1	crlf1	CCL4	CCR3
Bola3	crlf2	CCL5	CCR3
Bpi	crlf3	CCL7	CCR3
BPIFA1	cry1	CCL8	CCR3
BPIFA2	cry2	CXCL10	CCR3
Bpifa2e	csf1r	CXCL11	CCR3
Bpifa3	csf2ra	CXCL9	CCR3
Bpifa5	csf2rb	CCL17	CCR4
Bpifb1	csf2rb2	CCL22	CCR4
Bpifb2	csf3r	CCL3	CCR4
Bpifb3	cubn	CCL5	CCR4
Bpifb4	cul5	CXCL12	CCR4
Bpifb6	cx3cr1	AFP	CCR5
Bpifc	cxadr	CCL11	CCR5
BRPF3	cxcl16	CCL13	CCR5
Bsph1	cxcr1	CCL14	CCR5
Btbd17	cxcr2	CCL16	CCR5
Btc	cxcr3	CCL2	CCR5
Btd	cxcr4	CCL3	CCR5
BTN1A1	cxcr5	CCL3L1	CCR5
C10orf25	cxcr6	CCL4	CCR5
C10orf31	cxcr7	CCL5	CCR5
C10orf58	cysltr1	CCL7	CCR5
C10orf99	cysltr2	CCL8	CCR5
C11orf44	cyth3	CCR3	CCR5
C11orf45	dag1	ORM1	CCR5
C11orf83	darc	CCL20	CCR6
C11orf94	dbi	DEFB1	CCR6
C12orf28	dcc	DEFB103A	CCR6
C12orf39	ddr1	CCL19	CCR7
C12orf49	ddr2	CCL21	CCR7
C12orf73	dear1	CCL1	CCR8
C14orf144	derl1	CCL16	CCR8
C14orf93	dgcr2	CCL17	CCR8
C15orf61	disp1	CCL4	CCR8
C16orf89	dmbt1	CCL25	CCR9

C17orf67	dnajc13	CCL11	CCRL1
C17orf69	dner	CCL13	CCRL1
C17orf77	dpp4	CCL19	CCRL1
C17orf99	drd1	CCL2	CCRL1
C18orf54	drd1a	CCL21	CCRL1
C19orf10	drd2	CCL25	CCRL1
C19orf24	drd3	CCL5	CCRL1
C19orf63	drd4	CCL7	CCRL1
C19orf80	drd5	CCL8	CCRL1
C1orf134	ebi3	CXCL13	CCRL1
C1orf187	ebp	CCL2	CCRL2
C1orf191	eda2r	CD55	CD14
C1orf54	edar	LBP	CD14
C1orf56	edaradd	LGALS3BP	CD14
C1qa	ednra	LTF	CD14
C1qb	ednrb	TLR9	CD14
C1qbp	efemp1	HP	CD163
C1qc	efemp2	LY86	CD180
C1ql1	efna3	B2M	CD1D
C1ql2	efna4	CALR	CD1D
C1ql3	efnb3	P4HB	CD1D
C1ql4	egfr	CD59	CD2
C1qtnf1	eif2d	LGALS1	CD2
C1qtnf2	elovl4	CLEC4M	CD209
C1qtnf3	eltd1	HP	CD22
C1qtnf4	emr1	IGHM	CD22
C1qtnf5	emr2	ST6GAL1	CD22
C1qtnf6	emr3	PVR	CD226
C1qtnf7	emr4	CD2	CD247
C1QTNF8	emr4p	FCGR3A	CD247
C1qtnf9	endou	TFRC	CD247
C1QTNF9B	eng	CD70	CD27
C1R	enpp1	IL12A	CD28
C1rl	enpp2	LEP	CD33
C1S	enpp3	MUC2	CD33
C2	ep3-i	COL1A1	CD36
C22orf46	epha1	COL1A2	CD36
C2CD2	epha10	COL2A1	CD36
C2orf40	epha2	COL4A1	CD36
C2orf66	epha3	COL4A2	CD36
C2orf69	epha4	COL6A1	CD36
C3	epha5	FN1	CD36
C330005M16Rik	epha6	ITGA6	CD36
C3orf58	epha7	ITGB1	CD36
C3P1	epha8	LDLR	CD36
C4A	ephb1	THBS1	CD36
C4b	ephb2	VLDLR	CD36

C4B-1	ephb3	FCGR3A	CD38
C4bp	ephb4	PECAM1	CD38
C4BPA	ephb6	CD8A	CD3D
C4BPB	epor	CD8B	CD3D
C4orf26	eps15	IGKC	CD3E
C4orf29	eps15l1	LGALS1	CD3E
C4orf40	eps8	ANXA1	CD4
C4orf48	eps8l1	ANXA2	CD4
C5	erbb2	CD2	CD4
C5orf38	erbb3	CTSD	CD4
C5orf46	erbb4	CXCL12	CD4
C5orf55	errfi1	DPP4	CD4
C5orf64	esna1	FCGR3A	CD4
C6	esr1	HSP90AA1	CD4
C6orf1	esr2	HSPA8	CD4
C6orf120	esrra	HSPD1	CD4
C6orf126	esrrb	IL16	CD4
C6orf127	esrrg	LGALS1	CD4
C6orf15	evi2a	PIP	CD4
C6orf186	f2	TFRC	CD4
C6orf27	f2r	YBX1	CD4
C6orf57	f2rl1	CALR	CD40
C6orf58	f2rl2	CD40LG	CD40
C7	f2rl3	HSPA8	CD40
C7orf34	f5h4k3	IL4R	CD40
C7orf69	f630003a18rik	TNFSF13B	CD40
C7orf73	f8vs73	TXN	CD40
C85492	f8wb81	COL14A1	CD44
C8a	fam89b	COL1A1	CD44
C8b	fas	COL1A2	CD44
C8g	fcamr	DMP1	CD44
C8orf55	fcar	EGFR	CD44
C9	fcer1a	FGF2	CD44
C9orf47	fcer1g	FN1	CD44
C9orf8	fcer2	HBEGF	CD44
CA11	fcer2a	IGFBP3	CD44
CA2	fcgr1	LGALS9	CD44
CA6	fcgr1a	MMP1	CD44
CABP4	fcgr1b	MMP7	CD44
Calca	fcgr1c	MMP9	CD44
CALCB	fcgr2a	SELE	CD44
CALM3	fcgr2b	SPN	CD44
Calr	fcgr2c	SPP1	CD44
Calu	fcgr3	SRGN	CD44
Camp	fcgr3a	VCAN	CD44
CAP1	fcgr3b	C3	CD46
CAPZA1	fcgrt	C4B	CD46

CAPZA2	fcrl1	ITGB1	CD46
Car11	fcrl2	ALDOA	CD47
Car15	fcrl3	FAS	CD47
Car2	fcrl4	FCER2	CD47
Car6	fcrl5	ITGB1	CD47
Cartpt	fcrla	THBS1	CD47
Casp1	fcrls	CD2	CD48
Cbln1	ffar1	CD2	CD5
Cbln3	ffar2	CD27	CD5
Cbln4	ffar3	CD14	CD55
Ccbe1	fgfr1	CD97	CD55
Ccdc126	fgfr2	GPLD1	CD55
Ccdc134	fgfr3	CD40LG	CD5L
Ccdc3	fgfr4	CTSL1	CD7
Ccdc70	fgfrl1	LGALS1	CD7
Ccdc80	fkbp1a	SECTM1	CD7
Cck	fkbp3	CTSL1	CD74
Ccl1	flt1	MIF	CD74
Ccl11	flt3	AMBP	CD79A
Ccl12	flt4	FCAR	CD79A
CCL13	flvcr1	FN1	CD79A
CCL14	folr1	IGHM	CD79A
CCL15	folr2	IGJ	CD79A
CCL16	folr3	PIGR	CD79A
Ccl17	folr4	B2M	CD8A
CCL18	fpr1	CD8B	CD8A
CCL19	fpr2	LGALS1	CD8A
Ccl2	fpr3	CD8A	CD8B
Ccl20	fpr-rs3	ST3GAL4	CD8B
CCL21	fpr-rs4	C1QA	CD93
Ccl22	fpr-rs6	COL1A1	CD93
CCL23	frs3	COL1A2	CD93
Ccl24	frzb	COL4A1	CD93
Ccl25	fshr	COL4A2	CD93
CCL26	fzd1	COL4A3	CD93
CCL27	fzd10	COL4A4	CD93
Ccl28	fzd2	COL4A5	CD93
Ccl3	fzd3	COL4A6	CD93
CCL3L1	fzd4	F12	CD93
CCL3L3	fzd5	KNG1	CD93
Ccl4	fzd6	MBL2	CD93
CCL4L1	fzd7	SFTPA2	CD93
CCL4L2	fzd8	CD55	CD97
Ccl5	fzd9	PSAP	CELSR1
Ccl6	g6b	CSF1	CELSR3
Ccl7	gab1	UACA	CELSR3
Ccl8	gab2	C3	CFI

Ccl9	gab3	CFH	CFI
CCR3	gabarap	CRELD2	CHRNA4
Cd109	gabarapl1	APP	CHRNA7
Cd14	gabbr1	CRELD2	CHRNB2
CD163	gabbr2	SHBG	CLDN4
CD163L1	gabra1	CD209	CLEC4M
CD164	gabra2	CEACAM1	CLEC4M
CD2	gabra3	MET	CNR1
CD200R1	gabra4	CLCF1	CNTFR
CD209	gabra5	CNTF	CNTFR
CD248	gabra6	CRLF1	CNTFR
CD27	gabrb1	IL6ST	CNTFR
Cd34	gabrb2	LIFR	CNTFR
CD40	gabrb3	PLSCR1	CNTFR
Cd40lg	gabrd	C1QA	CR1
Cd46	gabre	C3	CR1
CD47	gabrg1	C4A	CR1
CD55	gabrg2	C4B	CR1
CD59	gabrg3	CD55	CR1
Cd59b	gabrp	C3	CR2
CD5L	gabrq	FCER2	CR2
Cd70	gabrr1	IFNA1	CR2
CD8A	gabrr2	RAMP1	CRCP
CD8B	gabrr3	CRH	CRHR1
CD97	galr1	GNAS	CRHR1
CDA	galr2	UCN	CRHR1
CDCP1	galr3	UCN3	CRHR1
Cdcp2	gcgr	CRH	CRHR2
Cdh13	gfra1	UCN	CRHR2
Cdnf	gfra2	UCN2	CRHR2
Cdsn	gfra3	UCN3	CRHR2
CEACAM1	gfra4	CLCF1	CRLF1
Ceacam10	gfral	IL7R	CRLF2
CEACAM8	ghr	TSLP	CRLF2
CECR1	ghrhr	CSF1	CSF1R
CECR9	ghsr	HSPA8	CSF1R
Cel	gipr	CSF2	CSF2RA
CELA1	git1	KIT	CSF2RA
Cela2a	git2	CSF2	CSF2RB
CELA2B	glp1r	CSF2RA	CSF2RB
CELA3B	glp2r	EPOR	CSF2RB
Cer1	glra1	IL3	CSF2RB
Ces1c	glra2	IL5	CSF2RB
CES1P1	glra3	IL5RA	CSF2RB
Ces4a	glra4	KIT	CSF2RB
Ces5a	glrb	CSF2	CSF3R
CETP	gm106	CSF3	CSF3R

Cfb	gm13318	ELANE	CSF3R
Cfc1	gm14496	PLA2G1B	CSF3R
CFC1B	gm156	ALB	CUBN
Cfd	gm5127	GC	CUBN
Cfdp1	gm725	GIF	CUBN
Cfh	gm9268	LGALS3	CUBN
CFHR1	gnat2	LRP2	CUBN
CFHR2	gnb2l1	SCGB1A1	CUBN
CFHR3	gnrhr	TF	CUBN
CFHR4	gnrhr2	KARS	CUL5
CFHR5	gosr1	CX3CL1	CX3CR1
CFI	gosr2	CXCL1	CXCR1
Cfp	gp1ba	CXCL2	CXCR1
Cga	gp1bb	CXCL3	CXCR1
CGB1	gp6	CXCL5	CXCR1
CGB2	gpa33	CXCL6	CXCR1
CGB7	gpbar1	IL8	CXCR1
Cgref1	gper	PPBP	CXCR1
Chad	gpld1	CXCL1	CXCR2
CHADL	gpr1	CXCL2	CXCR2
Chga	gpr101	CXCL3	CXCR2
Chgb	gpr107	CXCL5	CXCR2
Chi3l1	gpr110	CXCL6	CXCR2
CHI3L2	gpr111	IL8	CXCR2
Chi3l3	gpr112	PPBP	CXCR2
Chi3l4	gpr113	CCL11	CXCR3
Chia	gpr114	CCL13	CXCR3
Chid1	gpr115	CCL20	CXCR3
Chit1	gpr116	CCL21	CXCR3
Chl1	gpr119	CCL5	CXCR3
Chrd	gpr12	CCL7	CXCR3
Chrdl1	gpr123	CXCL10	CXCR3
Chrdl2	gpr124	CXCL11	CXCR3
CHST9	gpr125	CXCL13	CXCR3
CHSY1	gpr126	CXCL9	CXCR3
Cilp	gpr128	PF4	CXCR3
CILP2	gpr132	CTSG	CXCR4
Cklf	gpr133	CXCL12	CXCR4
CLCA1	gpr135	DPP4	CXCR4
CLCA2	gpr137	ELANE	CXCR4
Clca3	gpr137b	HSPA8	CXCR4
CLCA3P	gpr139	SDC4	CXCR4
CLCA4	gpr141	CCL16	CXCR5
Clcf1	gpr142	CCL3	CXCR5
Clec11a	gpr143	CCL4	CXCR5
CLEC18A	gpr144	CCL5	CXCR5
CLEC18B	gpr146	CXCL13	CXCR5

CLEC18C	gpr148	CXCL16	CXCR6
CLEC19A	gpr149	AGR3	DAG1
Clec3a	gpr15	AGRN	DAG1
Clec3b	gpr150	HSPG2	DAG1
CLEC4M	gpr151	LAMA1	DAG1
Clps	gpr152	LAMA2	DAG1
Clstn1	gpr153	LAMA5	DAG1
Clu	gpr155	CCL17	DARC
CLUL1	gpr156	CCL2	DARC
Cma1	gpr157	CCL5	DARC
Cml1	gpr158	CCL7	DARC
Cml2	gpr160	CCL8	DARC
Cml3	gpr161	CXCL1	DARC
Cml5	gpr162	CXCL5	DARC
CMTM1	gpr165	IL8	DARC
CMTM2	gpr17	PF4	DARC
Cmtm2a	gpr171	ALB	DCC
Cmtm2b	gpr172a	DSCAM	DCC
Cmtm3	gpr172b	NTN1	DCC
Cmtm4	gpr173	COL11A1	DDR1
Cmtm5	gpr174	COL2A1	DDR1
Cmtm6	gpr176	COL3A1	DDR1
Cmtm7	gpr177	COL5A2	DDR1
Cmtm8	gpr179	TTR	DDR1
CNDP1	gpr18	COL11A2	DDR2
Cnp	gpr182	COL1A1	DDR2
Cnpy4	gpr183	COL3A1	DDR2
Cntf	gpr19	ALB	DERL1
Cntn4	gpr20	MFI2	DERL1
CNTNAP3	gpr21	SELS	DERL1
Coch	gpr22	SFTPA1	DMBT1
Col10a1	gpr25	SFTPD	DMBT1
Col11a1	gpr26	ADA	DPP4
Col11a2	gpr27	CCL11	DPP4
Col12a1	gpr3	CCL22	DPP4
Col13a1	gpr30	CCL3L1	DPP4
Col14a1	gpr31	CCL5	DPP4
Col15a1	gpr32	CXCL10	DPP4
Col16a1	gpr33	CXCL11	DPP4
Col17a1	gpr34	CXCL12	DPP4
Col18a1	gpr35	CXCL2	DPP4
Col19a1	gpr37	CXCL9	DPP4
Col1a1	gpr37l1	GRP	DPP4
Col1a2	gpr39	ADA	DRD1
Col20a1	gpr4	FLNA	DRD1
COL21A1	gpr42	FLNA	DRD2
COL22A1	gpr44	FLNA	DRD3

Col23a1	gpr45	IL12A	EBI3
Col24a1	gpr50	IL27	EBI3
Col25a1	gpr52	EDA	EDA2R
Col27a1	gpr55	EDA	EDAR
Col28a1	gpr56	EDN1	EDNRA
Col2a1	gpr6	EDN3	EDNRA
Col3a1	gpr61	EDN1	EDNRB
Col4a1	gpr62	EDN3	EDNRB
Col4a2	gpr63	TIMP3	EFEMP1
Col4a3	gpr64	AQP1	EFEMP2
Col4a4	gpr65	COL8A1	EFEMP2
Col4a5	gpr68	IL16	EFEMP2
Col4a6	gpr75	PLSCR1	EFEMP2
Col5a1	gpr77	PRSS23	EFNA3
Col5a2	gpr78	AMH	EGFR
Col5a3	gpr81	ANXA1	EGFR
Col6a1	gpr82	AREG	EGFR
Col6a2	gpr83	BTC	EGFR
Col6a3	gpr84	CASP1	EGFR
Col6a4	gpr85	CD59	EGFR
Col6a5	gpr87	CDCP1	EGFR
Col6a6	gpr88	CEACAM1	EGFR
Col7a1	gpr97	CMTM8	EGFR
Col8a1	gpr98	DCN	EGFR
Col8a2	gprc5a	EGF	EGFR
Col9a1	gprc5b	ERBB3	EGFR
Col9a2	gprc5c	EREG	EGFR
Col9a3	gprc5d	FAS	EGFR
Colec10	gprc6a	HBEGF	EGFR
Colec11	grb10	HSP90AA1	EGFR
COLEC12	grb14	HSPA5	EGFR
Colq	grb2	HSPA8	EGFR
Comp	grb7	HSPD1	EGFR
Copa	gria1	ICAM1	EGFR
Cort	gria2	LRSAM1	EGFR
Cp	gria3	MET	EGFR
Cpa1	gria4	MUC1	EGFR
Cpa2	grid1	NRG1	EGFR
Cpa4	grid2	PLSCR1	EGFR
Cpa5	grik1	S100A7	EGFR
Cpa6	grik2	S100A9	EGFR
CPAMD8	grik3	SFN	EGFR
CPB1	grik4	TGFA	EGFR
Cpb2	grik5	TLN1	EGFR
Cpe	grin1	TNC	EGFR
Cpn1	grin2a	TUBA4A	EGFR
Cpn2	grin2b	TXN	EGFR

CPO	grin2c	BMP2	ENG
Cpxm1	grin2d	BMP7	ENG
Cpxm2	grin3a	INHBA	ENG
Cpz	grin3b	TGFB1	ENG
CR1L	grk1	TGFB2	ENG
CRB1	grk5	TGFB3	ENG
CRB2	grk6	TGFBR3	ENG
Creg1	grlf1	EFNA1	EPHA1
Creg2	grm1	EFNA1	EPHA2
Creld2	grm2	EFNA4	EPHA2
Crh	grm3	EGFR	EPHA2
Crhbp	grm4	EFNA1	EPHA3
Crim1	grm5	EFNA1	EPHA4
CRIP2	grm6	EFNA4	EPHA4
Crisp1	grm7	FGFR1	EPHA4
Crisp2	grm8	FGFR2	EPHA4
Crisp3	grpr	FGFR4	EPHA4
Crispld1	gucy1a3	EFNA1	EPHA5
Crispld2	gucy1b3	EFNA4	EPHA5
CRLF1	gucy2c	EFNA1	EPHA6
CRLF2	gucy2d	EFNA1	EPHA7
Crp	gucy2f	EFNA4	EPHA7
Crtac1	gucy2g	EFNA1	EPHA8
Crtap	gypa	EFNA4	EPHA8
Csf1	havcr1	EFNA1	EPHB1
Csf2	hbegf	EPHB6	EPHB1
CSF2RA	hcar1	AQP1	EPHB2
Csf3	hcar2	GRIN2B	EPHB2
CSF3R	hcar3	EGFR	EPHB4
CSH1	hcg_1774561	EPO	EPOR
CSH2	hcg_2044188	KIT	EPOR
CSHL1	hcrtr1	KITLG	EPOR
Csn1s1	hcrtr2	KLK3	EPOR
Csn1s2a	hctr-5	ACPP	ERBB2
Csn1s2b	hctr-6	BTC	ERBB2
Csn2	heyl	EGF	ERBB2
CSN3	hhipl1	EGFR	ERBB2
CST1	hla-a	ERBB2IP	ERBB2
Cst10	hla-b	ERBB3	ERBB2
Cst11	hla-c	HSP90AA1	ERBB2
Cst12	hla-doa	IL6ST	ERBB2
Cst13	hla-dob	MUC1	ERBB2
CST2	hla-dpa1	MUC4	ERBB2
Cst3	hla-dqa1	NRG1	ERBB2
CST4	hla-dqa2	SERPINA3	ERBB2
CST5	hla-dqb1	TGFA	ERBB2
CST6	hla-dqb2	TLN1	ERBB2

Cst7	hla-dra	EGF	ERBB3
Cst8	hla-drb1	EGFR	ERBB3
Cst9	hla-drb3	FGFR1	ERBB3
CST9L	hla-e	IL6ST	ERBB3
CST9L2	hla-f	MUC1	ERBB3
CSTL1	hla-g	NRG1	ERBB3
Ctf1	hla-h	NRG2	ERBB3
Ctf2	hnf4a	ADAM17	ERBB4
Ctgf	hnf4g	BTC	ERBB4
CTHRC1	hpgd	CTGF	ERBB4
Ctla2a	hpn	EGFR	ERBB4
Ctrb1	hrankl3	ERBB3	ERBB4
CTRB2	hrh1	EREG	ERBB4
CTRL	hrh2	HBEGF	ERBB4
Ctsb	hrh3	MUC1	ERBB4
Ctsd	hrh4	NRG1	ERBB4
Ctsg	hspa1a	NRG2	ERBB4
Ctsk	htr1a	NRG3	ERBB4
CTSL1	htr1b	NRG4	ERBB4
CTSS	htr1d	TGFA	ERBB4
Ctsz	htr1e	BDNF	ESR1
Cx3cl1	htr1f	EGFR	ESR1
CXADR	htr2a	HSP90AA1	ESR1
Cxcl1	htr2b	OXT	ESR2
Cxcl10	htr2c	CTSG	F2R
Cxcl11	htr3a	ELANE	F2R
Cxcl12	htr3b	F2	F2R
Cxcl13	htr3c	MMP1	F2R
Cxcl14	htr3d	PLG	F2R
Cxcl15	htr3e	PRTN3	F2R
CXCL16	htr4	CTSG	F2RL1
Cxcl17	htr5a	ELANE	F2RL1
Cxcl2	htr5b	F2	F2RL1
Cxcl3	htr6	PLG	F2RL1
Cxcl5	htr7	PRTN3	F2RL1
CXCL6	hyal2	ST14	F2RL1
Cxcl9	icam1	CTSG	F2RL2
CXorf36	ifnar1	F2	F2RL2
Cyb5d2	ifnar2	F2RL3	F2RL2
Cyr61	ifngr1	JAM3	F2RL2
CYTL1	ifngr2	F2	F2RL3
D17H6S56E-3	igf1r	F2RL2	F2RL3
D17Wsu104e	igf2r	CD47	FAS
D730048I06Rik	igflr1	CEACAM1	FAS
Dag1	igsf1	EGFR	FAS
Dand5	igsf10	FASLG	FAS
DBH	igsf11	LGALS1	FAS

Dbi	igsf6	LGALS3	FAS
DCD	il10ra	MET	FAS
Dcn	il10rb	TNFRSF1A	FAS
Dcpp1	il11ra	TNFSF13	FAS
DDR1	il11ra1	APCS	FCAR
DEAF1	il12b	CRP	FCAR
Defa1	il12rb1	PILRA	FCAR
Defa2	il12rb2	ITIH2	FCER1A
Defa21	il13ra1	FCGR3A	FCER1G
Defa22	il13ra2	CD47	FCER2
Defa24	il15ra	APCS	FCGR1A
Defa25	il17ra	CRP	FCGR1A
Defa26	il17rb	FCAR	FCGR1A
Defa3	il17rc	FCGR3A	FCGR1A
Defa4	il17rd	HRG	FCGR1A
DEFA5	il17re	CRP	FCGR2A
Defa6	il18r1	LGALS3	FCGR2A
Defa-rs10	il18rap	APCS	FCGR2B
Defa-rs12	il1r1	CRP	FCGR2B
Defa-rs2	il1r2	IGHG1	FCGR2B
Defb1	il1rap	CRP	FCGR2C
Defb10	il1rapl1	APCS	FCGR3A
DEFB103A	il1rapl2	IGHG1	FCGR3A
DEFB104B	il1rl1	APCS	FCGR3B
DEFB105A	il1rl2	IGHG1	FCGR3B
DEFB106B	il1rn	ALB	FCGRT
DEFB107B	il20ra	B2M	FCGRT
DEFB108B	il20rb	CA6	FCGRT
DEFB108P1	il21r	ERBB3	FGFR1
DEFB109	il22ra1	FGF1	FGFR1
Defb11	il22ra2	FGF17	FGFR1
DEFB110	il23r	FGF18	FGFR1
DEFB112	il27ra	FGF2	FGFR1
DEFB113	il28ra	FGF3	FGFR1
DEFB114	il2ra	FGF4	FGFR1
DEFB115	il2rb	FGF5	FGFR1
DEFB116	il2rg	FGF6	FGFR1
DEFB118	il31ra	FGF7	FGFR1
DEFB119	il3ra	FGF8	FGFR1
Defb12	il4r	FGF9	FGFR1
DEFB121	il4ra	IGHG1	FGFR1
DEFB123	il5ra	MMP2	FGFR1
DEFB124	il6r	NCAM1	FGFR1
DEFB125	il6ra	NRP1	FGFR1
DEFB126	il6st	RTN3	FGFR1
DEFB127	il7r	FGF1	FGFR2
DEFB128	il8rb	FGF10	FGFR2

DEFB129	il9r	FGF17	FGFR2
Defb13	ildr1	FGF18	FGFR2
DEFB130	impg1	FGF2	FGFR2
DEFB131	impg2	FGF23	FGFR2
DEFB132	insr	FGF3	FGFR2
DEFB133	insrr	FGF4	FGFR2
DEFB134	ints6	FGF5	FGFR2
DEFB135	irak1	FGF6	FGFR2
DEFB136	irak3	FGF7	FGFR2
Defb14	irs1	FGF8	FGFR2
Defb15	irs3	FGF9	FGFR2
Defb18	itga1	CHGB	FGFR3
Defb19	itga10	CTSK	FGFR3
Defb2	itga11	FGF1	FGFR3
Defb20	itga2	FGF17	FGFR3
Defb25	itga2b	FGF18	FGFR3
Defb29	itga3	FGF2	FGFR3
Defb3	itga4	FGF23	FGFR3
Defb30	itga5	FGF3	FGFR3
Defb34	itga6	FGF4	FGFR3
Defb35	itga7	FGF5	FGFR3
Defb36	itga8	FGF6	FGFR3
Defb37	itga9	FGF7	FGFR3
Defb38	itgad	FGF8	FGFR3
Defb39	itgae	FGF9	FGFR3
Defb4	itgal	FGF1	FGFR4
Defb40	itgam	FGF17	FGFR4
Defb41	itgav	FGF18	FGFR4
Defb43	itgax	FGF19	FGFR4
DEFB4B	itgb1	FGF2	FGFR4
Defb50	itgb2	FGF3	FGFR4
Defb6	itgb2l	FGF4	FGFR4
Defb7	itgb3	FGF5	FGFR4
Defb8	itgb4	FGF6	FGFR4
Defb9	itgb5	FGF7	FGFR4
DGCR6	itgb6	FGF8	FGFR4
Dhh	itgb7	FGF9	FGFR4
Dhrs11	itgb8	FGF2	FGFRL1
Dhrs13	itgbl1	LDLR	FLT1
DHRS4L2	itpr1	NRP1	FLT1
Dhrs7c	itpr2	PGF	FLT1
DKFZp586F0824	itpr3	VEGFA	FLT1
DKFZp686G21263	jmjd6	VEGFB	FLT1
Dkk1	kdelr1	FLT3LG	FLT3
Dkk2	kdelr2	FIGF	FLT4
Dkk3	kdelr3	ITGB1	FLT4
Dkk4	kdr	VEGFC	FLT4

Dkkl1	kir2dl1	ANXA1	FPR1
DLK1	kir2dl2	SAA1	FPR1
DLL1	kir2dl3	CAMP	FPR2
DMBT1	kir2dl4	FGFR1	FRS3
Dmkn	kir2dl5a	KARS	FRS3
Dmp1	kir2dl5b	CGA	FSHR
Dnajc10	kir2ds1	FSHB	FSHR
Dnase1	kir2ds2	WNT2	FZD1
Dnase1l2	kir2ds3	WNT3	FZD1
Dnase2b	kir2ds4	WNT3A	FZD1
DPP4	kir2ds5	WNT5A	FZD1
Dpp7	kir3dl1	NDP	FZD4
Dpt	kir3dl2	WNT5A	FZD5
Dpysl3	kir3dl3	WNT7A	FZD5
Dsc3	kir3ds1	SFRP1	FZD6
DSCAM	kiss1r	WNT4	FZD6
Dspp	kit	WNT1	FZD8
DST	klra1	WNT1	FZD9
EBI3	klra12	WNT2	FZD9
Ecm1	klra14	WNT7A	FZD9
Ecm2	klra15	ALB	GABBR1
Eda	klra16	APP	GABBR1
EDDM3A	klra17	GABBR1	GABBR2
EDDM3B	klra18	GABBR1	GABRA1
EDEM2	klra19	GABRA6	GABRA1
Edil3	klra2	GABRA6	GABRA2
Edn1	klra20	GABRA6	GABRA3
Edn2	klra21	GABRA6	GABRA4
Edn3	klra22	C1QBP	GABRB1
EFEMP1	klra23	GABBR1	GABRB2
EFEMP2	klra4	GABRA6	GABRB2
Efhb	klra5	GABRA6	GABRB3
Efna1	klra6	GABBR1	GABRG2
EFNA4	klra7	GABRA6	GABRG3
EG654453	klra8	GAL	GALR1
Egf	klra9	GAL	GALR2
Egfl6	klrb1	GCG	GCGR
Egfl7	klrb1a	GNAS	GCGR
Egfl8	klrb1b	GDNF	GFRA1
Egflam	klrb1c	NCAM1	GFRA1
EGFR	klrc1	NRTN	GFRA1
ELANE	klrc2	GDNF	GFRA2
Eln	klrc3	NRTN	GFRA2
ELSPBP1	klrc4	ARTN	GFRA3
EMCN	klrd1	PSPN	GFRA4
Emid1	klre1	GH1	GHR
Emid2	klrf1	GH2	GHR

Emilin1	klrf2	GHRH	GHRHR
Emilin2	klrg1	GHRL	GHRHR
Emilin3	klri1	CORT	GHSR
EMR3	klri2	GHRL	GHSR
Enam	klrk1	GCG	GIPR
Endod1	kpg_004	GIP	GIPR
ENDOU	krt1	GCG	GLP1R
ENG	ktn1	GCG	GLP2R
Enho	lag3	GNRH1	GNRHR
Enox1	lair1	EBI3	GOSR1
Enox2	lair2	CTSG	GP1BA
ENPP1	lancl1	F11	GP1BA
ENPP2	lbr	F12	GP1BA
ENPP3	lcor	F2	GP1BA
Enpp5	ldlr	FLNA	GP1BA
ENPP6	ldlrad1	KNG1	GP1BA
Entpd1	ldlrad2	SELP	GP1BA
Entpd2	ldlrad3	VWF	GP1BA
Entpd6	lef1	VWF	GP1BB
Epdr1	leng4	CRP	GP6
EPGN	leng8	FCGR3A	GP6
EPHA10	leng9	APOA1	GPLD1
EPHA3	lepr	APOA4	GPLD1
EPHB6	lgals3bp	CD55	GPLD1
Ephx3	lgr4	GAL	GPR151
Epo	lgr5	C3	GPR77
EPOR	lgr6	C4A	GPR77
Epyc	lhcgr	HSPA5	GRIA1
Erap1	lifr	GRIA3	GRIA2
ERBB2IP	lilra1	ACTN1	GRIA4
ERBB3	lilra2	FLNA	GRIK1
Ereg	lilra3	GRIN2B	GRIK2
Es2	lilra4	FLNA	GRIK3
Esm1	lilra5	LRSAM1	GRIK5
Expi	lilra6	ACTN2	GRIN1
EXTL2	lilrb1	ACTN4	GRIN1
EYS	lilrb2	GRIN2B	GRIN1
F10	lilrb3	LRP8	GRIN1
F11	lilrb4	MAP2K2	GRIN1
F12	lilrb5	PLAT	GRIN1
F13a1	lmbr1	SLMAP	GRIN1
F13B	lmbr1l	STX1A	GRIN1
F2	lnpep	TUBA4A	GRIN1
F2R	loxl2	ACTN1	GRIN2A
F2RL2	loxl3	GRIN2B	GRIN2A
F2RL3	loxl4	IL16	GRIN2A
F3	lpar1	ACTN2	GRIN2B

F5	lpar2	ACTN4	GRIN2B
F7	lpar3	CAPZA2	GRIN2B
F8	lpar4	ERBB2IP	GRIN2B
F8VSI5	lpar5	GNAS	GRIN2B
F8VX64	lpar6	GSN	GRIN2B
F8WCM5	lphn1	IL16	GRIN2B
F9	lphn2	LGI1	GRIN2B
Fam108a	lphn3	MAP2K2	GRIN2B
FAM108A1	lrp1	SPINK5	GRIN2B
Fam108b	lrp10	STX1A	GRIN2B
FAM108B1	lrp11	VEGFA	GRIN2B
Fam131a	lrp12	ERBB2IP	GRIN2C
Fam132a	lrp1b	IL16	GRIN2C
Fam132b	lrp2	IL16	GRIN2D
FAM150A	lrp3	MAP2K2	GRIN2D
FAM150B	lrp4	GRIN2B	GRIN3A
Fam172a	lrp5	SNCA	GRK1
Fam180a	lrp5l	SNCA	GRK5
Fam198a	lrp6	PRSS23	GRK6
Fam19a1	lrp8	SNCA	GRK6
Fam19a3	lrpap1	PTPRZ1	GRLF1
Fam19a4	lrrn2	GRIN2B	GRM1
Fam19a5	lsr	FLNA	GRM4
Fam20a	ltb4r	FLNA	GRM5
Fam20c	ltb4r1	GRIN2B	GRM5
Fam24a	ltb4r2	FLNA	GRM7
FAM24B	ltbp1	TUBA4A	GRM7
Fam3a	ltbp4	FLNA	GRM8
Fam3b	ltbr	GRP	GRPR
Fam3c	ltk	HSP90AA1	GUCY1B3
FAM3D	ly75	GUCA2A	GUCY2C
FAM55A	ly9	GUCA1B	GUCY2D
FAM55C	ly96	GUCA2B	GUCY2D
Fam55d	lyve1	S100B	GUCY2D
Fam5b	m6pr	TUBA4A	GUCY2D
Fam5c	marco	GUCA1B	GUCY2F
FAS	mas1	EGFR	HBEGF
Fasl	mas1l	FBLN1	HBEGF
FASLG	mc1r	LTBP3	HBEGF
Fbln1	mc2r	MMP7	HBEGF
FBLN2	mc3r	HCRT	HCRTR1
Fbln5	mc4r	HCRT	HCRTR2
Fbln7	mc5r	HABP2	HNF4A
Fbn1	mcc	SERPINA1	HNF4A
Fbn2	mchr1	VTN	HNF4A
FBN3	mchr2	F7	HPN
FCAR	med1	HGF	HPN

FCER2	med12	IL6	HRH1
FCGBP	med13	CCL16	HRH4
FCGR3A	med14	PON2	HTR2A
FCGR3B	med16	HSPA5	HTR3A
FCN1	med17	GNAS	HTR6
FCN2	med24	EGFR	ICAM1
FCN3	med30	FGG	ICAM1
Fcna	med4	SPN	ICAM1
Fcnb	mertk	IFNA2	IFNAR1
Fcrla	met	IFNA8	IFNAR1
FDCSP	mfsd6	IFNAR2	IFNAR1
Fetub	mlnr	IFNB1	IFNAR1
Fga	mmd	IFNW1	IFNAR1
Fgb	mmd2	IFNA1	IFNAR2
Fgf1	mpl	IFNA2	IFNAR2
Fgf10	mr1	IFNA5	IFNAR2
FGF12	mrc1	IFNA8	IFNAR2
Fgf15	mrc2	IFNB1	IFNAR2
Fgf16	mrgpra1	IL10	IFNAR2
Fgf17	mrgpra3	IL22	IFNAR2
Fgf18	mrgpra4	IFNG	IFNGR1
FGF19	mrgpra5	ANXA5	IFNGR2
Fgf2	mrgpra6	IFNG	IFNGR2
Fgf20	mrgpra7	EGFR	IGF1R
Fgf21	mrgpra8	IGF1	IGF1R
Fgf22	mrgprb1	IGF2	IGF1R
Fgf23	mrgprb2	IGFBP3	IGF1R
FGF3	mrgprb3	INS	IGF1R
Fgf4	mrgprb4	ITGB1	IGF1R
Fgf5	mrgprb5	WISP2	IGF1R
Fgf6	mrgprb8	CREG1	IGF2R
Fgf7	mrgprd	CTSD	IGF2R
Fgf8	mrgpre	IGF2	IGF2R
Fgf9	mrgprf	PLAU	IGF2R
Fgfbp1	mrgprg	PLAUR	IGF2R
FGFBP2	mrgprh	WISP2	IGF2R
Fgfbp3	mrgprx1	IFNAR2	IL10RA
FGFR1	mrgprx2	IL10	IL10RA
FGFR2	mrgprx3	IL10	IL10RB
FGFR4	mrgprx4	IL22	IL10RB
Fgg	ms4a10	IL28A	IL10RB
Fgl1	ms4a12	IL28B	IL10RB
Fgl2	ms4a13	IL29	IL10RB
Fhad1	ms4a14	UCN2	IL10RB
Fibcd1	ms4a15	UCN3	IL10RB
Fibin	ms4a2	IL11	IL11RA
Figf	ms4a3	IL6ST	IL11RA

Fjx1	ms4a4a	IL11	IL11RA1
Fkrp	ms4a4d	IL12A	IL12B
FKTN	ms4a4e	IL23A	IL12B
FLNA	ms4a5	IL12A	IL12RB1
FLRT1	ms4a6a	IL12B	IL12RB1
FLRT2	ms4a6b	IL23A	IL12RB1
FLRT3	ms4a6c	IL12A	IL12RB2
FLT1	ms4a6d	IL12B	IL12RB2
Flt3l	ms4a6e	IL13	IL13RA1
FLT3LG	ms4a7	IL4	IL13RA1
Fmo2	ms4a8a	IL4R	IL13RA1
Fmod	ms4a8b	IL13	IL13RA2
Fn1	msr1	IL4	IL13RA2
FNDC1	mst1r	IL15	IL15RA
Fndc7	mtnr1a	IL17A	IL17RA
FOLR1	mtnr1b	IL17F	IL17RA
FOLR2	mup12	IL25	IL17RA
FOLR3	musk	IL17B	IL17RB
FOLR4	myd88	IL25	IL17RB
FP248	narg2	IL17A	IL17RC
Fras1	ncoa3	IL17F	IL17RC
Frem1	ncoa4	FGFR1	IL17RD
Frem2	ncoa5	IL17RB	IL17RD
Frem3	ncoa6	IL18	IL18R1
FRZB	ncoa7	IL1RAP	IL18R1
Fshb	ncor1	IL18	IL18RAP
Fst	ncr1	FREM1	IL1R1
Fstl1	ncr2	IL1A	IL1R1
Fstl3	ncr3	IL1B	IL1R1
Fstl4	neo1	IL1F10	IL1R1
Fstl5	neto1	IL1RAP	IL1R1
Fuca2	neto2	IL1RN	IL1R1
Furin	nfam1	IL1A	IL1R2
GABBR1	ngfr	IL1B	IL1R2
GABRA6	niacr1	IL1RAP	IL1R2
Gal	nipal4	IL1RN	IL1R2
Galnt1	nisch	IL1A	IL1RAP
Galnt2	nkx3-1	IL1B	IL1RAP
Galp	nlgn1	IL1RL1	IL1RAP
Gas6	nlgn2	IL1RAP	IL1RL1
GAST	nlgn3	IL33	IL1RL1
Gbp1	nlgn4l	IL18	IL1RL2
Gbp2	nlgn4x	IL19	IL20RA
Gbp3	nlgn4y	IL20	IL20RA
Gbp6	nmbr	IL24	IL20RA
Gbp7	nmur1	IL26	IL20RA
Gbp9	nmur2	IL19	IL20RB

Gc	notch1	IL20	IL20RB
Gcg	notch2	IL24	IL20RB
Gcnt1	notch3	IL2	IL21R
Gdf1	notch4	IL21	IL21R
Gdf10	npbwr1	IL22	IL22RA1
Gdf11	npbwr2	IL22	IL22RA2
GDF15	npc1	IL12B	IL23R
Gdf2	npc1l1	IL23A	IL23R
Gdf3	npffr1	EBI3	IL27RA
Gdf5	npffr2	IL27	IL27RA
Gdf6	npr1	IL28A	IL28RA
Gdf7	npr2	IL28B	IL28RA
Gdf9	npr3	IL29	IL28RA
Gdnf	npsr1	ICAM1	IL2RA
GFER	nptxr	IL2	IL2RA
Gfod1	npvf	ECM1	IL2RB
Gfod2	npy	IL15	IL2RB
GFRA4	npy1r	IL15RA	IL2RB
Ggh	npy2r	IL2	IL2RB
Ggt1	npy5r	ICAM1	IL2RG
Gh	npy6r	IL15	IL2RG
GH1	nr0b1	IL15RA	IL2RG
GH2	nr0b2	IL2	IL2RG
GHR	nr1d1	IL21	IL2RG
Ghrh	nr1d2	IL4	IL2RG
Ghrl	nr1h2	IL4R	IL2RG
Gif	nr1h3	IL7	IL2RG
GIP	nr1h4	IL7R	IL2RG
Gkn1	nr1h5	IL9	IL2RG
Gkn2	nr1i2	IL9R	IL2RG
Gkn3	nr1i3	IL6ST	IL31RA
GKN3P	nr2c1	LIFR	IL31RA
Gla	nr2c2	CSF2	IL3RA
Glb1l	nr2c2ap	EPOR	IL3RA
Glb1l2	nr2e1	IL3	IL3RA
Gldn	nr2e3	CD40	IL4R
Glg1	nr2f1	IL13	IL4R
Glipr1	nr2f2	IL4	IL4R
GLIPR1L1	nr2f6	IL5	IL5RA
Glipr2	nr3c1	CNTF	IL6R
Glt1d1	nr3c2	ERAP1	IL6R
Glycam1	nr4a1	IL6	IL6R
Gm12597	nr4a2	IL6ST	IL6R
Gm128	nr4a3	IL6ST	IL6RA
Gm13271	nr5a1	CNTF	IL6ST
Gm13275	nr5a2	CTF1	IL6ST
Gm13276	nr6a1	ERBB3	IL6ST

Gm13280	nradd	IL11	IL6ST
Gm13283	nrbf2	IL6	IL6ST
Gm15386	nrg2	IL6R	IL6ST
Gm1673	nrip1	LIF	IL6ST
Gm17365	nrp1	LIFR	IL6ST
Gm46	nrp2	OSM	IL6ST
Gm5077	nrxn1	PLAUR	IL6ST
Gm525	nrxn3	ALB	IL7R
Gm6484	ntrk1	APOA1	IL7R
Gm749	ntrk2	CALR	IL7R
Gm885	ntrk3	CAPZA1	IL7R
Gm94	ntsr1	CRLF2	IL7R
Gnas	ntsr2	F13A1	IL7R
GNG8	nus1	FGG	IL7R
GNGT2	o3far1	GSN	IL7R
GNL1	ogfr	HSPA5	IL7R
GNLY	ogfrl1	HSPA8	IL7R
Gnptg	olfr1	HSPD1	IL7R
Gnrh1	olfr10	IL7	IL7R
GNRH2	olfr100	LGALS1	IL7R
Gp2	olfr1000	P4HB	IL7R
Gpc1	olfr1002	PEBP1	IL7R
Gpc2	olfr1006	PFN1	IL7R
Gpc3	olfr1008	PLEK	IL7R
Gpc4	olfr1009	PPBP	IL7R
Gpc5	olfr101	SOD1	IL7R
Gpc6	olfr1010	TF	IL7R
Gpha2	olfr1012	TSLP	IL7R
Gphb5	olfr1013	TTR	IL7R
GPI	olfr1014	TUBA4A	IL7R
Gpi1	olfr1015	TXN	IL7R
Gpihbp1	olfr1016	VCL	IL7R
GPLD1	olfr1018	IL9	IL9R
Gpx3	olfr1019	AHSG	INSR
GPX5	olfr102	CEACAM1	INSR
GPX6	olfr1020	ENPP1	INSR
Gpx7	olfr1022	IGF2	INSR
Grem1	olfr1023	INS	INSR
Grem2	olfr1024	ANXA1	IRAK1
GRIA3	olfr1026	DKK3	IRAK1
GRIN2B	olfr1028	DLK1	IRAK1
Grn	olfr1029	FBLN2	IRAK1
Grp	olfr103	HSP90AA1	IRAK1
Gsn	olfr1030	HSPA8	IRAK1
Gstm1	olfr1031	IL10	IRAK1
GUCA1B	olfr1032	IL1RAP	IRAK1
Guca2a	olfr1033	IL1RL1	IRAK1

Guca2b	olfr1034	NGFR	IRAK1
Gzma	olfr1036	NOTCH2	IRAK1
GZMK	olfr1037	COL6A3	ITGA1
GZMM	olfr1039	COL8A1	ITGA1
H3F3B	olfr1040	ITGB1	ITGA1
H47	olfr1042	LAMA1	ITGA1
Habp2	olfr1043	LGALS8	ITGA1
HABP4	olfr1044	MATN1	ITGA1
Hamp	olfr1045	TGM2	ITGA1
Hamp2	olfr1046	TLN1	ITGA1
Hapln1	olfr1047	ITGB1	ITGA10
Hapln2	olfr1048	ITGB1	ITGA11
Hapln3	olfr1049	ALB	ITGA2
Hapln4	olfr1051	CD46	ITGA2
Hbegf	olfr1052	CD47	ITGA2
Hc	olfr1053	CHAD	ITGA2
HCG22	olfr1054	COL1A1	ITGA2
HCRT	olfr1055	COL1A2	ITGA2
Hdgf	olfr1056	COL6A3	ITGA2
Hdlbp	olfr1057	COL8A1	ITGA2
HEBP1	olfr1058	HSPG2	ITGA2
HEG1	olfr1060	ITGB1	ITGA2
Hfe2	olfr1061	LAMA1	ITGA2
Hgf	olfr1062	MMP1	ITGA2
Hgfac	olfr1065	CALR	ITGA2B
hGH-V	olfr1066	CD47	ITGA2B
Hhip	olfr107	COL1A2	ITGA2B
HHIPL1	olfr1071	COL2A1	ITGA2B
Hhipl2	olfr1076	CTSG	ITGA2B
Hhla1	olfr1079	F2	ITGA2B
HIG2	olfr108	FGA	ITGA2B
HIST1H3G	olfr1080	IL7R	ITGA2B
HIST1H4K	olfr1082	TGM2	ITGA2B
HIST2H3A	olfr1084	TLN1	ITGA2B
HLA-C	olfr1085	VWF	ITGA2B
Hmcn1	olfr1086	ADAM9	ITGA3
Hmcn2	olfr1087	CALR	ITGA3
Hmgb1	olfr1089	FN1	ITGA3
Hmgb2	olfr109	ITGB1	ITGA3
HMOX1	olfr1090	LAMA3	ITGA3
HMSD	olfr1093	LAMA4	ITGA3
Hp	olfr1094	LGALS8	ITGA3
HPR	olfr1095	TGM2	ITGA3
Hpse	olfr1097	THBS1	ITGA3
Hpx	olfr1098	TIMP2	ITGA3
Hrg	olfr1099	ADAM28	ITGA4
HS3ST4	olfr11	CD47	ITGA4

HSD11B1L	olfr110	EMILIN1	ITGA4
Hsd17b11	olfr1100	FN1	ITGA4
Hsd17b12	olfr1101	ICAM4	ITGA4
HSD17B13	olfr1102	ITGB1	ITGA4
Hsp90aa1	olfr1104	LGALS8	ITGA4
HSPA5	olfr1105	LGALS9	ITGA4
Hspa8	olfr1106	TGFB1I1	ITGA4
Hspd1	olfr1107	THBS1	ITGA4
Hspg2	olfr1109	THBS2	ITGA4
Hsph1	olfr111	VCAM1	ITGA4
HTN1	olfr1110	VCAN	ITGA4
HTN3	olfr1111	ANGPT1	ITGA5
Htra1	olfr1112	ANGPTL3	ITGA5
Htra3	olfr1113	CD40LG	ITGA5
HTRA4	olfr1115	COL18A1	ITGA5
Hyal1	olfr1118	COL1A1	ITGA5
Hyal3	olfr112	COL6A3	ITGA5
Iapp	olfr1120	CTGF	ITGA5
Ibsp	olfr1121	EGFR	ITGA5
Icam1	olfr1122	F2	ITGA5
Icam4	olfr1123	FGFR2	ITGA5
ICOS	olfr1124	FN1	ITGA5
Ide	olfr1126	IGFBP2	ITGA5
Ifi30	olfr1128	ITGB1	ITGA5
Ifna1	olfr1129	LAMA4	ITGA5
IFNA10	olfr113	LGALS8	ITGA5
Ifna11	olfr1130	LGALS9	ITGA5
Ifna12	olfr1131	MMP9	ITGA5
Ifna13	olfr1132	PECAM1	ITGA5
Ifna14	olfr1133	SFRP2	ITGA5
IFNA16	olfr1134	SPP1	ITGA5
IFNA17	olfr1135	TGM2	ITGA5
Ifna2	olfr1136	TNC	ITGA5
IFNA21	olfr1137	ADAM9	ITGA6
Ifna4	olfr1138	COL17A1	ITGA6
Ifna5	olfr114	ITGB1	ITGA6
Ifna6	olfr1140	LAMA2	ITGA6
Ifna7	olfr1141	LAMA5	ITGA6
IFNA8	olfr1143	LAMB1	ITGA6
Ifna9	olfr1145	LAMB2	ITGA6
Ifnab	olfr1148	LAMB3	ITGA6
IFNAR2	olfr115	LAMC1	ITGA6
Ifnb1	olfr1151	PCSK5	ITGA6
Ifne	olfr1152	ITGB1	ITGA7
Ifng	olfr1153	MYOC	ITGA7
Ifnk	olfr1154	FN1	ITGA8
IFNW1	olfr1155	ITGB1	ITGA8

Ifnz	olfr1156	NPNT	ITGA8
Igf1	olfr1157	TNC	ITGA8
Igf2	olfr1158	VTN	ITGA8
Igfals	olfr116	ADAM12	ITGA9
Igfbp1	olfr1160	ADAM9	ITGA9
Igfbp2	olfr1161	FIGF	ITGA9
Igfbp3	olfr1162	ITGB1	ITGA9
Igfbp4	olfr1163	SPP1	ITGA9
Igfbp5	olfr1164	TNC	ITGA9
Igfbp6	olfr1166	VCAM1	ITGA9
Igfbp7	olfr1167	CYR61	ITGAD
Igfbpl1	olfr1168	PLG	ITGAD
IGFL1	olfr117	VCAM1	ITGAD
IGFL2	olfr1170	VTN	ITGAD
Igfl3	olfr1173	ESM1	ITGAL
IGFL4	olfr1176	ICAM1	ITGAL
IGHD	olfr1178	ICAM4	ITGAL
IGHG1	olfr1179	LGALS8	ITGAL
IGHG2	olfr118	LGALS9	ITGAL
IGHG3	olfr1180	C3	ITGAM
IGHG4	olfr1181	CEACAM8	ITGAM
IGHM	olfr1182	CFH	ITGAM
IGIP	olfr1183	CLEC4M	ITGAM
Igj	olfr1184	ELANE	ITGAM
IGKC	olfr1186	FGG	ITGAM
IGKV1-5	olfr1188	HP	ITGAM
IGKV4-1	olfr1189	ICAM1	ITGAM
IGLC1	olfr119	ICAM4	ITGAM
IGLC2	olfr1193	JAM3	ITGAM
IGLC3	olfr1195	LGALS3	ITGAM
IGLC6	olfr1196	PLAUR	ITGAM
IGLC7	olfr1197	PRTN3	ITGAM
IGLL1	olfr1198	TGFBI	ITGAM
IGLL5	olfr1199	ADAM23	ITGAV
Iglon5	olfr12	ADAM9	ITGAV
IGSF1	olfr120	ANGPTL3	ITGAV
IGSF10	olfr1200	AZGP1	ITGAV
Igsf21	olfr1201	CALR	ITGAV
Ihh	olfr1202	CD47	ITGAV
Iigp1	olfr1204	COL4A3	ITGAV
IK	olfr1205	CYR61	ITGAV
Il10	olfr1206	EDIL3	ITGAV
Il11	olfr1208	F2R	ITGAV
Il12a	olfr1209	FCER2	ITGAV
IL12B	olfr121	FN1	ITGAV
Il13	olfr1211	GSN	ITGAV
IL13RA2	olfr1212	ICAM4	ITGAV

Il15	olfr1213	ITGB1	ITGAV
IL15RA	olfr1214	LGALS8	ITGAV
Il16	olfr1215	MMP14	ITGAV
Il17a	olfr1216	MMP2	ITGAV
Il17b	olfr1217	NID1	ITGAV
IL17C	olfr1218	NOV	ITGAV
IL17D	olfr1219	PLAUR	ITGAV
Il17f	olfr122	SPP1	ITGAV
IL17RB	olfr1220	TGFB1	ITGAV
IL17RE	olfr1221	TGFB3	ITGAV
Il18	olfr1222	THBS1	ITGAV
Il18bp	olfr1223	VTN	ITGAV
Il19	olfr1225	C3	ITGAX
Il1a	olfr1226	FCER2	ITGAX
Il1b	olfr1228	ICAM4	ITGAX
Il1f10	olfr1229	JAM3	ITGAX
Il1f5	olfr123	ACTN1	ITGB1
Il1f6	olfr1230	ACTN4	ITGB1
Il1f8	olfr1231	ADAM12	ITGB1
Il1f9	olfr1232	ADAM9	ITGB1
IL1RAP	olfr1233	CD46	ITGB1
IL1RL1	olfr1234	CD47	ITGB1
IL1RN	olfr1238	CHAD	ITGB1
Il2	olfr1239	COL1A1	ITGB1
Il20	olfr124	COL6A3	ITGB1
Il21	olfr1240	EMILIN1	ITGB1
Il22	olfr1241	FBLN1	ITGB1
IL22RA2	olfr1242	FIGF	ITGB1
Il23a	olfr1243	FLNA	ITGB1
Il24	olfr1245	FN1	ITGB1
IL25	olfr1246	HSPG2	ITGB1
IL26	olfr1247	ICAM4	ITGB1
Il27	olfr1248	IGFBP2	ITGB1
IL28A	olfr1249	ITGA6	ITGB1
Il28b	olfr125	LAMA1	ITGB1
IL29	olfr1250	LAMA4	ITGB1
Il3	olfr1251	LGALS1	ITGB1
Il31	olfr1252	LGALS3	ITGB1
IL32	olfr1253	LGALS3BP	ITGB1
Il33	olfr1254	LGALS8	ITGB1
Il34	olfr1255	LGALS9	ITGB1
IL36A	olfr1256	LTB	ITGB1
IL36B	olfr1257	NID1	ITGB1
IL36G	olfr1258	NOV	ITGB1
IL36RN	olfr1259	PECAM1	ITGB1
IL37	olfr126	SPARC	ITGB1
Il4	olfr1260	SPP1	ITGB1

IL4R	olfr1261	TGM2	ITGB1
Il5	olfr1262	THBS1	ITGB1
IL5RA	olfr1263	THBS2	ITGB1
Il6	olfr1264	TLN1	ITGB1
IL6R	olfr1265	TNC	ITGB1
IL6ST	olfr1269	VCAM1	ITGB1
Il7	olfr127	VCAN	ITGB1
IL7R	olfr1270	VEGFB	ITGB1
IL8	olfr1271	C3	ITGB2
Il9	olfr1272	CD14	ITGB2
IL9R	olfr1275	ESM1	ITGB2
IMPG1	olfr1276	FCER2	ITGB2
IMPG2	olfr1277	HP	ITGB2
Inha	olfr1278	ICAM1	ITGB2
Inhba	olfr1279	ICAM4	ITGB2
Inhbb	olfr128	KNG1	ITGB2
Inhbc	olfr1280	PLAUR	ITGB2
Inhbe	olfr1281	PRTN3	ITGB2
INS	olfr1282	TLN1	ITGB2
Ins1	olfr1283	ADAM23	ITGB3
Ins2	olfr1284	ANGPTL3	ITGB3
Insl3	olfr1285	CD47	ITGB3
INSL4	olfr1286	COL1A2	ITGB3
Insl5	olfr1287	COL4A3	ITGB3
Insl6	olfr1288	COL6A3	ITGB3
Irgm2	olfr1289	CTSG	ITGB3
Isg15	olfr129	F2	ITGB3
Islr	olfr1290	FBLN2	ITGB3
Ism1	olfr1294	FCER2	ITGB3
ISM2	olfr1295	FGA	ITGB3
Itfg1	olfr1297	FGG	ITGB3
ITGA6	olfr1298	FLNA	ITGB3
ITGB1	olfr1299	FN1	ITGB3
ITGBL1	olfr13	GSN	ITGB3
Itih1	olfr130	ITGB1	ITGB3
Itih2	olfr1301	LAMA4	ITGB3
Itih3	olfr1302	NID1	ITGB3
ITIH4	olfr1303	NOV	ITGB3
Itih5	olfr1305	PECAM1	ITGB3
ITIH5L	olfr1306	SPP1	ITGB3
Itln1	olfr1307	TGM2	ITGB3
ITLN2	olfr1308	THBS1	ITGB3
Itlnb	olfr1309	TLN1	ITGB3
ITM2B	olfr131	VTN	ITGB3
IZUMO4	olfr1310	CLCA2	ITGB4
JAG1	olfr1311	COL17A1	ITGB4
Jam3	olfr1312	EGFR	ITGB4

KAL1	olfr1313	ERBB2IP	ITGB4
Kap	olfr1314	FLNA	ITGB4
KARS	olfr1316	ITGA6	ITGB4
Kazald1	olfr1317	LAMA5	ITGB4
Kcp	olfr1318	LAMB1	ITGB4
KDSR	olfr132	LAMB2	ITGB4
Kera	olfr1320	LAMC1	ITGB4
KGFLP1	olfr1321	LGALS3	ITGB4
KGFLP2	olfr1322	MET	ITGB4
KIAA0100	olfr1323	ADAM9	ITGB5
KIAA0564	olfr1324	ALB	ITGB5
KIR3DP1	olfr1325	ANXA5	ITGB5
KIR3DX1	olfr1328	CYR61	ITGB5
Kirrel3	olfr1329	FLNA	ITGB5
Kiss1	olfr133	ICAM4	ITGB5
KIT	olfr1330	ITGB1	ITGB5
Kitl	olfr1331	LTBP1	ITGB5
KITLG	olfr1333	LTBP3	ITGB5
Kl	olfr1335	PLAUR	ITGB5
Klhl11	olfr1336	SPP1	ITGB5
KLK10	olfr1337	TLN1	ITGB5
Klk11	olfr1338	VTN	ITGB5
KLK12	olfr1339	FLNA	ITGB6
Klk13	olfr134	FN1	ITGB6
Klk14	olfr1340	SPP1	ITGB6
KLK15	olfr1341	TGFB1	ITGB6
Klk1b21	olfr1342	TGFB3	ITGB6
KLK3	olfr1344	TNC	ITGB6
KLK4	olfr1346	FLNA	ITGB7
KLK5	olfr1347	FN1	ITGB7
Klk6	olfr1348	TLN1	ITGB7
Klk7	olfr1349	VCAM1	ITGB7
Klk8	olfr135	MMP14	ITGB8
KLK9	olfr1350	TGFB1	ITGB8
Klkb1	olfr1351	VTN	ITGB8
Kng1	olfr1352	ANXA5	KDR
KRTAP1-1	olfr1353	COL18A1	KDR
KRTAP1-3	olfr1354	FIGF	KDR
KRTAP5-8	olfr1355	FLT1	KDR
Krtdap	olfr1356	HSP90AA1	KDR
LACRT	olfr1357	NRP1	KDR
Lad1	olfr1358	TIMP3	KDR
LAIR2	olfr1359	VEGFA	KDR
Lalba	olfr136	VEGFC	KDR
Lama1	olfr1360	KISS1	KISS1R
Lama2	olfr1361	CSF2RA	KIT
Lama3	olfr1362	EPOR	KIT

Lama4	olfr1364	KITLG	KIT
Lama5	olfr1366	MICA	KLRK1
Lamb1	olfr1367	ULBP2	KLRK1
Lamb2	olfr1368	APOB	LDLR
Lamb3	olfr137	APOE	LDLR
LAMB4	olfr1370	FLT1	LDLR
Lamc1	olfr1371	HSPA5	LDLR
Lamc2	olfr1373	PCSK9	LDLR
Lamc3	olfr1377	PF4	LDLR
Lao1	olfr1378	APOD	LEPR
LATH	olfr138	CLU	LEPR
Lbp	olfr1380	LEP	LEPR
Lcat	olfr1381	CD14	LGALS3BP
LCN1	olfr1382	CD248	LGALS3BP
Lcn10	olfr1383	COL5A1	LGALS3BP
Lcn12	olfr1384	COL6A1	LGALS3BP
Lcn13	olfr1385	FN1	LGALS3BP
LCN15	olfr1386	ITGB1	LGALS3BP
LCN1P1	olfr1387	LGALS1	LGALS3BP
Lcn2	olfr1388	LGALS3	LGALS3BP
Lcn3	olfr1389	NID1	LGALS3BP
Lcn4	olfr139	VCL	LGALS3BP
Lcn5	olfr1390	CGA	LHCGR
LCN6	olfr1391	GNAS	LHCGR
Lcn8	olfr1392	LHB	LHCGR
Lcn9	olfr1393	CNTF	LIFR
LDLR	olfr1394	CTF1	LIFR
Leap2	olfr1395	IL6ST	LIFR
Lect1	olfr1396	LIF	LIFR
Lect2	olfr140	OSM	LIFR
Lefty1	olfr1402	B2M	LILRB1
Lefty2	olfr1404	LCN1	LMBR1L
Lep	olfr1406	SCGB1A1	LMBR1L
LEPR	olfr1408	EGFL7	LOXL2
Lepre1	olfr141	EGFL7	LOXL3
Leprel1	olfr1410	COL2A1	LOXL4
LFNG	olfr1411	EGFL7	LOXL4
Lgals1	olfr1412	A2M	LRP1
Lgals3	olfr1413	APOE	LRP1
LGALS3BP	olfr1414	APP	LRP1
LGALS7	olfr1415	C3	LRP1
LGALS8	olfr1416	C4BPA	LRP1
Lgals9	olfr1417	CALR	LRP1
Lgi1	olfr1418	CTGF	LRP1
Lgi2	olfr1419	CTSG	LRP1
Lgi3	olfr142	ELANE	LRP1
Lgi4	olfr1420	F8	LRP1

LHB	olfr1423	F9	LRP1
LHCGR	olfr1424	FURIN	LRP1
LIBC	olfr1425	LIPC	LRP1
Lif	olfr1426	LPL	LRP1
LIFR	olfr1427	LTF	LRP1
LILRA3	olfr1428	MDK	LRP1
LILRA5	olfr143	MMP13	LRP1
LINC00305	olfr1431	MMP14	LRP1
Lipc	olfr1432	MMP15	LRP1
Lipe	olfr1433	MMP16	LRP1
Lipf	olfr1434	MMP17	LRP1
Lipg	olfr1436	MMP9	LRP1
Liph	olfr1437	PDGFB	LRP1
Lipi	olfr1440	PLAT	LRP1
Lipk	olfr1441	PLAUR	LRP1
Lipm	olfr1442	SERPINA1	LRP1
Lipn	olfr1443	SERPINE1	LRP1
Lmcd1	olfr1444	THBS1	LRP1
LNPEP	olfr1445	TIMP1	LRP1
Lox	olfr1446	TLN1	LRP1
Loxl1	olfr1447	VCL	LRP1
LOXL2	olfr1448	WNT3A	LRP1
LOXL3	olfr1449	HTRA1	LRP10
LOXL4	olfr145	ACTN2	LRP12
LPA	olfr1450	APP	LRP1B
LPAL2	olfr1451	PLAT	LRP1B
Lpl	olfr1453	PLAUR	LRP1B
LPO	olfr1454	SERPINE1	LRP1B
Lrch3	olfr1457	ALB	LRP2
LRG1	olfr1458	APOB	LRP2
LRP2	olfr1459	APOE	LRP2
LRP8	olfr146	APOH	LRP2
Lrrc17	olfr1461	CLU	LRP2
LRSAM1	olfr1462	GC	LRP2
LSR	olfr1463	INS	LRP2
Lta	olfr1465	LCN2	LRP2
Ltb	olfr1466	LPA	LRP2
Ltbp1	olfr1467	LPL	LRP2
Ltbp2	olfr1469	MDK	LRP2
Ltbp3	olfr147	PLAU	LRP2
Ltbp4	olfr1471	SCGB1A1	LRP2
Ltf	olfr1472	SERPINE1	LRP2
Lum	olfr1474	TG	LRP2
Luzp2	olfr1475	TLN1	LRP2
Ly6g5b	olfr1477	TTR	LRP2
Ly6g5c	olfr148	DKK1	LRP5
Ly6k	olfr1480	THBS1	LRP5

Ly86	olfr1484	WNT1	LRP5
LY96	olfr1487	ANTXR2	LRP6
Lyg1	olfr1489	DKK1	LRP6
Lyg2	olfr149	DKK2	LRP6
Lypd6	olfr1490	WNT1	LRP6
LYZ	olfr1491	WNT3A	LRP6
Lyz1	olfr1494	APOE	LRP8
Lyz2	olfr1495	APOH	LRP8
Lyzl1	olfr1496	CLU	LRP8
LYZL2	olfr1497	RELN	LRP8
Lyzl4	olfr1499	VCL	LRRN2
Lyzl6	olfr15	FBN1	LTBP1
MAGP2	olfr150	FBN2	LTBP1
Mamdc2	olfr1500	FN1	LTBP1
Man2b2	olfr1501	IGFBP3	LTBP1
Manf	olfr1502	TGM2	LTBP1
MAP2K2	olfr1504	IGHG1	LTBP4
MAPK15	olfr1505	TGFB1	LTBP4
MARCO	olfr1506	LTA	LTBR
Masp1	olfr1507	LTB	LTBR
Masp2	olfr1508	TNF	LTBR
Matn1	olfr1509	TNFSF14	LTBR
Matn2	olfr151	LY86	LY96
Matn3	olfr1510	MBL2	LY96
Matn4	olfr1511	S100A8	LY96
Mbl1	olfr1512	SFTPA1	LY96
Mbl2	olfr1513	SFTPA2	LY96
Mcfd2	olfr152	SFTPD	LY96
Mcpt1	olfr153	REN	M6PR
Mcpt8	olfr1535	SCGB3A1	MARCO
Mdk	olfr154	SCGB3A2	MARCO
Megf6	olfr155	AGT	MAS1
MEP1A	olfr156	ASIP	MC1R
MEP1B	olfr157	POMC	MC1R
Mepe	olfr159	ASIP	MC2R
MET	olfr16	POMC	MC2R
Metrn	olfr160	AGRP	MC3R
Metrnl	olfr161	ASIP	MC3R
MFAP1	olfr164	AGRP	MC4R
Mfap1a	olfr165	ASIP	MC4R
Mfap2	olfr166	NPY	MC4R
Mfap4	olfr167	POMC	MC4R
Mfap5	olfr168	AGRP	MC5R
Mfge8	olfr169	ASIP	MC5R
Mfi2	olfr17	POMC	MC5R
MFNG	olfr170	AIMP1	MCC
Mgat4a	olfr171	AKR1B1	MCC

Mgp	olfr172	ANXA2	MCC
MIA	olfr173	CAPZA1	MCC
Mia1	olfr176	ERBB2IP	MCC
Mia2	olfr177	MAP2K2	MCC
MICA	olfr178	PNP	MCC
Mif	olfr179	SMC3	MCC
mifr-2	olfr18	TFRC	MCC
MLN	olfr180	PMCH	MCHR1
Mmel1	olfr181	PMCH	MCHR2
MMP1	olfr183	IGFBP1	MED1
Mmp10	olfr186	ACTN1	MED14
Mmp11	olfr187	ACTN2	MED14
Mmp12	olfr19	IGFBP1	MED14
Mmp13	olfr190	ISG15	MED14
Mmp14	olfr191	UACA	MED14
Mmp15	olfr192	GAS6	MERTK
Mmp16	olfr193	MSR1	MERTK
Mmp17	olfr194	EGFR	MET
Mmp19	olfr195	FAS	MET
Mmp1a	olfr196	HGF	MET
Mmp2	olfr197	HGFAC	MET
Mmp20	olfr198	PLXNB1	MET
Mmp21	olfr199	GHRL	MLNR
Mmp23	olfr2	MLN	MLNR
MMP23B	olfr20	THPO	MPL
Mmp24	olfr201	SIGLEC1	MRC1
Mmp25	olfr202	CORT	MRGPRX2
MMP26	olfr203	EPOR	MST1R
MMP27	olfr204	MST1	MST1R
MMP28	olfr205	SFN	MST1R
Mmp3	olfr206	CALR	MTNR1A
Mmp7	olfr207	CNP	MTNR1A
Mmp8	olfr208	FLNA	MTNR1A
Mmp9	olfr209	HSPA5	MTNR1A
Mmrn1	olfr211	COPA	MTNR1B
Mmrn2	olfr212	FLNA	MTNR1B
Mpo	olfr213	HSPA5	MTNR1B
MR1	olfr214	COLQ	MUSK
Msln	olfr215	CD59	NCR1
Msmb	olfr218	CD59	NCR3
Msmp	olfr220	NTN1	NEO1
MSR1	olfr221	NTN3	NEO1
Mst1	olfr222	NTN4	NEO1
MST1P9	olfr223	BDNF	NGFR
Mstn	olfr224	IL2	NGFR
MT-RNR2	olfr225	NGF	NGFR
MTRNR2L1	olfr228	NTF4	NGFR

MTRNR2L10	olfr229	NMB	NMBR
MTRNR2L2	olfr23	NMS	NMUR1
MTRNR2L3	olfr231	NMU	NMUR1
MTRNR2L4	olfr235	NMS	NMUR2
MTRNR2L5	olfr238	NMU	NMUR2
MTRNR2L6	olfr239	ADAM17	NOTCH1
MTRNR2L7	olfr24	DLL1	NOTCH1
MTRNR2L8	olfr242	FURIN	NOTCH1
MTRNR2L9	olfr243	JAG1	NOTCH1
MUC1	olfr247	LFNG	NOTCH1
Muc13	olfr248	MFNG	NOTCH1
MUC15	olfr25	NOV	NOTCH1
MUC16	olfr250	PRF1	NOTCH1
MUC17	olfr251	RBP3	NOTCH1
Muc19	olfr255	TNF	NOTCH1
Muc2	olfr256	DLK1	NOTCH2
Muc20	olfr257	DLL1	NOTCH2
MUC3A	olfr259	JAG1	NOTCH2
Muc4	olfr26	LFNG	NOTCH2
Muc5ac	olfr262	MFNG	NOTCH2
MUC5B	olfr263	MYOC	NOTCH2
Muc6	olfr266	DLL1	NOTCH3
MUC7	olfr267	JAG1	NOTCH3
MUCL1	olfr27	NPB	NPBWR1
Mug1	olfr270	NPW	NPBWR1
Mug2	olfr272	NPB	NPBWR2
Mup20	olfr273	NPW	NPBWR2
Mup3	olfr275	NPFF	NPFFR1
Mup4	olfr279	HCRT	NPFFR2
MXRA5	olfr281	NPFF	NPFFR2
Myoc	olfr282	NPY	NPFFR2
Napsa	olfr283	NPPA	NPR1
Nav2	olfr284	NPPB	NPR1
Nbl1	olfr285	SEMA3A	NPR1
NCAM1	olfr286	NPPA	NPR2
Ncan	olfr287	NPPB	NPR2
Ndfip1	olfr288	NPPC	NPR2
NDNF	olfr290	NPPA	NPR3
Ndp	olfr291	NPPB	NPR3
Nell1	olfr292	NPPC	NPR3
Nell2	olfr293	NPTX2	NPTXR
Nenf	olfr294	GHRL	NPY
Nepn	olfr295	MEP1A	NPY
NETO1	olfr297	MEP1B	NPY
Ngf	olfr298	NPFF	NPY
NGFR	olfr299	PMCH	NPY
Ngrn	olfr3	NPY	NPY1R

NHLRC3	olfr30	PYY	NPY1R
Nid1	olfr301	NPY	NPY2R
Nid2	olfr302	PYY	NPY2R
Nmb	olfr303	NPY	NPY5R
Nms	olfr304	PYY	NPY5R
Nmu	olfr305	ACTN1	NR1H3
Nodal	olfr307	HSP90AA1	NR2C2
Nog	olfr308	PEBP1	NR2C2
NOTCH1	olfr309	ERBB2IP	NR2E1
NOTCH2	olfr31	ANGPTL1	NR2F6
NOTCH2NL	olfr310	ADA	NR3C1
NOTCH3	olfr311	CALR	NR3C1
NOTCH4	olfr312	GZMA	NR3C1
Notum	olfr313	HMGB1	NR3C1
Nov	olfr314	HMGB2	NR3C1
Npb	olfr315	HSP90AA1	NR3C1
Npc2	olfr316	HSPD1	NR3C1
Npff	olfr317	IDE	NR3C1
NPIPL1	olfr318	IGFBP1	NR3C1
NPIPL2	olfr319	IL8	NR3C1
Npnt	olfr32	MAPK15	NR3C1
NPPA	olfr320	MMP1	NR3C1
Nppb	olfr323	MMP13	NR3C1
Nppc	olfr324	SFN	NR3C1
Nps	olfr325	TGFB1I1	NR3C1
Nptx2	olfr328	TXN	NR3C1
NPTXR	olfr33	HSP90AA1	NR3C2
Npvf	olfr330	LHB	NR5A1
NPW	olfr331	ERBB3	NRG2
Npy	olfr332	ACTN2	NRIP1
Nrg1	olfr338	FGF1	NRP1
NRG2	olfr339	FGF2	NRP1
Nrg3	olfr340	FGF4	NRP1
Nrg4	olfr341	FGF7	NRP1
NRP1	olfr342	FGFR1	NRP1
Nrtn	olfr344	FLT1	NRP1
Ntf3	olfr345	PGF	NRP1
NTF4	olfr346	SEMA3A	NRP1
Ntf5	olfr347	SEMA3B	NRP1
Ntm	olfr348	SEMA3C	NRP1
Ntn1	olfr350	SEMA3D	NRP1
Ntn3	olfr351	SEMA3F	NRP1
Ntn4	olfr352	VEGFA	NRP1
Ntn5	olfr353	VEGFB	NRP1
Nts	olfr354	FLT1	NRP2
NUCB1	olfr355	NRP1	NRP2
NUCB2	olfr356	PGF	NRP2

Nxph1	olfr357	SEMA3B	NRP2
Nxph2	olfr358	SEMA3C	NRP2
Nxph3	olfr360	SEMA3F	NRP2
NXPH4	olfr361	VEGFA	NRP2
Nyx	olfr362	NXPH1	NRXN1
OBP2A	olfr365	NXPH2	NRXN1
OBP2B	olfr366	NXPH3	NRXN1
Oc90	olfr368	PDZD2	NRXN1
Odam	olfr370	KLK3	NTRK1
ODZ1	olfr371	NGF	NTRK1
Ogn	olfr372	NGFR	NTRK1
Oit1	olfr373	NTF3	NTRK1
Olfm1	olfr374	BDNF	NTRK2
Olfm2	olfr376	NGFR	NTRK2
Olfm3	olfr378	NTF3	NTRK2
Olfm4	olfr38	NTF4	NTRK2
OLFML1	olfr380	NGFR	NTRK3
Olfml2a	olfr381	NTF3	NTRK3
Olfml2b	olfr382	NTS	NTSR1
Olfml3	olfr384	NTS	NTSR2
OLR1	olfr385	PNOC	OPRL1
Omd	olfr389	FLNA	OPRM1
Oosp1	olfr39	OBP2A	OR1G1
Optc	olfr390	ALB	OR2T6
Orm1	olfr392	ALB	OR3A2
Orm2	olfr393	IGHM	OR5H6
OSCAR	olfr394	ALB	OR8D2
Osm	olfr395	IGHG1	OR8D2
Ostn	olfr397	IL6ST	OSMR
Otoa	olfr398	OSM	OSMR
Otog	olfr399	AVP	OXTR
Otol1	olfr401	OXT	OXTR
Otop1	olfr402	CAMP	P2RX7
Otor	olfr403	CD47	P2RY2
Otos	olfr410	PDGFA	PDGFRA
OVCH1	olfr411	PDGFB	PDGFRA
Ovch2	olfr412	PDGFC	PDGFRA
OVOS1	olfr414	COPA	PDGFRB
OVOS2	olfr417	EGFR	PDGFRB
Oxt	olfr419	PDAP1	PDGFRB
P4ha2	olfr420	PDGFB	PDGFRB
P4HA3	olfr421	PDGFD	PDGFRB
P4HB	olfr424	PGLYRP4	PGLYRP3
PAEP	olfr426	PGLYRP3	PGLYRP4
PAM	olfr427	HMGB1	PGR
Pamr1	olfr429	HMGB2	PGR
PAPL	olfr43	HSP90AA1	PGR

Papln	olfr430	CALR	PGRMC1
Pappa	olfr432	P4HB	PGRMC1
PAPPA2	olfr433	TIMP2	PGRMC1
PATE1	olfr434	COPA	PHB2
Pate2	olfr435	FLRT3	PHB2
PATE3	olfr437	GRIN2B	PHB2
Pate4	olfr44	LGALS3BP	PHB2
Pbsn	olfr441	YBX1	PHB2
Pcdh15	olfr444	FCAR	PILRA
PCDHA1	olfr446	IL6ST	PLAUR
PCDHA10	olfr447	KNG1	PLAUR
PCDHA6	olfr448	MMP12	PLAUR
Pcolce	olfr449	PLAU	PLAUR
Pcolce2	olfr45	SERPINE1	PLAUR
Pcsk1	olfr450	VTN	PLAUR
Pcsk1n	olfr452	NRP1	PLXNA1
Pcsk2	olfr453	PLXNB1	PLXNA1
Pcsk5	olfr455	MET	PLXNB1
Pcsk6	olfr456	PTN	PLXNB2
Pcsk9	olfr457	SPOCK2	PLXNB3
Pcyox1	olfr458	NRP1	PLXND1
Pcyox1l	olfr459	SEMA3E	PLXND1
Pdap1	olfr46	HSP90AA1	PPARA
PDCD1LG2	olfr460	PEBP1	PPARD
Pddc1	olfr461	NPY	PPYR1
Pdgfa	olfr462	PPY	PPYR1
Pdgfb	olfr463	PYY	PPYR1
Pdgfc	olfr464	PRLH	PRLHR
Pdgfd	olfr466	GH1	PRLR
Pdgfrl	olfr467	PPIA	PRLR
PDYN	olfr469	PRL	PRLR
PDZD11	olfr47	PROC	PROCR
Pdzd2	olfr470	DHH	PTCH1
Pebp1	olfr471	IHH	PTCH1
Pecam1	olfr472	SHH	PTCH1
Penk	olfr473	DHH	PTCH2
Pf4	olfr474	IHH	PTCH2
PF4V1	olfr476	SHH	PTCH2
PFN1	olfr477	CA2	PTCHD2
PGA4	olfr478	GNAS	PTGDR
Pgc	olfr479	PTGDS	PTGER1
Pgcp	olfr48	PTGFR	PTGER1
Pgf	olfr480	PTGDS	PTGER2
PGLYRP1	olfr481	PTGFR	PTGER2
PGLYRP2	olfr482	PTGDS	PTGFR
PGLYRP3	olfr483	GNAS	PTGIR
PGLYRP4	olfr484	PTH	PTH1R

Pi15	olfr485	PTHLH	PTH1R
Pi16	olfr486	PTH	PTH2R
PI3	olfr487	CHL1	PTPRA
Pigr	olfr488	GHR	PTPRB
PILRA	olfr49	LGALS1	PTPRB
Pip	olfr490	MET	PTPRB
PKHD1L1	olfr491	TNC	PTPRB
Pla1a	olfr492	CD2	PTPRC
Pla2g10	olfr493	CD8A	PTPRC
Pla2g12a	olfr494	DPP4	PTPRC
PLA2G12B	olfr495	FCGR3A	PTPRC
Pla2g15	olfr497	LGALS1	PTPRC
Pla2g1b	olfr498	LGALS3	PTPRC
Pla2g2a	olfr5	MBL2	PTPRC
Pla2g2c	olfr50	PAEP	PTPRC
Pla2g2d	olfr502	CD8A	PTPRCAP
Pla2g2e	olfr503	TLN1	PTPRE
Pla2g2f	olfr504	GALNT1	PTPRF
PLA2G3	olfr506	GRIA3	PTPRF
PLA2G4B	olfr507	LAMA1	PTPRF
Pla2g5	olfr508	LAMB1	PTPRF
Pla2g7	olfr509	LAMC1	PTPRF
PLA2R1	olfr51	NID1	PTPRF
Plac1	olfr510	GHR	PTPRH
Plac1l	olfr512	EGFR	PTPRJ
PLAC9	olfr513	MET	PTPRJ
Plat	olfr514	PCSK5	PTPRM
Plau	olfr516	PPIA	PTPRN
PLAUR	olfr517	KIT	PTPRO
Plbd1	olfr518	EGFR	PTPRS
PLEK	olfr519	PTN	PTPRS
Plg	olfr52	RAB35	PTPRS
PLGLA	olfr520	KIT	PTPRU
PLGLB2	olfr521	HMGB1	PTPRZ1
PLIN2	olfr522	MDK	PTPRZ1
Plod1	olfr523	PTN	PTPRZ1
PLOD2	olfr524	TNR	PTPRZ1
PLSCR1	olfr525	PVRL1	PVR
Pltp	olfr527	VTN	PVR
PLXDC1	olfr53	PVR	PVRL1
PLXNB1	olfr530	PVRL4	PVRL1
Pm20d1	olfr531	PVRL1	PVRL4
Pmch	olfr532	CALCA	RAMP1
PMCHL1	olfr533	IAPP	RAMP1
PMEL	olfr535	ADM	RAMP2
Pnlip	olfr536	CALCA	RAMP2
Pnliprp1	olfr538	IAPP	RAMP2

Pnliprp2	olfr539	RAMP1	RAMP2
PNLIPRP3	olfr54	ADM	RAMP3
Pnoc	olfr541	GDNF	RET
Pnp	olfr543	GFRA4	RET
Podn	olfr544	NRTN	RET
Podnl1	olfr545	PSPN	RET
Pomc	olfr547	SLIT1	ROBO1
Pon1	olfr549	SLIT2	ROBO1
Pon2	olfr55	CPE	ROBO2
Pon3	olfr550	OLFM2	ROBO2
Postn	olfr551	SLIT2	ROBO2
POTEKP	olfr552	SLIT3	ROBO2
PPBP	olfr553	APOA1	ROBO3
PPBPL2	olfr554	SLIT2	ROBO4
PPIA	olfr555	NGF	ROR1
Ppt1	olfr556	WNT5A	ROR1
Ppy	olfr557	WNT5A	ROR2
PRADC1	olfr558	IL17A	RORC
Prap1	olfr559	EGFR	ROS1
PRB1	olfr56	NGFR	RTN4R
PRB2	olfr560	RLN1	RXFP1
PRB3	olfr561	RLN2	RXFP1
PRB4	olfr564	RLN3	RXFP1
Prdx4	olfr566	INSL3	RXFP2
Prelp	olfr568	RLN2	RXFP2
Prf1	olfr569	KNG1	RXFP4
PRG2	olfr57	RLN3	RXFP4
PRG4	olfr570	CCL6	RXRA
PRH2	olfr571	CCL9	RXRA
Prl	olfr572	CTSL1	RXRA
Prl2a1	olfr574	IGFBP3	RXRA
Prl2b1	olfr575	CHGB	RXRG
Prl2c2	olfr576	LRSAM1	RXRG
Prl2c3	olfr577	WNT1	RYK
Prl2c5	olfr578	WNT3A	RYK
Prl3b1	olfr58	ADAM23	RYR1
Prl3c1	olfr582	PTN	RYR1
Prl3d1	olfr583	TF	RYR1
Prl4a1	olfr584	ALB	RYR2
Prl5a1	olfr585	RS1	SARM1
Prl6a1	olfr586	APOA1	SCARB1
Prl7a1	olfr589	APOE	SCARB1
Prl7a2	olfr59	THBS1	SCARB2
Prl7b1	olfr591	CALR	SCARF1
Prl7c1	olfr592	ADCYAP1	SCTR
Prl7d1	olfr593	CPAMD8	SCTR
Prl8a6	olfr594	SCT	SCTR

Prl8a8	olfr596	CCL5	SDC4
Prl8a9	olfr597	FGF2	SDC4
PRLH	olfr598	MDK	SDC4
PRLR	olfr599	ACTN2	SELE
PRNT	olfr6	FLNA	SELE
PRO2829	olfr60	GLG1	SELE
Proc	olfr600	SERPING1	SELE
Prok1	olfr601	VCL	SELE
Prok2	olfr603	SAA1	SELS
PROL1	olfr605	NRP1	SEMA3A
Prom1	olfr606	SEMA3B	SEMA3A
Prom2	olfr607	NRP1	SEMA3B
Pros1	olfr608	SEMA3A	SEMA3B
Proz	olfr609	NRP1	SEMA3C
PRR4	olfr61	NRP1	SEMA3D
PRRG1	olfr610	NRP1	SEMA3F
Prrg2	olfr611	SHBG	SEMA3F
Prrg3	olfr612	VEGFA	SEMA3F
Prrg4	olfr613	PLXNB1	SEMA4A
Prss1	olfr615	PLXNB1	SEMA4B
PRSS12	olfr616	PLXNB1	SEMA4D
Prss2	olfr617	TLR9	SIGIRR
PRSS22	olfr618	ALB	SLC1A5
Prss23	olfr619	LGALS9	SLC1A5
Prss27	olfr62	TNFRSF1A	SLC1A5
Prss28	olfr620	TNFRSF1B	SLC1A5
Prss29	olfr622	LCN2	SLC22A17
PRSS29P	olfr623	CSF1	SLC7A1
PRSS3	olfr624	FURIN	SORCS1
Prss33	olfr628	NGF	SORCS3
Prss34	olfr629	FURIN	SORL1
Prss35	olfr63	BDNF	SORT1
PRSS36	olfr630	NGF	SORT1
Prss37	olfr631	NGFR	SORT1
Prss38	olfr632	NTS	SORT1
Prss39	olfr633	ICAM1	SPN
Prss40	olfr635	LGALS1	SPN
Prss42	olfr638	LGALS3	SPN
Prss45	olfr639	SIGLEC1	SPN
PRSS47	olfr64	MET	SPSB1
Prss48	olfr640	C1QBP	SSR4
Prss53	olfr641	HSPD1	SSR4
Prss54	olfr642	SOD1	SSR4
Prss57	olfr643	TNFRSF1A	SSR4
Prss58	olfr644	TNFRSF1B	SSR4
Prss8	olfr645	CORT	SSTR1
PRTN3	olfr646	SST	SSTR1

Psap	olfr648	CORT	SSTR2
Psapl1	olfr649	SST	SSTR2
PSG1	olfr65	CORT	SSTR3
PSG11	olfr651	SST	SSTR3
PSG2	olfr652	CORT	SSTR4
PSG3	olfr653	SST	SSTR4
PSG4	olfr654	CORT	SSTR5
PSG5	olfr655	SST	SSTR5
PSG6	olfr656	CHID1	STAB1
PSG7	olfr657	SPARC	STAB1
PSG8	olfr658	TMSB4X	STAB2
PSG9	olfr659	GH1	STRAP
Psors1c2	olfr66	LGALS1	SUSD2
Pspn	olfr661	TAC1	TACR1
Ptgds	olfr663	TAC3	TACR1
PTGFR	olfr664	TAC1	TACR2
Ptgis	olfr665	TAC3	TACR2
Pth	olfr666	TAC1	TACR3
Pth2	olfr667	TAC3	TACR3
Pthlh	olfr668	ANGPT1	TEK
Ptn	olfr669	ANGPT2	TEK
Ptprg	olfr67	ANGPT4	TEK
PTPRZ1	olfr670	ANGPTL1	TEK
Ptx3	olfr671	TF	TFR2
PTX4	olfr672	B2M	TFRC
PVR	olfr675	LGALS3	TFRC
PVRL1	olfr676	TF	TFRC
PVRL4	olfr677	TLN1	TFRC
Pxdn	olfr678	VCL	TFRC
PXDNL	olfr679	CLU	TGFBR1
Pycard	olfr68	ENG	TGFBR1
Pyy	olfr681	GDF11	TGFBR1
PYY2	olfr683	MYOC	TGFBR1
PYY3	olfr684	PLEK	TGFBR1
Pzp	olfr685	TGFB1	TGFBR1
Q6ZRU5	olfr686	TGFB2	TGFBR1
Q6ZVS6	olfr687	TGFB3	TGFBR1
Qpct	olfr688	WDR33	TGFBR1
Qrfp	olfr689	CLU	TGFBR2
Qsox1	olfr69	ENG	TGFBR2
QSOX2	olfr690	SPARC	TGFBR2
R3hdml	olfr691	TGFB1	TGFBR2
Rab35	olfr692	TGFB1I1	TGFBR2
RAMP1	olfr693	TGFB2	TGFBR2
Rarres2	olfr694	TGFB3	TGFBR2
Rbmx	olfr695	TGFBR3	TGFBR2
Rbp3	olfr697	ENG	TGFBR3

Rbp4	olfr698	INHA	TGFBR3
Reg1	olfr699	INHBA	TGFBR3
REG1A	olfr70	TGFB1	TGFBR3
REG1B	olfr700	TGFB2	TGFBR3
Reg2	olfr701	TGFB3	TGFBR3
Reg3a	olfr702	F2	THBD
Reg3b	olfr703	PF4	THBD
Reg3g	olfr704	PROC	THBD
Reg4	olfr705	SERPINA5	THBD
Rell2	olfr706	SERPINE1	THBD
Reln	olfr707	VTN	THBD
REN	olfr71	ALB	THRAP3
Ren1	olfr710	CLEC3B	THRAP3
Ren2	olfr711	ANGPT4	TIE1
Resp18	olfr713	CASP1	TIRAP
Retn	olfr714	IL1RL1	TIRAP
Retnla	olfr715	FLNA	TLR1
Retnlb	olfr716	HSPD1	TLR1
RFNG	olfr720	TLR9	TLR1
RLN1	olfr722	FLNA	TLR10
RLN2	olfr723	CD14	TLR2
Rln3	olfr724	EGFR	TLR2
RNASE1	olfr725	HMGB1	TLR2
Rnase10	olfr726	LY96	TLR2
RNASE11	olfr727	SFTPA1	TLR2
Rnase12	olfr728	SFTPD	TLR2
Rnase13	olfr729	CD14	TLR3
RNASE2	olfr73	ANXA2	TLR4
RNASE3	olfr730	CD14	TLR4
RNASE4	olfr731	CNPY4	TLR4
RNASE6	olfr732	EGFR	TLR4
RNASE7	olfr733	HMGB1	TLR4
RNASE8	olfr734	LY86	TLR4
Rnase9	olfr735	LY96	TLR4
RNASET2	olfr736	MBL2	TLR4
Rnh1	olfr738	S100A8	TLR4
Rnls	olfr739	SFTPA1	TLR4
Rnpep	olfr74	SFTPA2	TLR4
RPESP	olfr740	SFTPD	TLR4
RPTN	olfr741	TREM1	TLR4
Rs1	olfr742	CD14	TLR7
Rspo1	olfr743	CD14	TLR8
Rspo2	olfr744	TLR9	TLR8
Rspo3	olfr745	AGER	TLR9
Rspo4	olfr746	CD14	TLR9
Rspry1	olfr747	HMGB1	TLR9
Rtbdn	olfr748	APP	TM2D1

RTN3	olfr749	SPINK1	TMPRSS15
S100A12	olfr750	COL2A1	TNFRSF10A
S100a13	olfr76	TNFSF10	TNFRSF10A
S100A7	olfr761	FAS	TNFRSF10B
S100a8	olfr763	FASLG	TNFRSF10B
S100a9	olfr764	TNFRSF1A	TNFRSF10B
S100b	olfr765	TNFSF10	TNFRSF10B
Saa1	olfr766	APOA1	TNFRSF10C
Saa2	olfr767	LRSAM1	TNFRSF10C
Saa3	olfr768	TNFSF10	TNFRSF10C
SAA3P	olfr769	CALR	TNFRSF10D
Saa4	olfr77	MIF	TNFRSF10D
Saal1	olfr770	TNFSF10	TNFRSF10D
SAMD1	olfr771	WDR1	TNFRSF10D
Sbp	olfr772	TNFSF11	TNFRSF11A
Sbsn	olfr773	FN1	TNFRSF11B
Scara3	olfr774	THBS1	TNFRSF11B
Scg2	olfr775	TNFSF10	TNFRSF11B
Scg3	olfr776	TNFSF11	TNFRSF11B
Scg5	olfr777	TNFSF13	TNFRSF11B
Scgb1a1	olfr779	VTN	TNFRSF11B
SCGB1C1	olfr78	VWF	TNFRSF11B
SCGB1D1	olfr780	TNFSF12	TNFRSF12A
SCGB1D2	olfr781	TNFSF13	TNFRSF13B
SCGB1D4	olfr782	TNFSF13B	TNFRSF13B
SCGB2A1	olfr784	TNFSF13B	TNFRSF13C
SCGB2A2	olfr786	DCD	TNFRSF14
Scgb3a1	olfr787	LTA	TNFRSF14
Scgb3a2	olfr788	MIF	TNFRSF14
SCGBL	olfr790	P4HB	TNFRSF14
Scgn	olfr791	TNFSF13	TNFRSF14
Scpep1	olfr792	TNFSF14	TNFRSF14
Scrg1	olfr794	TNFSF13	TNFRSF17
Sct	olfr796	TNFSF13B	TNFRSF17
Scube1	olfr798	TNFSF18	TNFRSF18
Scube2	olfr799	C1QBP	TNFRSF1A
Scube3	olfr8	ERAP1	TNFRSF1A
Sdc1	olfr800	FAS	TNFRSF1A
SDC4	olfr801	FASLG	TNFRSF1A
Sdf2	olfr802	FBLN2	TNFRSF1A
SECTM1	olfr803	HSP90AA1	TNFRSF1A
SELE	olfr804	HSPA5	TNFRSF1A
Selp	olfr805	HSPA8	TNFRSF1A
SELS	olfr806	KARS	TNFRSF1A
SEMA3A	olfr807	LTA	TNFRSF1A
SEMA3B	olfr808	LTB	TNFRSF1A
SEMA3C	olfr809	MYOC	TNFRSF1A

SEMA3D	olfr810	SLC1A5	TNFRSF1A
SEMA3E	olfr811	TNF	TNFRSF1A
SEMA3F	olfr812	TNFRSF25	TNFRSF1A
SEMA3G	olfr813	TNFSF13	TNFRSF1A
SEMG1	olfr814	C1QBP	TNFRSF1B
SEMG2	olfr815	FLNA	TNFRSF1B
SEPN1	olfr816	HSPA5	TNFRSF1B
Sepp1	olfr818	HSPA8	TNFRSF1B
Serac1	olfr819	LTA	TNFRSF1B
SERPINA1	olfr820	POTEKP	TNFRSF1B
Serpina10	olfr821	SERPINB3	TNFRSF1B
Serpina11	olfr822	SLC1A3	TNFRSF1B
Serpina12	olfr823	SLC1A5	TNFRSF1B
SERPINA13	olfr824	TNF	TNFRSF1B
Serpina1a	olfr825	TNFSF10	TNFRSF22
Serpina1b	olfr826	TNFSF10	TNFRSF23
Serpina1c	olfr827	TNFRSF1A	TNFRSF25
Serpina1d	olfr828	TNFSF12	TNFRSF25
Serpina1e	olfr829	TNFSF15	TNFRSF25
Serpina1f	olfr830	TNFSF4	TNFRSF4
SERPINA2	olfr832	FASLG	TNFRSF6B
SERPINA3	olfr834	TNFSF14	TNFRSF6B
Serpina3a	olfr835	TNFSF15	TNFRSF6B
Serpina3b	olfr836	TNFSF8	TNFRSF8
Serpina3c	olfr837	TNFSF9	TNFRSF9
Serpina3f	olfr843	ADAM17	TNFSF11
Serpina3g	olfr844	MMP1	TNFSF11
Serpina3h	olfr845	MMP3	TNFSF11
Serpina3k	olfr846	MMP7	TNFSF11
Serpina3m	olfr847	TNFRSF11B	TNFSF11
Serpina3n	olfr849	TNFRSF18	TNFSF18
SERPINA4	olfr850	TRH	TRHR
Serpina5	olfr851	TRH	TRHR2
Serpina6	olfr853	ANXA1	TRPM7
Serpina7	olfr854	ANXA2	TRPV6
Serpina9	olfr855	CALR	TSHR
SERPINB1	olfr857	FN1	TSHR
Serpinb13	olfr859	GNAS	TSHR
Serpinb1a	olfr860	HSPA5	TSHR
Serpinb1b	olfr862	LHCGR	TSHR
Serpinb1c	olfr866	TSHB	TSHR
Serpinb2	olfr867	GAS6	TYRO3
SERPINB3	olfr868	PROS1	TYRO3
Serpinb3a	olfr869	STX1A	UNC13B
Serpinb3b	olfr870	NTN1	UNC5A
Serpinb3c	olfr871	NTN1	UNC5B
Serpinb3d	olfr872	NTN1	UNC5C

SERPINB4	olfr873	UTS2	UTS2R
Serpinb5	olfr874	UTS2D	UTS2R
Serpinb9	olfr875	CAMP	VDR
Serpinc1	olfr876	ADCYAP1	VIPR1
SERPIND1	olfr877	GNAS	VIPR1
Serpine1	olfr878	PFN1	VIPR1
Serpine2	olfr881	RAMP1	VIPR1
SERPINE3	olfr883	SCT	VIPR1
Serpinf1	olfr884	VIP	VIPR1
Serpinf2	olfr885	ADCYAP1	VIPR2
Serping1	olfr887	ERBB2IP	VIPR2
SERPINH1	olfr888	VIP	VIPR2
Serpini1	olfr889	APOE	VLDLR
Serpini2	olfr890	CLU	VLDLR
Sez6	olfr891	LPL	VLDLR
Sfn	olfr893	PLAU	VLDLR
SFRP1	olfr894	RELN	VLDLR
SFRP2	olfr895	SERPINE1	VLDLR
SFRP4	olfr898	XCL1	XCR1
SFRP5	olfr899	XCL2	XCR1
SFTA2	olfr9	BMP4	ZFYVE9
Sftpa1	olfr90	ERBB2IP	ZFYVE9
SFTPA2	olfr900	RNPEP	ZFYVE9
Sftpb	olfr901	SVEP1	ZFYVE9
Sftpc	olfr902	ZPBP	ZP2
Sftpd	olfr904	FURIN	ZP3
Shbg	olfr905	ZP3	ZP3R
Shh	olfr906	FURIN	ZP4
SIAE	olfr907		
Siglec1	olfr908		
SIGLEC10	olfr91		
SIGLEC6	olfr910		
SIRPD	olfr912		
Slc1a3	olfr913		
Slc1a5	olfr914		
Slc2a4	olfr915		
Slit1	olfr916		
Slit2	olfr917		
Slit3	olfr918		
Slmap	olfr919		
Slpi	olfr92		
Slurp1	olfr920		
SLURP2	olfr921		
Smc3	olfr922		
Smgc	olfr923		
Smoc1	olfr924		
Smoc2	olfr926		

Smpd1	olfr93
Smpdl3a	olfr930
Smpdl3b	olfr933
Smr2	olfr934
Smr3a	olfr935
SMR3B	olfr936
Snca	olfr937
Sned1	olfr938
Sod1	olfr94
Sod3	olfr943
SORD	olfr944
SORL1	olfr945
Sost	olfr948
Sostdc1	olfr95
Spaca3	olfr951
Spaca5	olfr952
Spaca7	olfr954
SPAG11A	olfr955
SPAG11B	olfr957
Sparc	olfr958
Sparcl1	olfr959
Spata20	olfr96
Spata6	olfr960
SPINK1	olfr961
Spink11	olfr963
Spink12	olfr965
Spink13	olfr967
SPINK14	olfr968
Spink2	olfr969
Spink3	olfr97
SPINK4	olfr970
Spink5	olfr971
Spink6	olfr972
SPINK7	olfr974
Spink8	olfr975
SPINK9	olfr976
Spinkl	olfr978
Spinlw1	olfr979
Spint1	olfr98
SPINT2	olfr980
SPINT3	olfr981
Spint4	olfr982
Spn	olfr983
Spock1	olfr984
Spock2	olfr985
Spock3	olfr986
Spon1	olfr987

Spon2	olfr988
Spp1	olfr99
Spp2	olfr992
SRCRB4D	olfr993
Srgn	olfr994
Srpx2	olfr995
SSC5D	olfr996
Sspo	olfr998
Sst	olr1
St14	opcml
St3gal1	opn1lw
St3gal2	opn1mw
ST3GAL3	opn1mw2
ST3GAL4	opn1sw
St6gal1	opn3
STATH	opn4
Stc1	opn5
Stc2	oprd1
STRCP1	oprk1
STX1A	oprl1
Sulf1	oprm1
Sulf2	oprs1
Sva	or10a2
Svep1	or10a3
Svs3a	or10a4
Svs4	or10a5
Svs5	or10a6
Tac1	or10a7
Tac2	or10ac1p
TAC3	or10ad1
Tac4	or10ag1
Tbc1d15	or10c1
TCN1	or10d3
Tcn2	or10d4p
Tctn1	or10g2
Tdgf1	or10g3
Tecta	or10g4
Tectb	or10g6
TEPP	or10g7
TEX264	or10g8
TF	or10g9
Tff1	or10h1
TFF2	or10h2
Tff3	or10h3
Tfip11	or10h4
Tfpi	or10h5
Tfpi2	or10j1

TFRC	or10j3
Tg	or10j4
Tgfa	or10j5
Tgfb1	or10j6p
TGFB1I1	or10k1
Tgfb2	or10k2
Tgfb3	or10p1
Tgfbi	or10q1
TGFBR3	or10r2
Tgm2	or10s1
Tgm4	or10t2
THBD	or10v1
Thbs1	or10w1
Thbs2	or10x1
Thbs3	or10z1
Thbs4	or11a1
THNSL2	or11g2
Thpo	or11h1
THSD1	or11h12
Thsd4	or11h2
Timp1	or11h4
Timp2	or11h6
Timp3	or11h7
Timp4	or11l1
Tinag	or12d2
TINAGL1	or12d3
Tll1	or13a1
Tll2	or13c2
TLN1	or13c3
TLR9	or13c4
TMEFF2	or13c5
TMEM155	or13c8
TMEM229A	or13c9
Tmem25	or13d1
Tmprss11a	or13f1
TMPRSS11B	or13g1
Tmprss11bnl	or13h1
Tmprss11d	or13j1
Tmprss11e	or14a16
Tmprss11f	or14a2
TMPRSS2	or14c36
TMSB4X	or14i1
Tnc	or14j1
Tnf	or14k1
TNFAIP2	or14l1p
TNFRSF11B	or1a1
TNFRSF18	or1a2

TNFRSF1A	or1b1
TNFRSF1B	or1c1
TNFRSF25	or1d2
TNFRSF6B	or1d4
Tnfsf10	or1d5
TNFSF11	or1e1
Tnfsf12	or1e2
Tnfsf13	or1e3
Tnfsf13b	or1f1
Tnfsf14	or1f12
Tnfsf15	or1f2p
TNFSF18	or1g1
Tnfsf4	or1i1
TNFSF5	or1j1
Tnfsf8	or1j2
Tnfsf9	or1j4
Tnn	or1k1
Tnr	or1l1
Tnxb	or1l3
Tor2a	or1l4
TP73-AS1	or1l6
TPSAB1	or1l8
Tpsb2	or1m1
TPSD1	or1n1
Tpt1	or1n2
TREM1	or1p1
TREM2	or1q1
TREML4	or1s1
Trf	or1s2
Trh	or2a1
TRY6	or2a12
TSHB	or2a14
Tsku	or2a2
Tslp	or2a25
TSPEAR	or2a4
TTN	or2a5
Ttr	or2a7
TUB	or2ae1
TUBA4A	or2ag1
Tuft1	or2ag2
TULP1	or2aj1
Tulp2	or2ak2
Tulp3	or2ap1
Twsg1	or2at4
TXLNA	or2b11
TXN	or2b2
Txn1	or2b3

Txndc16	or2b6
UACA	or2b8p
Ucma	or2c1
Ucn	or2c3
Ucn2	or2d2
Ucn3	or2d3
ULBP2	or2f1
Umod	or2f2
Umodl1	or2g2
Ush2a	or2g3
Utp11l	or2g6
Uts2	or2h1
Uts2d	or2h2
Vash1	or2i1p
VASN	or2j1
Vcam1	or2j2
Vcan	or2j3
VCL	or2k2
Vegfa	or2l13
Vegfb	or2l2
Vegfc	or2l3
Vgf	or2l5
Vip	or2l8
Vit	or2m2
VLDLR	or2m3
Vmo1	or2m4
Vnn1	or2m5
Vnn3	or2m7
VPREB1	or2s2
VSIG6	or2t1
VSIG7	or2t10
Vstm2a	or2t11
VTN	or2t12
Vwa1	or2t2
Vwa2	or2t27
VWA3A	or2t29
Vwa5b1	or2t3
Vwc2	or2t33
Vwc2l	or2t34
Vwce	or2t35
Vwde	or2t4
Vwf	or2t5
Wap	or2t6
WDR1	or2t7
WDR33	or2t8
Wfdc1	or2v1
WFDC10A	or2v2

WFDC10B	or2w1
WFDC11	or2w3
Wfdc12	or2w5
WFDC13	or2w6p
Wfdc15a	or2y1
Wfdc15b	or2z1
Wfdc2	or3a1
WFDC3	or3a2
WFDC5	or3a3
WFDC6	or3a4
Wfdc8	or4a15
Wfdc9	or4a16
Wfikkn1	or4a47
Wfikkn2	or4a4p
Wif1	or4a5
Wisp1	or4a8p
Wisp2	or4b1
WISP3	or4c11
Wnt1	or4c12
Wnt10a	or4c13
Wnt10b	or4c15
Wnt11	or4c16
Wnt16	or4c3
Wnt2	or4c45
Wnt2b	or4c46
Wnt3	or4c5
Wnt3a	or4c6
Wnt4	or4d1
Wnt5a	or4d10
Wnt5b	or4d11
Wnt6	or4d2
Wnt7a	or4d5
Wnt7b	or4d6
Wnt8a	or4d9
Wnt8b	or4e1
Wnt9a	or4e2
Wnt9b	or4f15
Xcl1	or4f17
XCL2	or4f21
Xdh	or4f3
XYLT1	or4f4
YARS	or4f5
Ybx1	or4f6
Zg16	or4k1
ZG16B	or4k13
Zp1	or4k14
ZP2	or4k15

ZP3	or4k17
ZP4	or4k2
Zpbp	or4k3
Zpbp2	or4k5
	or4l1
	or4m1
	or4m2
	or4n2
	or4n4
	or4n5
	or4p4
	or4q2
	or4q3
	or4s1
	or4s2
	or4x1
	or4x2
	or51a2
	or51a4
	or51a7
	or51b2
	or51b4
	or51b5
	or51b6
	or51d1
	or51e1
	or51e2
	or51f1
	or51f2
	or51g1
	or51g2
	or51h1p
	or51i1
	or51i2
	or51j1
	or51l1
	or51m1
	or51q1
	or51s1
	or51t1
	or51v1
	or52a1
	or52a4
	or52a5
	or52b2
	or52b4
	or52b6

or52d1
or52e1
or52e2
or52e4
or52e5
or52e6
or52e8
or52h1
or52i1
or52i2
or52j3
or52k1
or52k2
or52l1
or52l2p
or52m1
or52n1
or52n2
or52n4
or52n5
or52p1p
or52r1
or52w1
or52z1
or56a1
or56a3
or56a4
or56a5
or56b1
or56b2p
or56b4
or5a1
or5a2
or5ac1
or5ac2
or5ak2
or5ak3p
or5al1
or5an1
or5ap2
or5ar1
or5as1
or5au1
or5b12
or5b17
or5b2
or5b21

or5b3
or5c1
or5d13
or5d14
or5d16
or5d18
or5f1
or5g3
or5h1
or5h14
or5h15
or5h2
or5h6
or5i1
or5j2
or5k1
or5k2
or5k3
or5k4
or5l1
or5l2
or5m1
or5m10
or5m11
or5m3
or5m8
or5m9
or5p2
or5p3
or5r1
or5t1
or5t2
or5t3
or5v1
or5w2
or6a2
or6b1
or6b2
or6b3
or6c1
or6c2
or6c3
or6c4
or6c6
or6c65
or6c68
or6c70

or6c74	
or6c75	
or6c76	
or6f1	
or6j1	
or6k2	
or6k3	
or6k6	
or6m1	
or6n1	
or6n2	
or6p1	
or6q1	
or6s1	
or6t1	
or6v1	
or6x1	
or6y1	
or7a10	
or7a17	
or7a2p	
or7a5	
or7c1	
or7c2	
or7d2	
or7d4	
or7e24	
or7g1	
or7g2	
or7g3	
or8a1	
or8b12	
or8b2	
or8b3	
or8b4	
or8b8	
or8d1	
or8d2	
or8d4	
or8g1	
or8g2	
or8g5	
or8h1	
or8h2	
or8h3	
or8i2	
or8j1	

or8j2
or8j3
or8k1
or8k3
or8k5
or8s1
or8u1
or8u8
or8u9
or9a1p
or9a2
or9a4
or9g1
or9g4
or9g9
or9i1
or9k2
or9q1
or9q2
oscar
osmr
oxer1
oxgr1
oxtr
p01737
p04436
p2rx1
p2rx2
p2rx3
p2rx4
p2rx5
p2rx6
p2rx7
p2ry1
p2ry10
p2ry11
p2ry12
p2ry13
p2ry14
p2ry2
p2ry4
p2ry6
p2ry8
paqr3
paqr4
paqr5
paqr6

paqr7
paqr8
paqr9
pax8
pdcd1lg2
pdgfra
pdgfrb
pdgfrl
pear1
pex5
pgbd1
pglyrp1
pglyrp2
pglyrp3
pglyrp4
pgr
pgr15l
pgrmc1
pgrmc2
phb2
pilra
pilrb
pira11
pkhd1
pkhd1l1
pla2r1
plaur
plxna1
plxna2
plxna3
plxna4
plxnb1
plxnb2
plxnb3
plxnc1
plxnd1
pnrc1
ppara
ppard
pparg
ppargc1a
ppargc1b
ppyr1
pr
prg4
prlhr
prlr

procr
prokr1
prokr2
prss12
ptafr
ptch1
ptch2
ptchd1
ptchd2
ptchd3
ptcra
ptgdr
ptger1
ptger2
ptger3
ptger4
ptgfr
ptgir
pth1r
pth2r
ptk7
ptpn1
ptpn12
ptpn14
ptpn18
ptpn1l
ptpn2
ptpn20a
ptpn23
ptpn3
ptpn5
ptpn7
ptpn9
ptpra
ptprb
ptprc
ptprcap
ptprd
ptpre
ptprf
ptprg
ptprh
ptprj
ptprk
ptprm
ptprn
ptprn2

ptpro
ptprq
ptprr
ptprs
ptprt
ptpru
ptprz1
pvr
pvrl1
pvrl2
pvrl4
q32vq0
q4g0t1
q6iex5
q8n0w0
q8nga4
q8ngm4
q8ngm6
q8nh33
q8nh68
q8nh95
qrfpr
rage
ramp1
ramp2
ramp3
rapsn
rara
rarb
rarg
reep1
reep2
reep5
reep6
relt
ret
rgr
rho
ripk2
ripk4
rnf135
rnf139
robo1
robo2
robo3
robo4
ror1

ror2
rora
rorb
rorc
ros1
rpesp
rpsa
rrbp1
rrh
rtn4r
rtn4rl1
rtn4rl2
rtp1
rxfp1
rxfp2
rxfp3
rxfp4
rxra
rxrb
rxrg
ryk
ryr1
ryr2
ryr3
s1pr1
s1pr2
s1pr3
s1pr4
s1pr5
sarg
sarm1
scara3
scara5
scarb1
scarb2
scarf1
scarf2
sctr
sdc4
sec62
sec63
sele
sels
sema3a
sema3b
sema3c
sema3d

sema3e
sema3f
sema3g
sema4a
sema4b
sema4c
sema4d
sema4f
sema4g
sema5a
sema5b
sema6a
sema6b
sema6c
sema6d
sema7a
sfrp1
sfrp2
sfrp4
sfrp5
sigirr
siglec7
siglec8
siglece
sigmar1
siva1
slamf1
slamf6
slamf7
slamf8
slamf9
slc1a5
slc20a1
slc20a2
slc22a17
slc7a1
smo
sorcs1
sorcs2
sorcs3
sorl1
sort1
specc1l
spn
spsb1
spsb3
sra1

srcrb4d
srpr
srprb
ssc5d
ssr3
ssr4
sstr1
sstr2
sstr3
sstr4
sstr5
stab1
stab2
stra6
strap
sucnr1
susd2
sv2a
sv2b
sv2c
taar1
taar2
taar3
taar4
taar5
taar6
taar7a
taar7b
taar7d
taar7e
taar7f
taar8
taar8a
taar8b
taar8c
taar9
tacr1
tacr2
tacr3
tacstd2
tapt1
tas1r1
tas1r2
tas1r3
tas2r1
tas2r10
tas2r102

tas2r103
tas2r104
tas2r105
tas2r106
tas2r107
tas2r108
tas2r109
tas2r110
tas2r113
tas2r114
tas2r115
tas2r116
tas2r117
tas2r118
tas2r119
tas2r12
tas2r120
tas2r121
tas2r122
tas2r123
tas2r124
tas2r125
tas2r126
tas2r129
tas2r13
tas2r130
tas2r131
tas2r134
tas2r135
tas2r136
tas2r137
tas2r138
tas2r139
tas2r14
tas2r140
tas2r143
tas2r144
tas2r16
tas2r19
tas2r20
tas2r3
tas2r30
tas2r31
tas2r38
tas2r39
tas2r4
tas2r40

tas2r41
tas2r42
tas2r43
tas2r45
tas2r46
tas2r5
tas2r50
tas2r60
tas2r7
tas2r8
tas2r9
tbxa2r
tcra
tcrb
tek
tfr2
tfrc
tg
tgfbr1
tgfbr2
tgfbr3
thbd
thra
thrap3
thrb
ticam2
tie1
timd2
tinag
tinagl1
tirap
tirap3
tlr1
tlr10
tlr11
tlr12
tlr13
tlr2
tlr3
tlr4
tlr5
tlr6
tlr7
tlr8
tlr9
tm2d1
tmem123

tmem149
tmprss13
tmprss15
tmprss2
tmprss3
tmprss4
tmprss5
tnfrsf10a
tnfrsf10b
tnfrsf10c
tnfrsf10d
tnfrsf11a
tnfrsf11b
tnfrsf12a
tnfrsf13b
tnfrsf13c
tnfrsf14
tnfrsf17
tnfrsf18
tnfrsf19
tnfrsf1a
tnfrsf1b
tnfrsf21
tnfrsf22
tnfrsf23
tnfrsf25
tnfrsf26
tnfrsf4
tnfrsf6b
tnfrsf8
tnfrsf9
tnfsf11
tnfsf18
tnk1
tnk2
tnpo3
tomm22
tomm70a
tpra1
trac
traf1
traf2
traf4
tram1
trbc1
trbc2
trbv12-3

trbv20or9-2
trdc
trem1
trem2
trem3
treml1
treml2
treml4
trfr2
trgc1
trgc2
trgv3
trhr
trhr2
trim27
trip6
trpc1
trpc2
trpc3
trpc4
trpc4ap
trpc5
trpc7
trpm1
trpm2
trpm3
trpm4
trpm5
trpm6
trpm7
trpm8
trpv4
trpv6
tshr
tspan12
tspo
tspo2
tub
tulp1
tyro3
ulbp1
ulbp2
ulbp3
unc13b
unc13c
unc5a
unc5b

unc5c
unc5d
uts2r
v1ra8
v1rg10
v1rh19
vac14
vdr
vipr1
vipr2
vldlr
vmn1r10
vmn1r11
vmn1r112
vmn1r114
vmn1r115
vmn1r118
vmn1r120
vmn1r121
vmn1r123
vmn1r124
vmn1r127
vmn1r128
vmn1r13
vmn1r14
vmn1r142
vmn1r15
vmn1r16
vmn1r160
vmn1r17
vmn1r171
vmn1r172
vmn1r178
vmn1r179
vmn1r18
vmn1r180
vmn1r181
vmn1r183
vmn1r185
vmn1r188
vmn1r189
vmn1r19
vmn1r191
vmn1r192
vmn1r193
vmn1r195
vmn1r197

vmn1r198
vmn1r199
vmn1r2
vmn1r200
vmn1r201
vmn1r202
vmn1r203
vmn1r205
vmn1r206
vmn1r208
vmn1r209
vmn1r21
vmn1r210
vmn1r211
vmn1r212
vmn1r213
vmn1r214
vmn1r215
vmn1r216
vmn1r217
vmn1r218
vmn1r219
vmn1r22
vmn1r220
vmn1r222
vmn1r223
vmn1r225
vmn1r226
vmn1r227
vmn1r228
vmn1r229
vmn1r23
vmn1r230
vmn1r231
vmn1r232
vmn1r233
vmn1r234
vmn1r235
vmn1r236
vmn1r237
vmn1r24
vmn1r25
vmn1r26
vmn1r27
vmn1r28
vmn1r29
vmn1r3

vmn1r30	
vmn1r32	
vmn1r33	
vmn1r35	
vmn1r36	
vmn1r37	
vmn1r38	
vmn1r4	
vmn1r40	
vmn1r41	
vmn1r42	
vmn1r43	
vmn1r44	
vmn1r45	
vmn1r46	
vmn1r47	
vmn1r48	
vmn1r49	
vmn1r5	
vmn1r50	
vmn1r51	
vmn1r52	
vmn1r53	
vmn1r54	
vmn1r56	
vmn1r59	
vmn1r6	
vmn1r62	
vmn1r63	
vmn1r64	
vmn1r65	
vmn1r66	
vmn1r67	
vmn1r69	
vmn1r7	
vmn1r70	
vmn1r71	
vmn1r72	
vmn1r73	
vmn1r74	
vmn1r75	
vmn1r76	
vmn1r78	
vmn1r79	
vmn1r8	
vmn1r80	
vmn1r81	

vmn1r82	
vmn1r83	
vmn1r84	
vmn1r85	
vmn1r87	
vmn1r89	
vmn1r9	
vmn1r91	
vmn1r94	
vmn2r1	
vmn2r10	
vmn2r100	
vmn2r101	
vmn2r102	
vmn2r103	
vmn2r104	
vmn2r105	
vmn2r107	
vmn2r108	
vmn2r109	
vmn2r11	
vmn2r110	
vmn2r111	
vmn2r112	
vmn2r113	
vmn2r114	
vmn2r115	
vmn2r116	
vmn2r117	
vmn2r118	
vmn2r12	
vmn2r120	
vmn2r121	
vmn2r123	
vmn2r13	
vmn2r14	
vmn2r15	
vmn2r16	
vmn2r17	
vmn2r18	
vmn2r2	
vmn2r20	
vmn2r21	
vmn2r22	
vmn2r23	
vmn2r24	
vmn2r26	

vmn2r27
vmn2r28
vmn2r29
vmn2r3
vmn2r30
vmn2r31
vmn2r32
vmn2r33
vmn2r34
vmn2r35
vmn2r37
vmn2r38
vmn2r39
vmn2r4
vmn2r41
vmn2r42
vmn2r43
vmn2r44
vmn2r45
vmn2r48
vmn2r49
vmn2r5
vmn2r50
vmn2r51
vmn2r52
vmn2r54
vmn2r56
vmn2r57
vmn2r58
vmn2r59
vmn2r6
vmn2r60
vmn2r61
vmn2r62
vmn2r63
vmn2r66
vmn2r67
vmn2r7
vmn2r70
vmn2r71
vmn2r73
vmn2r74
vmn2r76
vmn2r77
vmn2r78
vmn2r79
vmn2r8

vmn2r80
vmn2r81
vmn2r83
vmn2r84
vmn2r87
vmn2r89
vmn2r9
vmn2r90
vmn2r91
vmn2r92
vmn2r93
vmn2r94
vmn2r95
vmn2r96
vmn2r97
vmn2r98
vmn2r99
vn1r1
vn1r17p
vn1r2
vn1r3
vn1r4
vn1r5
vpreb1
vtn
wls
xcr1
xpr1
zacn
zfyve9
znf219
zp2
zp3
zp3r
zp4

Supplementary Table S3

Secretory Molecules (Ligand)	Receptors	Interactions	
A2M	ACVR1	**Secretory**	**Receptor**
ADCYAP1	ACVR1B	A2M	LRP1
ADM	ACVR2A	ACE	AGTR2
AFP	ACVR2B	ADCYAP1	ADCYAP1R1
AGRP	ACVRL1	ADIPOQ	ADIPOR2
JAG1	ADCYAP1R1	ADM	CALCRL
AGT	ADRA1A	AFP	CCR5
ALB	ADRBK1	AGR3	DAG1
AMH	AGER	AGRN	DAG1
ANGPT1	AGTR1	AGRP	MC3R
ANGPT2	AGTR2	AGRP	MC4R
APOA1	APLNR	AGRP	MC5R
APOB	ALK	AGT	AGTR1
APOE	AMHR2	AGT	AGTR2
APOH	FAS	AGT	MAS1
APP	ASGR1	ALB	CUBN
FASLG	ASGR2	AMH	AMHR2
ASIP	AVPR1A	ANGPT1	ITGA5
AVP	AVPR1B	ANGPT1	TEK
BDNF	AVPR2	ANGPT2	TEK
CEACAM1	AXL	ANGPT4	TEK
BMP1	TNFRSF17	ANGPT4	TIE1
BMP2	CXCR5	ANGPTL1	TEK
BMP3	BMPR1A	ANGPTL3	ITGA5
BMP4	BMPR1B	ANGPTL3	ITGAV
BMP5	BMPR2	ANGPTL3	ITGB3
BMP6	BTN1A1	APLN	APLNR
BMP7	C3AR1	APOA1	SCARB1
BTC	C5AR1	APOB	LDLR
C1QA	DDR1	APOB	LRP2
C3	CCKAR	APOE	LDLR
C4A	CCKBR	APOE	LRP1
C5	CD2	APOE	LRP2
CALCA	CD7	APOE	LRP8
CALR	CD14	APOE	SCARB1
CCK	CD22	APOE	VLDLR
TNFSF8	TNFRSF8	APOH	LRP2
CD40LG	CD36	APOH	LRP8
CD59	SCARB1	APP	LRP1
CGA	CD40	APP	TM2D1
CHAD	CD44	ARTN	GFRA3
CHGB	CD47	ASIP	ATRN
CLU	CHRNA4	ASIP	MC2R
CNTF	CHRNB2	ASIP	MC3R

COL1A1	CCR1	ASIP	MC4R
COL1A2	CCR3	ASIP	MC5R
COL2A1	CCR4	AVP	AVPR1A
COL3A1	CCR5	AVP	AVPR1B
COL4A1	CCR6	AVP	AVPR2
COL4A2	CCR7	AVP	OXTR
COL4A3	CCR8	BDNF	NGFR
COL4A4	CCBP2	BDNF	NTRK2
COL4A5	CNTFR	BDNF	SORT1
COL4A6	CR2	BMP1	ADRA1A
COL5A2	CRHR1	BMP15	BMPR1B
COL6A1	CRHR2	BMP2	ACVR1
COL6A3	CSF1R	BMP2	ACVR2A
COL8A1	CSF2RA	BMP2	ACVR2B
COL11A1	CSF2RB	BMP2	BMPR1A
COL11A2	CSF3R	BMP2	BMPR1B
COL17A1	CX3CR1	BMP2	BMPR2
CORT	DAG1	BMP2	ENG
CRH	DCC	BMP3	ACVR2A
CSF1	DMBT1	BMP3	ACVR2B
CSF2	EDNRA	BMP4	BMPR1A
CSF3	EDNRB	BMP4	BMPR1B
CTF1	EGFR	BMP4	BMPR2
CTGF	EPHA2	BMP4	ZFYVE9
ACE	ENG	BMP5	BMPR1A
DPP4	EPHA1	BMP6	ACVR2A
DSCAM	EPHA3	BMP6	ACVR2B
HBEGF	EPHA4	BMP6	BMPR1A
EDA	EPHA5	BMP6	BMPR1B
EDN1	EPHA7	BMP6	BMPR2
EDN3	EPHA8	BMP7	ACVR1
EFNA1	EPHB1	BMP7	ACVR2A
EFNA4	EPOR	BMP7	ACVR2B
EGF	ERBB2	BMP7	BMPR1A
EPO	ERBB3	BMP7	BMPR1B
EREG	ERBB4	BMP7	BMPR2
F2	F2R	BMP7	ENG
F8	F2RL1	BTC	EGFR
F11	F2RL2	BTC	ERBB2
F12	EFEMP1	BTC	ERBB4
FBLN1	FCGR2B	C1QA	CD93
FBLN2	FCGR3A	C3	C3AR1
FBN1	FCGR3B	C4A	C3AR1
FBN2	FGFR1	C5	C5AR1
FGA	FGFR3	CALCA	CALCRL
FGF1	FGFR2	CALCA	RAMP1
FGF2	FGFR4	CALCA	RAMP2

FGF3	FLT1	CALR	LRP1
FGF4	FLT3	CCK	CCKAR
FGF5	FLT4	CCK	CCKBR
FGF6	FPR1	CCL1	CCR1
FGF7	DARC	CCL1	CCR8
FGF8	GALR1	CCL11	CCBP2
FGF9	GCGR	CCL11	CCR2
FGF10	GFRA1	CCL11	CCR3
FGG	GFRA2	CCL11	CCR5
FIGF	GFRA3	CCL11	CCRL1
FLT3LG	GHR	CCL11	CXCR3
FN1	GHRHR	CCL12	CCBP2
GAST	GHSR	CCL13	CCBP2
GAS6	GIPR	CCL13	CCR2
GC	GLP1R	CCL13	CCR3
GCG	GNRHR	CCL13	CCR5
MSTN	GP1BA	CCL13	CCRL1
GDF9	GP1BB	CCL13	CXCR3
GDNF	CCR10	CCL14	CCBP2
GH1	XCR1	CCL14	CCR1
GH2	NPBWR1	CCL14	CCR3
GHRH	NPBWR2	CCL14	CCR5
GIF	CXCR3	CCL15	CCR1
GIP	PRLHR	CCL15	CCR3
GNRH1	UTS2R	CCL16	CCR1
CXCL1	MCHR1	CCL16	CCR2
CXCL2	MLNR	CCL16	CCR5
CXCL3	GRPR	CCL16	CCR8
GRP	HCRTR1	CCL16	CXCR5
HCRT	HCRTR2	CCL17	CCR4
HGF	ICAM1	CCL17	CCR8
HGFAC	IFNAR1	CCL17	DARC
NRG1	IFNAR2	CCL19	CCBP2
HMGB1	IFNGR1	CCL19	CCR10
HP	IFNGR2	CCL19	CCR7
TNC	IGF1R	CCL19	CCRL1
IFNA1	IGF2R	CCL2	CCBP2
IFNA2	IL1R1	CCL2	CCR1
IFNA5	IL1RAP	CCL2	CCR10
IFNA8	IL2RA	CCL2	CCR2
IFNB1	IL2RB	CCL2	CCR3
IFNG	IL2RG	CCL2	CCR5
IFNW1	IL3RA	CCL2	CCRL1
IGF1	IL4R	CCL2	CCRL2
IGF2	IL5RA	CCL2	DARC
IGFBP3	IL6R	CCL20	CCR6
CYR61	IL6ST	CCL20	CXCR3

IGHG1	IL7R	CCL21	CCBP2
IHH	CXCR1	CCL21	CCR10
IL1A	CXCR2	CCL21	CCR7
IL1B	IL9R	CCL21	CCRL1
IL2	IL10RA	CCL21	CXCR3
IL3	IL10RB	CCL22	CCR4
IL4	IL11RA	CCL23	CCR1
IL5	IL12B	CCL24	CCR3
IL6	IL12RB1	CCL25	CCBP2
IL7	IL12RB2	CCL25	CCR10
IL8	IL13RA1	CCL25	CCR9
IL9	IL13RA2	CCL25	CCRL1
IL10	IL15RA	CCL26	CCR1
IL11	TNFRSF9	CCL26	CCR3
IL12A	INSR	CCL27	CCBP2
IL13	ITGA6	CCL27	CCR10
IL15	ITGA1	CCL28	CCBP2
IL17A	ITGA2	CCL28	CCR10
IL18	ITGA2B	CCL28	CCR3
INHA	ITGA3	CCL3	CCBP2
INHBA	ITGA4	CCL3	CCR1
INHBB	ITGA5	CCL3	CCR3
INHBC	ITGA9	CCL3	CCR4
CXCL10	ITGAD	CCL3	CCR5
INS	ITGAM	CCL3	CXCR5
INSL3	ITGAV	CCL3L1	CCR1
KISS1	ITGAX	CCL3L1	CCR3
KNG1	ITGB1	CCL3L1	CCR5
LAMA2	ITGB3	CCL4	CCBP2
LAMA3	ITGB4	CCL4	CCR1
LAMA4	ITGB5	CCL4	CCR3
LAMA5	ITGB6	CCL4	CCR5
LAMB1	ITGB7	CCL4	CCR8
LAMB2	ITGB8	CCL4	CXCR5
LAMB3	KDR	CCL5	CCBP2
LAMC1	KIT	CCL5	CCR1
LBP	LDLR	CCL5	CCR3
LCN1	LEPR	CCL5	CCR4
LCN2	LHCGR	CCL5	CCR5
LEP	LIFR	CCL5	CCRL1
LGALS1	LRP1	CCL5	CXCR3
LGALS3	LRP2	CCL5	CXCR5
LGALS3BP	LRP6	CCL5	DARC
LHB	LRP5	CCL7	CCBP2
LIF	LTBP1	CCL7	CCR1
LIPC	LTBR	CCL7	CCR10
LPA	MAS1	CCL7	CCR2

LPL	MC1R	CCL7	CCR3
LTA	MC2R	CCL7	CCR5
LTB	MC3R	CCL7	CCRL1
LTF	MC4R	CCL7	CXCR3
MATN1	MC5R	CCL7	DARC
MBL2	MET	CCL8	CCBP2
MDK	MPL	CCL8	CCR1
KITLG	MST1R	CCL8	CCR2
CXCL9	NEO1	CCL8	CCR3
MLN	NGFR	CCL8	CCR5
MMP1	NMBR	CCL8	CCRL1
MMP7	NOTCH1	CCL8	DARC
MMP9	NOTCH2	CD40LG	CD40
MMP13	NOTCH3	CD59	CD2
MST1	NPR1	CEACAM1	CLEC4M
NCAM1	NPR2	CGA	LHCGR
NDP	NPR3	CHAD	ITGA2
NGF	NPY1R	CHAD	ITGB1
NID1	NPY2R	CHGB	FGFR3
NMB	NPY5R	CLCA2	ITGB4
NPY	NTRK1	CLCF1	CNTFR
NOV	NTRK2	CLU	LEPR
NPPA	NTRK3	CLU	LRP2
NPPB	ROR1	CLU	LRP8
NPPC	DDR2	CNTF	CNTFR
NPTX2	NTSR1	CNTF	LIFR
NRTN	TNFRSF11B	COL11A1	DDR1
NTF3	OPRL1	COL11A2	DDR2
NTF4	OXTR	COL14A1	CD44
NTN3	PDGFRA	COL17A1	ITGA6
NTS	PDGFRB	COL17A1	ITGB4
ORM1	PLAUR	COL18A1	ITGA5
OSM	NPY4R	COL18A1	KDR
OXT	PRLR	COL1A1	CD36
PEBP1	PTCH1	COL1A1	CD44
SERPINE1	PTH1R	COL1A1	CD93
PDGFA	PTH2R	COL1A1	DDR2
PDGFB	PTPRC	COL1A1	ITGA2
PF4	RET	COL1A1	ITGA5
PGF	ROBO1	COL1A1	ITGB1
PLAT	ROBO2	COL1A2	CD36
PLAU	RYK	COL1A2	CD44
PLG	SORT1	COL1A2	CD93
PMCH	SCTR	COL1A2	ITGA2
PNOC	SPN	COL1A2	ITGA2B
POMC	SSTR1	COL1A2	ITGB3
PPBP	SSTR2	COL2A1	CD36

PPY	SSTR3	COL2A1	DDR1
SRGN	SSTR4	COL2A1	ITGA2B
PRL	SSTR5	COL3A1	DDR1
PSPN	TACR2	COL3A1	DDR2
PROC	TACR1	COL4A1	CD36
RELN	TACR3	COL4A1	CD93
PYY	TEK	COL4A2	ANTXR2
PTH	TFR2	COL4A2	CD36
PTHLH	TFRC	COL4A2	CD93
PTN	TGFBR1	COL4A3	ANTXR2
REN	TGFBR2	COL4A3	CD93
RLN1	TGFBR3	COL4A3	ITGAV
RLN2	THBD	COL4A3	ITGB3
S100A12	TIE1	COL4A4	ANTXR2
S100B	TNFRSF1A	COL4A4	CD93
SAA1	TNFRSF1B	COL4A5	CD93
SCT	TRHR	COL4A6	CD93
CCL1	TSHR	COL5A2	DDR1
CCL2	TNFRSF4	COL6A1	CD36
CCL3	TYRO3	COL6A3	ITGA1
CCL3L1	VIPR1	COL6A3	ITGA2
CCL4	VIPR2	COL6A3	ITGA5
CCL5	VLDLR	COL6A3	ITGB1
CCL7	ZP2	COL6A3	ITGB3
CCL8	LRP8	COL8A1	ITGA1
CCL11	IL1R2	COL8A1	ITGA2
CCL13	CXCR4	CORT	GHSR
CCL14	FZD5	CORT	MRGPRX2
CCL15	CUBN	CORT	SSTR1
CCL16	FZD1	CORT	SSTR2
CCL17	FZD4	CORT	SSTR3
CCL19	FZD6	CORT	SSTR4
CCL20	FZD8	CORT	SSTR5
CCL21	FZD9	CREG1	IGF2R
CCL22	LTBP4	CRELD2	CHRNA4
CCL23	ATRN	CRELD2	CHRNB2
CCL24	ITGA8	CRH	CRHR1
CCL25	UNC5C	CRH	CRHR2
CXCL6	PTCH2	CRLF1	CNTFR
CXCL11	TNFRSF25	CSF1	CSF1R
CXCL5	TNFRSF14	CSF2	CSF2RA
XCL1	TNFRSF6B	CSF2	CSF2RB
CX3CL1	TNFRSF18	CSF2	CSF3R
CXCL12	TNFRSF11A	CSF2	IL3RA
SECTM1	TNFRSF10D	CSF3	CSF3R
SEMA3F	TNFRSF10C	CTF1	IL6ST
SFTPD	TNFRSF10B	CTF1	LIFR

SHH	TNFRSF10A	CTGF	ERBB4
SLIT1	IL18RAP	CTGF	ITGA5
SLIT3	IL1RL2	CTGF	LRP1
SIGLEC1	IL18R1	CX3CL1	CX3CR1
SPP1	GALR2	CXCL1	CXCR1
SST	NRP2	CXCL1	CXCR2
XCL2	NRP1	CXCL1	DARC
TAC1	F2RL3	CXCL10	CCR3
TAC3	CCRL2	CXCL10	CXCR3
TF	IL1RL1	CXCL11	CCR3
TG	OSMR	CXCL11	CXCR3
TGFA	CD163	CXCL12	CCR10
TGFB1	GLP2R	CXCL12	CCR4
TGFB1I1	ZFYVE9	CXCL12	CXCR4
TGFB2	NRXN1	CXCL13	CCR10
TGFB3	IL27RA	CXCL13	CCRL1
THBS1	ATP6AP2	CXCL13	CXCR3
THPO	CALCRL	CXCL13	CXCR5
TIMP3	RAMP2	CXCL16	CXCR6
TNF	RAMP1	CXCL2	CXCR1
TRH	NMUR1	CXCL2	CXCR2
TSHB	CLEC4M	CXCL3	CXCR1
TTR	MERTK	CXCL3	CXCR2
TNFSF4	PROCR	CXCL5	CXCR1
UCN	CXCR6	CXCL5	CXCR2
SCGB1A1	CCR9	CXCL5	DARC
COL14A1	NPFFR2	CXCL6	CXCR1
VEGFA	EDAR	CXCL6	CXCR2
VEGFB	IL11RA1	CXCL9	CCR3
VEGFC	ZP3R	CXCL9	CXCR3
VIP	KLRK1	CYR61	ITGAD
VTN	CD93	CYR61	ITGAV
VWF	PLXND1	CYR61	ITGB5
WNT1	NPTXR	DCD	TNFRSF14
WNT2	TNFRSF13B	DHH	PTCH1
WNT3	NTSR2	DHH	PTCH2
WNT5A	IL17RA	DKK1	LRP5
WNT7A	IL21R	DKK1	LRP6
XDH	SLC22A17	DKK2	LRP6
ZP3	TNFRSF12A	DLK1	NOTCH2
SEMA3B	CCRL1	DLL1	NOTCH1
FGF23	LRP1B	DLL1	NOTCH2
GDF5	ADAM22	DLL1	NOTCH3
TNFSF11	IL20RA	DPP4	PTPRC
NPFF	IL20RB	DSCAM	DCC
TNFSF14	FGFRL1	EBI3	IL27RA
TNFSF13	ROBO4	EDA	EDA2R

TNFSF12	IL17RB	EDA	EDAR
TNFSF10	LMBR1L	EDIL3	ITGAV
TNFSF9	NMUR2	EDN1	EDNRA
DLK1	IL22RA1	EDN1	EDNRB
CREG1	RXFP1	EDN3	EDNRA
FGF18	EDA2R	EDN3	EDNRB
FGF17	GFRA4	EFNA1	EPHA1
WISP2	NPFFR1	EFNA1	EPHA2
APLN	TNFRSF23	EFNA1	EPHA3
TNFSF18	TNFRSF22	EFNA1	EPHA4
ARTN	ADIPOR2	EFNA1	EPHA5
ANGPTL1	TM2D1	EFNA1	EPHA6
BMP15	MCHR2	EFNA1	EPHA7
LGI1	KISS1R	EFNA1	EPHA8
CRLF1	IL17RC	EFNA1	EPHB1
SLIT2	UNC5A	EFNA4	EPHA2
ADIPOQ	TNFRSF13C	EFNA4	EPHA4
NTN1	IL22RA2	EFNA4	EPHA5
NRG2	MRGPRX2	EFNA4	EPHA7
CLCA2	ANTXR2	EFNA4	EPHA8
SEMA3E	RXFP2	EGF	EGFR
FGF19	ACVR1C	EGF	ERBB2
TNFSF15	GPR151	EGF	ERBB3
EDIL3	IL23R	EMILIN1	ITGA4
EBI3	AMOT	EMILIN1	ITGB1
GDF11	IFNLR1	EPO	EPOR
CCL26	TRHR2	EREG	EGFR
SEMA3A	UNC5B	EREG	ERBB4
SEMA3C	EPHA6	F11	GP1BA
CXCL13	RXFP4	F12	CD93
TNFSF13B	CCR2	F12	GP1BA
NRG3		F2	F2R
CCL27		F2	F2RL1
NMU		F2	F2RL2
UTS2		F2	F2RL3
IL24		F2	GP1BA
ZPBP		F2	ITGA2B
EMILIN1		F2	ITGA5
INSL6		F2	ITGB3
NXPH3		F2	THBD
NXPH2		F8	ASGR2
PDAP1		F8	LRP1
CCL12		FASLG	FAS
DKK1		FASLG	TNFRSF10B
CLCF1		FASLG	TNFRSF6B
DKK2		FBLN1	ITGB1
IL17B		FBLN2	ITGB3

ANGPTL3	FBN1	LTBP1
DLL1	FBN2	LTBP1
IL19	FGA	ITGA2B
NXPH1	FGA	ITGB3
IL20	FGF1	FGFR1
IL22	FGF1	FGFR2
DHH	FGF1	FGFR3
PRLH	FGF1	FGFR4
GAL	FGF1	NRP1
ANGPT4	FGF10	FGFR2
IL23A	FGF17	FGFR1
GHRL	FGF17	FGFR2
TLR9	FGF17	FGFR3
WNT4	FGF17	FGFR4
IL26	FGF18	FGFR1
PDGFC	FGF18	FGFR2
CCL28	FGF18	FGFR3
CXCL16	FGF18	FGFR4
IL21	FGF19	FGFR4
NTN4	FGF2	FGFR1
IL25	FGF2	FGFR2
CRELD2	FGF2	FGFR3
PDGFD	FGF2	FGFR4
ULBP2	FGF2	FGFRL1
COL18A1	FGF2	NRP1
JAM3	FGF23	FGFR2
IL1F10	FGF23	FGFR3
TSLP	FGF3	FGFR1
WNT3A	FGF3	FGFR2
UCN2	FGF3	FGFR3
IL33	FGF3	FGFR4
IL17F	FGF4	FGFR1
UCN3	FGF4	FGFR2
DCD	FGF4	FGFR3
RLN3	FGF4	FGFR4
NRG4	FGF4	NRP1
AGR3	FGF5	FGFR1
SEMA3D	FGF5	FGFR2
IL27	FGF5	FGFR3
NPNT	FGF5	FGFR4
NPB	FGF6	FGFR1
UTS2B	FGF6	FGFR2
IFNL2	FGF6	FGFR3
IFNL3	FGF6	FGFR4
IFNL1	FGF7	FGFR1
NPW	FGF7	FGFR2
LAMA1	FGF7	FGFR3

AGRN		FGF7	FGFR4
GDF6		FGF7	NRP1
SFTPA1		FGF8	FGFR1
SFTPA2		FGF8	FGFR2
MICA		FGF8	FGFR3
		FGF8	FGFR4
		FGF9	FGFR1
		FGF9	FGFR2
		FGF9	FGFR3
		FGF9	FGFR4
		FGG	ICAM1
		FGG	ITGAM
		FGG	ITGB3
		FIGF	FLT4
		FIGF	KDR
		FLT3LG	FLT3
		FN1	ITGA3
		FN1	ITGA4
		FN1	ITGA5
		FN1	ITGA8
		FN1	ITGAV
		FN1	ITGB1
		FN1	ITGB3
		FN1	ITGB6
		FN1	ITGB7
		FN1	LTBP1
		GAL	GALR1
		GAL	GALR2
		GAL	GPR151
		GAS6	AXL
		GAS6	MERTK
		GAS6	TYRO3
		GAST	CCKBR
		GC	CUBN
		GC	LRP2
		GCG	GCGR
		GCG	GIPR
		GCG	GLP1R
		GCG	GLP2R
		GDF11	ACVR1B
		GDF11	ACVR1C
		GDF11	ACVR2B
		GDF11	TGFBR1
		GDF5	ACVR1
		GDF5	ACVR2A
		GDF5	ACVR2B
		GDF5	BMPR1A

GDF5	BMPR1B
GDF5	BMPR2
GDF6	BMPR1A
GDF6	BMPR1B
GDF6	BMPR2
GDF9	ACVR2A
GDF9	BMPR1A
GDF9	BMPR1B
GDF9	BMPR2
GDNF	GFRA1
GDNF	GFRA2
GDNF	RET
GH1	GHR
GH1	PRLR
GH2	GHR
GHRH	GHRHR
GHRL	GHRHR
GHRL	GHSR
GHRL	MLNR
GIF	CUBN
GIP	GIPR
GNRH1	GNRHR
GRP	GRPR
HBEGF	CD44
HBEGF	EGFR
HBEGF	ERBB4
HCRT	HCRTR1
HCRT	HCRTR2
HGF	MET
HGFAC	MET
HMGB1	AGER
HP	CD163
HP	CD22
IFNA1	CR2
IFNA1	IFNAR2
IFNA2	IFNAR1
IFNA2	IFNAR2
IFNA5	IFNAR2
IFNA8	IFNAR1
IFNA8	IFNAR2
IFNB1	IFNAR1
IFNB1	IFNAR2
IFNG	IFNGR1
IFNG	IFNGR2
IFNL1	IL10RB
IFNL1	IFNLR1
IFNL2	IL10RB

IFNL2	IFNLR1
IFNL3	IL10RB
IFNL3	IFNLR1
IFNW1	IFNAR1
IGF1	IGF1R
IGF2	IGF1R
IGF2	IGF2R
IGF2	INSR
IGFBP3	IGF1R
IGFBP3	LTBP1
IGHG1	FCGR2B
IGHG1	FCGR3A
IGHG1	FCGR3B
IHH	PTCH1
IHH	PTCH2
IL10	IL10RA
IL10	IL10RB
IL11	IL11RA
IL11	IL11RA1
IL12A	IL12B
IL12A	IL12RB1
IL12A	IL12RB2
IL13	IL13RA1
IL13	IL13RA2
IL13	IL4R
IL15	IL15RA
IL15	IL2RB
IL15	IL2RG
IL17A	IL17RA
IL17A	IL17RC
IL17B	IL17RB
IL17F	IL17RA
IL17F	IL17RC
IL18	IL18R1
IL18	IL18RAP
IL18	IL1RL2
IL19	IL20RA
IL19	IL20RB
IL1A	IL1R1
IL1A	IL1R2
IL1A	IL1RAP
IL1B	IL1R1
IL1B	IL1R2
IL1B	IL1RAP
IL1F10	IL1R1
IL2	IL21R
IL2	IL2RA

IL2	IL2RB
IL2	IL2RG
IL20	IL20RA
IL20	IL20RB
IL21	IL21R
IL21	IL2RG
IL22	IL10RB
IL22	IL22RA1
IL22	IL22RA2
IL23A	IL12B
IL23A	IL12RB1
IL23A	IL23R
IL24	IL20RA
IL24	IL20RB
IL25	IL17RA
IL25	IL17RB
IL26	IL20RA
IL27	IL27RA
IL3	CSF2RB
IL3	IL3RA
IL33	IL1RL1
IL4	IL13RA1
IL4	IL13RA2
IL4	IL2RG
IL4	IL4R
IL5	CSF2RB
IL5	IL5RA
IL6	IL6R
IL6	IL6ST
IL7	IL2RG
IL7	IL7R
IL8	CXCR1
IL8	CXCR2
IL8	DARC
IL9	IL2RG
IL9	IL9R
INHA	ACVR2A
INHA	TGFBR3
INHBA	ACVR1
INHBA	ACVR1B
INHBA	ACVR2A
INHBA	ACVR2B
INHBA	ENG
INHBA	TGFBR3
INHBB	ACVR1
INHBB	ACVR1B
INHBB	ACVR1C

INHBB	ACVR2A
INHBB	ACVR2B
INHBC	ACVR1
INHBC	ACVR1B
INHBC	ACVR2A
INHBC	ACVR2B
INS	IGF1R
INS	INSR
INS	LRP2
INSL3	RXFP2
INSL6	ALK
JAG1	NOTCH1
JAG1	NOTCH2
JAG1	NOTCH3
JAM3	F2RL2
JAM3	ITGAM
JAM3	ITGAX
KISS1	KISS1R
KITLG	KIT
KNG1	CD93
KNG1	GP1BA
KNG1	PLAUR
LAMA1	DAG1
LAMA1	ITGA1
LAMA1	ITGA2
LAMA1	ITGB1
LAMA2	DAG1
LAMA2	ITGA6
LAMA3	ITGA3
LAMA4	ITGA3
LAMA4	ITGA5
LAMA4	ITGB1
LAMA4	ITGB3
LAMA5	DAG1
LAMA5	ITGA6
LAMA5	ITGB4
LAMB1	ITGA6
LAMB1	ITGB4
LAMB2	ITGA6
LAMB2	ITGB4
LAMB3	ITGA6
LAMC1	ITGA6
LAMC1	ITGB4
LBP	CD14
LCN1	LMBR1L
LCN2	LRP2
LCN2	SLC22A17

LEP	LEPR
LGALS1	CD2
LGALS1	PTPRC
LGALS3	PTPRC
LGALS3BP	CD14
LGALS3BP	ITGB1
LGI1	ADAM22
LHB	LHCGR
LIF	IL6ST
LIF	LIFR
LIPC	LRP1
LPA	LRP2
LPL	LRP1
LPL	LRP2
LPL	VLDLR
LTA	LTBR
LTA	TNFRSF1A
LTA	TNFRSF1B
LTB	TNFRSF1A
LTF	CD14
LTF	LRP1
MATN1	ITGA1
MBL2	CD93
MDK	ALK
MDK	LRP1
MDK	LRP2
MICA	KLRK1
MLN	MLNR
MMP1	CD44
MMP1	F2R
MMP13	LRP1
MMP7	CD44
MMP9	CD44
MST1	MST1R
MSTN	ACVR2B
NCAM1	FGFR1
NCAM1	GFRA1
NDP	FZD4
NGF	NGFR
NGF	NTRK1
NGF	ROR1
NGF	SORT1
NID1	ITGAV
NID1	ITGB1
NID1	ITGB3
NMB	NMBR
NMU	NMUR1

NMU	NMUR2
NOV	ITGAV
NOV	ITGB1
NOV	ITGB3
NOV	NOTCH1
NPB	NPBWR1
NPB	NPBWR2
NPFF	NPFFR1
NPFF	NPFFR2
NPNT	ITGA8
NPPA	NPR1
NPPA	NPR2
NPPA	NPR3
NPPB	NPR1
NPPB	NPR2
NPPB	NPR3
NPPC	NPR2
NPPC	NPR3
NPTX2	NPTXR
NPW	NPBWR1
NPW	NPBWR2
NPY	MC4R
NPY	NPFFR2
NPY	NPY1R
NPY	NPY2R
NPY	NPY5R
NPY	NPY4R
NRG1	EGFR
NRG1	ERBB2
NRG1	ERBB3
NRG1	ERBB4
NRG2	ERBB3
NRG2	ERBB4
NRG3	ERBB4
NRG4	ERBB4
NRTN	GFRA1
NRTN	GFRA2
NTF3	NTRK1
NTF3	NTRK2
NTF3	NTRK3
NTF4	NGFR
NTF4	NTRK2
NTN1	DCC
NTN1	NEO1
NTN1	UNC5A
NTN1	UNC5B
NTN1	UNC5C

NTN3	NEO1
NTN4	NEO1
NTS	NTSR1
NTS	NTSR2
NXPH1	NRXN1
NXPH2	NRXN1
NXPH3	NRXN1
ORM1	CCR5
OSM	IL6ST
OSM	LIFR
OSM	OSMR
OXT	OXTR
PDAP1	PDGFRB
PDGFA	PDGFRA
PDGFB	LRP1
PDGFB	PDGFRA
PDGFB	PDGFRB
PDGFC	PDGFRA
PDGFD	PDGFRB
PEBP1	ADRBK1
PF4	CXCR3
PF4	DARC
PF4	LDLR
PGF	FLT1
PGF	NRP1
PGF	NRP2
PLAT	LRP1
PLAT	LRP1B
PLAU	LRP2
PLAU	PLAUR
PLG	AMOT
PMCH	MCHR1
PMCH	MCHR2
PNOC	OPRL1
POMC	MC1R
POMC	MC2R
POMC	MC4R
POMC	MC5R
PPBP	CXCR1
PPBP	CXCR2
PPY	NPY4R
PRL	PRLR
PRLH	PRLHR
PROC	PROCR
PSPN	GFRA4
PTH	PTH1R
PTH	PTH2R

PTHLH	PTH1R
PTN	ALK
PYY	NPY1R
PYY	NPY2R
PYY	NPY5R
PYY	NPY4R
RELN	LRP8
RELN	VLDLR
REN	ATP6AP2
RLN1	RXFP1
RLN2	RXFP1
RLN2	RXFP2
RLN3	RXFP1
RLN3	RXFP4
S100A12	AGER
S100B	AGER
SAA1	FPR1
SCGB1A1	CUBN
SCGB1A1	LRP2
SCT	SCTR
SECTM1	CD7
SEMA3A	NRP1
SEMA3B	NRP1
SEMA3B	NRP2
SEMA3C	NRP1
SEMA3C	NRP2
SEMA3D	NRP1
SEMA3E	PLXND1
SEMA3F	NRP1
SEMA3F	NRP2
SERPINE1	LRP1B
SERPINE1	LRP2
SERPINE1	PLAUR
SFTPA1	DMBT1
SFTPA2	CD93
SFTPD	DMBT1
SHH	PTCH1
SHH	PTCH2
SIGLEC1	SPN
SLIT1	ROBO1
SLIT2	ROBO1
SLIT2	ROBO2
SLIT2	ROBO4
SLIT3	ROBO2
SPP1	CD44
SRGN	CD44
SST	SSTR1

SST	SSTR2
SST	SSTR3
SST	SSTR4
SST	SSTR5
TAC1	TACR1
TAC1	TACR2
TAC1	TACR3
TAC3	TACR1
TAC3	TACR2
TAC3	TACR3
TF	TFR2
TF	TFRC
TG	ASGR1
TG	LRP2
TGFA	EGFR
TGFA	ERBB2
TGFA	ERBB4
TGFB1	ACVRL1
TGFB1	ENG
TGFB1	LTBP4
TGFB1	TGFBR1
TGFB1	TGFBR2
TGFB1	TGFBR3
TGFB1I1	TGFBR2
TGFB2	ENG
TGFB2	TGFBR1
TGFB2	TGFBR2
TGFB2	TGFBR3
TGFB3	ACVRL1
TGFB3	ENG
TGFB3	TGFBR1
TGFB3	TGFBR2
TGFB3	TGFBR3
THBS1	CD36
THBS1	CD47
THPO	MPL
TIMP3	AGTR2
TIMP3	EFEMP1
TLR9	AGER
TNC	ITGA5
TNC	ITGA8
TNC	ITGA9
TNC	ITGB1
TNC	ITGB6
TNF	LTBR
TNF	TNFRSF1A
TNF	TNFRSF1B

TNFSF10	TNFRSF10A
TNFSF10	TNFRSF10B
TNFSF10	TNFRSF10C
TNFSF10	TNFRSF10D
TNFSF10	TNFRSF11B
TNFSF10	TNFRSF22
TNFSF10	TNFRSF23
TNFSF11	TNFRSF11A
TNFSF11	TNFRSF11B
TNFSF12	TNFRSF12A
TNFSF12	TNFRSF25
TNFSF13	TNFRSF11B
TNFSF13	TNFRSF13B
TNFSF13	TNFRSF14
TNFSF13	TNFRSF17
TNFSF13	TNFRSF1A
TNFSF13B	TNFRSF13B
TNFSF13B	TNFRSF13C
TNFSF13B	TNFRSF17
TNFSF14	LTBR
TNFSF14	TNFRSF14
TNFSF14	TNFRSF6B
TNFSF15	TNFRSF25
TNFSF15	TNFRSF6B
TNFSF18	TNFRSF18
TNFSF4	TNFRSF4
TNFSF8	TNFRSF8
TNFSF9	TNFRSF9
TRH	TRHR
TRH	TRHR2
TSHB	TSHR
TSLP	IL7R
TTR	AGER
TTR	DDR1
TTR	LRP2
UCN	CRHR1
UCN	CRHR2
UCN2	CRHR2
UCN3	CRHR2
ULBP2	KLRK1
UTS2	UTS2R
UTS2B	UTS2R
VEGFA	FLT1
VEGFA	KDR
VEGFA	NRP1
VEGFA	NRP2
VEGFB	FLT1

VEGFB	NRP1
VEGFC	FLT4
VEGFC	KDR
VIP	VIPR1
VIP	VIPR2
VTN	ITGA8
VTN	ITGAD
VTN	ITGAV
VTN	ITGB3
VTN	ITGB5
VTN	ITGB8
VWF	GP1BA
VWF	GP1BB
VWF	ITGA2B
WISP2	IGF1R
WISP2	IGF2R
WNT1	FZD8
WNT1	FZD9
WNT1	LRP5
WNT1	LRP6
WNT1	RYK
WNT2	FZD1
WNT2	FZD9
WNT3	FZD1
WNT3A	FZD1
WNT3A	LRP1
WNT3A	LRP6
WNT3A	RYK
WNT4	FZD6
WNT5A	FZD1
WNT5A	FZD5
WNT7A	FZD5
WNT7A	FZD9
XCL1	XCR1
XCL2	XCR1
XDH	BTN1A1
ZP3	ZP3R
ZPBP	ZP2

Supplementary Table S4
HFHSD

SECRETORY TISSUE	RECEPTOR TISSUE	Number of Interactions							
		Day 1	Day 6	Day 14	Day 35	Day 56	Day 77	Day 98	Day 119
Adipose_BA	Adipose_BA	53	0	0	0	0	2	28	9
Adipose_BA	Adipose_EA	17	1	0	0	0	0	9	0
Adipose_BA	Adipose_SA	28	1	0	0	0	1	0	1
Adipose_BA	Hippo	15	0	0	0	0	0	0	0
Adipose_BA	Liver	24	0	3	1	0	0	4	0
Adipose_BA	Skeletal	75	0	1	0	0	0	0	0
Adipose_BA	Spleen	77	0	5	0	0	1	3	2
Adipose_BA	svc_BA	41	0	0	2	0	0	17	3
Adipose_BA	svc_EA	5	3	0	0	0	0	1	3
Adipose_BA	svc_SA	46	3	0	0	0	0	0	0
Adipose_EA	Adipose_BA	23	0	0	0	0	2	13	6
Adipose_EA	Adipose_EA	6	2	0	0	2	0	3	3
Adipose_EA	Adipose_SA	12	4	0	0	0	2	0	0
Adipose_EA	Hippo	6	0	0	0	0	0	0	0
Adipose_EA	Liver	13	1	0	0	0	0	0	0
Adipose_EA	Skeletal	32	0	0	0	0	0	0	0
Adipose_EA	Spleen	29	0	0	2	0	0	2	1
Adipose_EA	svc_BA	16	3	0	1	0	0	5	7
Adipose_EA	svc_EA	5	6	0	0	0	0	0	0
Adipose_EA	svc_SA	14	8	0	0	0	0	0	0
Adipose_SA	Adipose_BA	29	0	0	0	0	3	4	2
Adipose_SA	Adipose_EA	10	5	0	0	0	0	0	1
Adipose_SA	Adipose_SA	12	3	0	0	0	2	0	0
Adipose_SA	Hippo	6	0	0	0	0	1	0	0
Adipose_SA	Liver	12	1	0	0	0	0	1	0
Adipose_SA	Skeletal	38	0	0	0	1	0	0	0
Adipose_SA	Spleen	37	0	0	1	0	0	0	0
Adipose_SA	svc_BA	21	4	1	1	0	0	1	1
Adipose_SA	svc_EA	4	7	1	0	0	0	0	0
Adipose_SA	svc_SA	20	11	0	0	0	0	0	0
Hippo	Adipose_BA	13	0	0	0	0	0	1	0
Hippo	Adipose_EA	5	0	0	0	0	0	0	0
Hippo	Adipose_SA	11	0	0	0	0	0	0	0
Hippo	Hippo	4	0	0	0	0	1	0	0
Hippo	Liver	14	0	1	0	2	0	0	0
Hippo	Skeletal	24	0	1	0	0	1	0	0
Hippo	Spleen	21	0	0	0	0	0	0	0
Hippo	svc_BA	8	0	4	0	0	0	0	0
Hippo	svc_EA	1	1	0	0	0	0	0	0
Hippo	svc_SA	8	1	0	0	1	0	0	0
Liver	Adipose_BA	16	0	1	0	0	0	3	1
Liver	Adipose_EA	4	0	0	0	0	0	2	0
Liver	Adipose_SA	12	0	0	1	0	0	0	0
Liver	Hippo	6	0	1	0	0	0	0	0
Liver	Liver	9	1	1	3	0	0	2	0
Liver	Skeletal	29	0	0	0	0	0	0	0
Liver	Spleen	28	0	1	5	0	0	0	0
Liver	svc_BA	12	0	0	6	0	0	3	0
Liver	svc_EA	1	1	0	0	0	0	0	0
Liver	svc_SA	18	0	0	0	1	0	0	0
Skeletal	Adipose_BA	65	0	1	0	0	0	2	0
Skeletal	Adipose_EA	15	0	0	0	0	1	2	0
Skeletal	Adipose_SA	31	0	1	0	0	2	0	0
Skeletal	Hippo	13	0	0	0	0	1	0	0
Skeletal	Liver	28	0	2	0	0	0	0	0
Skeletal	Skeletal	93	0	0	0	0	0	0	0
Skeletal	Spleen	82	0	1	0	0	0	0	0
Skeletal	svc_BA	41	1	0	0	0	0	1	0
Skeletal	svc_EA	10	0	0	0	0	1	1	0
Skeletal	svc_SA	42	2	0	0	0	0	0	0
Spleen	Adipose_BA	66	0	1	0	0	0	0	5
Spleen	Adipose_EA	17	1	0	0	0	0	0	0
Spleen	Adipose_SA	31	1	1	0	0	0	0	0
Spleen	Hippo	12	0	2	0	0	0	0	0

Spleen	Liver	33	0	6	3	0	0	0	0
Spleen	Skeletal	99	0	0	0	0	0	0	0
Spleen	Spleen	93	0	3	3	0	0	0	1
Spleen	svc_BA	44	1	2	5	0	0	0	2
Spleen	svc_EA	9	1	1	0	0	0	0	0
Spleen	svc_SA	43	4	0	0	1	0	0	0
svc_BA	Adipose_BA	15	0	0	0	0	0	14	8
svc_BA	Adipose_EA	5	9	0	0	0	0	3	0
svc_BA	Adipose_SA	8	4	0	3	0	0	0	0
svc_BA	Hippo	4	1	0	0	0	0	2	0
svc_BA	Liver	7	1	0	3	0	0	4	0
svc_BA	Skeletal	20	1	0	0	0	0	0	0
svc_BA	Spleen	25	0	0	5	0	0	2	4
svc_BA	svc_BA	11	8	0	6	0	0	14	5
svc_BA	svc_EA	0	5	0	0	0	0	4	2
svc_BA	svc_SA	15	15	0	0	0	0	0	1
svc_EA	Adipose_BA	7	1	1	0	0	0	4	0
svc_EA	Adipose_EA	1	11	0	0	2	0	2	0
svc_EA	Adipose_SA	1	4	0	0	0	0	0	0
svc_EA	Hippo	2	0	0	0	0	0	1	0
svc_EA	Liver	1	1	1	0	0	0	0	0
svc_EA	Skeletal	5	1	0	0	0	0	0	0
svc_EA	Spleen	6	0	1	0	0	0	1	0
svc_EA	svc_BA	1	9	1	0	0	0	6	0
svc_EA	svc_EA	0	9	1	0	1	0	1	0
svc_EA	svc_SA	3	19	0	0	2	0	0	0
svc_SA	Adipose_BA	29	1	0	0	0	0	0	0
svc_SA	Adipose_EA	7	13	0	0	1	0	1	0
svc_SA	Adipose_SA	15	7	0	0	0	0	0	0
svc_SA	Hippo	5	0	0	0	0	0	1	0
svc_SA	Liver	13	3	0	0	0	0	0	0
svc_SA	Skeletal	41	1	0	0	1	0	0	0
svc_SA	Spleen	45	0	0	0	1	0	1	0
svc_SA	svc_BA	18	9	0	0	1	0	1	0
svc_SA	svc_EA	2	13	0	0	1	0	0	0
svc_SA	svc_SA	23	19	0	0	3	0	0	0

HFHSD+Kal1

SECRETORY TISSUE	RECEPTOR TISSUE	Number of Interactions							
		Day 1	Day 6	Day 14	Day 35	Day 56	Day 77	Day 98	Day 119
Adipose_BA	Adipose_BA	*NA*	*NA*	*NA*	0	0	1	21	3
Adipose_BA	Adipose_EA	*NA*	*NA*	*NA*	0	0	1	0	0
Adipose_BA	Adipose_SA	*NA*	*NA*	*NA*	0	0	2	1	0
Adipose_BA	Hippo	*NA*	*NA*	*NA*	0	0	0	0	0
Adipose_BA	Liver	*NA*	*NA*	*NA*	1	0	0	7	0
Adipose_BA	Skeletal	*NA*	*NA*	*NA*	0	0	0	0	0
Adipose_BA	Spleen	*NA*	*NA*	*NA*	6	0	0	10	0
Adipose_BA	svc_BA	*NA*	*NA*	*NA*	2	0	0	2	2
Adipose_BA	svc_EA	*NA*	*NA*	*NA*	0	0	1	13	1
Adipose_BA	svc_SA	*NA*	*NA*	*NA*	1	0	1	2	1
Adipose_EA	Adipose_BA	*NA*	*NA*	*NA*	1	0	1	0	0
Adipose_EA	Adipose_EA	*NA*	*NA*	*NA*	0	0	1	0	0
Adipose_EA	Adipose_SA	*NA*	*NA*	*NA*	0	0	1	0	4
Adipose_EA	Hippo	*NA*	*NA*	*NA*	0	0	0	0	0
Adipose_EA	Liver	*NA*	*NA*	*NA*	1	0	0	0	0
Adipose_EA	Skeletal	*NA*	*NA*	*NA*	0	0	1	0	0
Adipose_EA	Spleen	*NA*	*NA*	*NA*	2	0	0	0	1
Adipose_EA	svc_BA	*NA*	*NA*	*NA*	1	0	0	0	1
Adipose_EA	svc_EA	*NA*	*NA*	*NA*	1	0	0	0	0
Adipose_EA	svc_SA	*NA*	*NA*	*NA*	0	0	0	0	1
Adipose_SA	Adipose_BA	*NA*	*NA*	*NA*	0	0	2	0	0
Adipose_SA	Adipose_EA	*NA*	*NA*	*NA*	0	0	1	0	0
Adipose_SA	Adipose_SA	*NA*	*NA*	*NA*	0	0	1	0	0
Adipose_SA	Hippo	*NA*	*NA*	*NA*	0	0	0	0	0
Adipose_SA	Liver	*NA*	*NA*	*NA*	0	0	0	0	0
Adipose_SA	Skeletal	*NA*	*NA*	*NA*	0	0	0	0	0
Adipose_SA	Spleen	*NA*	*NA*	*NA*	2	0	0	1	0
Adipose_SA	svc_BA	*NA*	*NA*	*NA*	1	0	0	1	0
Adipose_SA	svc_EA	*NA*	*NA*	*NA*	0	0	0	1	0

Adipose_SA	svc_SA	*NA*	*NA*	*NA*	0	0	0	0	0
Hippo	Adipose_BA	*NA*	*NA*	*NA*	0	0	0	1	0
Hippo	Adipose_EA	*NA*	*NA*	*NA*	0	0	1	0	0
Hippo	Adipose_SA	*NA*	*NA*	*NA*	0	0	1	0	0
Hippo	Hippo	*NA*	*NA*	*NA*	0	0	0	0	0
Hippo	Liver	*NA*	*NA*	*NA*	0	2	0	0	0
Hippo	Skeletal	*NA*	*NA*	*NA*	0	0	1	0	0
Hippo	Spleen	*NA*	*NA*	*NA*	0	2	0	0	0
Hippo	svc_BA	*NA*	*NA*	*NA*	0	0	1	0	0
Hippo	svc_EA	*NA*	*NA*	*NA*	0	0	2	0	0
Hippo	svc_SA	*NA*	*NA*	*NA*	0	1	0	0	0
Liver	Adipose_BA	*NA*	*NA*	*NA*	1	0	0	3	0
Liver	Adipose_EA	*NA*	*NA*	*NA*	0	0	0	0	0
Liver	Adipose_SA	*NA*	*NA*	*NA*	1	0	0	0	0
Liver	Hippo	*NA*	*NA*	*NA*	0	0	0	0	0
Liver	Liver	*NA*	*NA*	*NA*	1	0	0	2	0
Liver	Skeletal	*NA*	*NA*	*NA*	0	0	0	0	0
Liver	Spleen	*NA*	*NA*	*NA*	8	0	0	0	0
Liver	svc_BA	*NA*	*NA*	*NA*	1	1	1	1	0
Liver	svc_EA	*NA*	*NA*	*NA*	0	0	0	1	0
Liver	svc_SA	*NA*	*NA*	*NA*	1	0	0	0	0
Skeletal	Adipose_BA	*NA*	*NA*	*NA*	0	0	0	5	0
Skeletal	Adipose_EA	*NA*	*NA*	*NA*	0	0	1	0	0
Skeletal	Adipose_SA	*NA*	*NA*	*NA*	0	0	3	0	0
Skeletal	Hippo	*NA*	*NA*	*NA*	0	0	0	0	0
Skeletal	Liver	*NA*	*NA*	*NA*	0	0	0	1	0
Skeletal	Skeletal	*NA*	*NA*	*NA*	0	0	7	0	0
Skeletal	Spleen	*NA*	*NA*	*NA*	0	0	1	1	0
Skeletal	svc_BA	*NA*	*NA*	*NA*	0	0	1	0	0
Skeletal	svc_EA	*NA*	*NA*	*NA*	0	0	1	4	0
Skeletal	svc_SA	*NA*	*NA*	*NA*	0	0	1	0	0
Spleen	Adipose_BA	*NA*	*NA*	*NA*	3	0	0	8	0
Spleen	Adipose_EA	*NA*	*NA*	*NA*	0	0	1	0	0
Spleen	Adipose_SA	*NA*	*NA*	*NA*	2	0	1	0	0
Spleen	Hippo	*NA*	*NA*	*NA*	2	0	0	0	0
Spleen	Liver	*NA*	*NA*	*NA*	3	1	0	0	0
Spleen	Skeletal	*NA*	*NA*	*NA*	0	0	0	0	0
Spleen	Spleen	*NA*	*NA*	*NA*	50	3	1	3	0
Spleen	svc_BA	*NA*	*NA*	*NA*	18	3	1	0	0
Spleen	svc_EA	*NA*	*NA*	*NA*	1	2	2	1	0
Spleen	svc_SA	*NA*	*NA*	*NA*	1	1	0	0	0
svc_BA	Adipose_BA	*NA*	*NA*	*NA*	1	0	0	3	1
svc_BA	Adipose_EA	*NA*	*NA*	*NA*	0	0	1	0	0
svc_BA	Adipose_SA	*NA*	*NA*	*NA*	0	0	0	0	0
svc_BA	Hippo	*NA*	*NA*	*NA*	1	0	0	0	0
svc_BA	Liver	*NA*	*NA*	*NA*	1	0	0	1	0
svc_BA	Skeletal	*NA*	*NA*	*NA*	0	0	0	0	0
svc_BA	Spleen	*NA*	*NA*	*NA*	16	5	0	1	0
svc_BA	svc_BA	*NA*	*NA*	*NA*	4	3	0	1	1
svc_BA	svc_EA	*NA*	*NA*	*NA*	0	2	1	1	0
svc_BA	svc_SA	*NA*	*NA*	*NA*	0	0	0	0	0
svc_EA	Adipose_BA	*NA*	*NA*	*NA*	0	0	0	11	0
svc_EA	Adipose_EA	*NA*	*NA*	*NA*	0	0	0	0	1
svc_EA	Adipose_SA	*NA*	*NA*	*NA*	0	0	0	0	0
svc_EA	Hippo	*NA*	*NA*	*NA*	0	0	0	0	0
svc_EA	Liver	*NA*	*NA*	*NA*	0	0	0	5	0
svc_EA	Skeletal	*NA*	*NA*	*NA*	0	1	0	0	0
svc_EA	Spleen	*NA*	*NA*	*NA*	0	0	0	6	0
svc_EA	svc_BA	*NA*	*NA*	*NA*	0	3	0	0	0
svc_EA	svc_EA	*NA*	*NA*	*NA*	0	1	0	10	0
svc_EA	svc_SA	*NA*	*NA*	*NA*	0	1	0	0	0
svc_SA	Adipose_BA	*NA*	*NA*	*NA*	0	0	0	2	0
svc_SA	Adipose_EA	*NA*	*NA*	*NA*	0	0	0	0	0
svc_SA	Adipose_SA	*NA*	*NA*	*NA*	0	0	0	0	0
svc_SA	Hippo	*NA*	*NA*	*NA*	0	0	0	0	0
svc_SA	Liver	*NA*	*NA*	*NA*	0	0	0	0	0
svc_SA	Skeletal	*NA*	*NA*	*NA*	0	0	0	0	0
svc_SA	Spleen	*NA*	*NA*	*NA*	0	0	0	2	0
svc_SA	svc_BA	*NA*	*NA*	*NA*	0	2	0	1	0

svc_SA	svc_EA	*NA*	*NA*	*NA*	0	0	0	1	0
svc_SA	svc_SA	*NA*	*NA*	*NA*	0	0	0	0	0

Supplementary Table S5

Secretory	Receptor	Day 1	Day 6	Day 14	Week 3	Week 6	Week 9	Week 12	Week 15	No. of days on which interaction occurs
APOB	LRP2	42	1	0	1	0	1	4	1	6
MDK	LRP2	28	1	0	1	1	0	6	1	6
APLN	APLNR	16	1	1	3	1	0	2	0	6
FN1	ITGA3	15	3	1	1	0	0	2	1	6
OSM	LIFR	15	3	1	0	0	1	1	1	6
FGF23	FGFR2	18	3	2	0	0	0	2	1	5
FGF2	FGFR2	15	1	0	1	0	0	2	1	5
FN1	ITGB6	15	9	1	0	1	0	0	2	5
PLAU	LRP2	14	0	0	1	1	0	4	1	5
FN1	ITGA4	9	12	0	1	0	0	2	1	5
NPPB	NPR3	45	0	1	0	0	0	1	1	4
TNC	ITGA8	42	3	1	0	0	0	3	0	4
NPNT	ITGA8	36	3	0	0	0	1	3	0	4
NRG1	ERBB3	35	0	0	0	1	6	3	0	4
WNT5A	FZD5	35	8	0	1	0	0	2	0	4
TNC	ITGB6	35	9	1	0	0	0	0	2	4
NPPA	NPR3	35	2	0	1	0	0	1	0	4
IFNA1	CR2	30	9	0	0	0	0	2	3	4
NRG1	ERBB4	30	0	0	0	0	3	6	4	4
SIGLEC1	SPN	30	1	0	2	0	0	1	0	4
NCAM1	GFRA1	25	1	0	1	0	0	9	0	4
THPO	MPL	24	4	0	0	0	1	2	0	4
FN1	LTBP1	21	6	1	0	0	0	2	0	4
BTC	ERBB4	18	1	1	0	0	0	4	0	4
EREG	ERBB4	18	0	1	0	0	0	8	4	4
FN1	ITGA8	18	3	1	0	0	0	6	0	4
IL1A	IL1R2	15	0	1	0	0	0	1	3	4
TGFA	ERBB4	12	4	0	2	0	0	2	0	4
FN1	ITGB3	12	6	1	1	0	0	0	0	4
OSM	OSMR	10	1	1	0	0	0	1	0	4
FBN1	LTBP1	7	2	2	0	0	0	1	0	4
VEGFA	FLT1	6	1	1	2	0	0	0	0	4
INHBB	ACVR1C	5	1	1	0	0	0	0	1	4
MMP9	CD44	5	0	1	1	1	0	0	0	4
SLIT2	ROBO1	4	4	1	0	0	0	1	0	4
FN1	ITGAV	3	6	0	0	0	0	2	1	4
COL4A3	ITGAV	2	2	0	0	0	0	1	1	4
EGFR	MET	1	1	1	1	0	0	0	0	4
HGF	MET	1	1	0	1	0	0	1	0	4
LAMA3	ITGA3	30	1	2	0	0	0	0	0	3
IL24	IL20RB	28	0	0	0	0	0	2	1	3
NPY	PPYR1	24	4	4	0	0	0	0	0	3
IGF2	INSR	20	1	0	0	0	0	0	1	3
COL6A3	ITGA2	20	0	0	0	0	0	3	1	3
COL6A3	ITGB3	20	2	1	0	0	0	0	0	3
PDGFB	PDGFRA	20	6	0	0	0	0	1	0	3
FGF1	FGFR2	18	2	0	0	0	0	0	1	3
PLG	AMOT	16	1	0	0	0	0	3	0	3
IGF2	IGF1R	16	1	0	0	0	0	0	1	3
NPY	NPFFR2	16	8	0	0	0	0	0	2	3
NTS	NTSR2	16	2	0	0	0	0	1	0	3
CXCL12	CCR4	15	0	2	0	0	0	2	0	3
COL1A1	CD44	15	0	0	0	1	0	2	0	3
LIFR	CNTFR	15	9	0	0	0	0	0	2	3
PLAT	LRP1B	14	0	0	2	0	0	1	0	3
CALCA	CALCRL	12	1	0	0	0	0	2	0	3
CXCL12	CXCR4	12	0	2	0	0	0	1	0	3
FGF23	FGFR3	12	3	0	0	0	0	6	0	3
COL1A1	ITGA2	12	0	0	0	0	0	3	1	3
JAG1	NOTCH2	12	0	1	0	0	0	1	0	3
TNFSF11	TNFRSF11A	12	3	0	0	0	0	2	0	3
FGF2	FGFR3	10	1	0	0	0	0	6	0	3
IL22	IL22RA2	10	0	0	0	0	1	1	0	3
COL6A3	ITGA1	10	1	0	0	0	0	2	0	3
INHBC	ACVR1B	9	0	0	1	0	0	3	0	3
IL1A	IL1RAP	9	0	0	0	0	0	3	3	3
COL2A1	DDR1	8	3	0	2	0	0	0	0	3
COL4A3	ITGB3	8	2	1	0	0	0	0	0	3
FGFR2	EPHA4	6	1	0	0	0	0	2	0	3
LGI1	ADAM22	4	1	0	0	0	0	2	0	3
KNG1	PLAUR	4	1	0	0	0	0	3	0	3
BTC	EGFR	3	1	1	0	0	0	0	0	3
DPP4	PTPRC	3	4	0	0	0	0	0	6	3
TGFA	EGFR	2	4	0	1	0	0	0	0	3
CALCA	RAMP1	2	2	0	0	0	0	1	0	3
DKK2	LRP6	1	0	0	1	2	0	0	0	3
C3	C3AR1	0	1	0	1	1	0	0	0	3
NPPB	NPR2	54	0	2	0	0	0	0	0	2
NPPA	NPR2	42	0	0	2	0	0	0	0	2
PNOC	OPRL1	42	0	0	0	0	0	6	0	2
TG	LRP2	35	2	0	0	0	0	0	0	2
GHRL	GHSR	30	0	0	0	0	0	1	0	2
TNC	ITGB1	28	0	0	0	0	0	1	0	2
PYY	PPYR1	24	2	0	0	0	0	0	0	2
EDN3	EDNRB	20	0	0	0	0	0	0	1	2
COL6A3	ITGB1	20	0	0	0	0	0	1	0	2
NXPH1	NRXN1	18	0	0	0	0	0	3	0	2
INHBB	ACVR1B	15	1	0	0	0	0	0	0	2
EDN3	EDNRA	15	1	0	0	0	0	0	0	2
IL7	IL7R	15	0	0	0	0	0	3	0	2
SLIT2	ROBO2	14	0	0	0	0	0	2	0	2
UCN	CRHR1	12	4	0	0	0	0	0	0	2
CCL7	DARC	12	2	0	0	0	0	0	0	2
COL1A1	DDR2	12	0	0	0	0	0	0	1	2
FGF1	FGFR3	12	2	0	0	0	0	0	0	2
IL2	IL2RA	12	0	2	0	0	0	0	0	2
COL1A1	ITGB1	12	0	0	0	0	0	1	0	2
FN1	ITGB1	12	0	0	0	0	0	2	0	2
CCL7	CCR2	9	4	0	0	0	0	0	0	2
BTC	ERBB2	9	0	1	0	0	0	0	0	2
BDNF	NTRK2	9	0	0	0	1	0	0	0	2
GDF5	BMPR1A	8	6	0	0	0	0	0	0	2

CSF1	CSF1R	8	0	0	0	2	0	0	0	2
COL11A2	DDR2	8	1	0	0	0	0	0	0	2
COL8A1	ITGA1	8	1	0	0	0	0	0	0	2
NTN1	NEO1	8	2	0	0	0	0	0	0	2
INHA	ACVR2A	6	3	0	0	0	0	0	0	2
BMP5	BMPR1A	6	4	0	0	0	0	0	0	2
BMP6	BMPR1A	6	2	0	0	0	0	0	0	2
COL1A1	CD93	6	0	0	0	0	0	0	1	2
VEGFB	FLT1	6	0	0	1	0	0	0	0	2
INHBB	ACVR2A	5	1	0	0	0	0	0	0	2
COL11A1	DDR1	5	2	0	0	0	0	0	0	2
GDF5	ACVR2A	4	3	0	0	0	0	0	0	2
COL4A3	CD93	4	0	0	0	0	0	0	1	2
COL3A1	DDR2	4	0	0	0	0	0	0	2	2
PLAU	PLAUR	4	0	0	0	0	0	6	0	2
CALCA	RAMP2	4	1	0	0	0	0	0	0	2
BMP6	ACVR2A	3	1	0	0	0	0	0	0	2
EREG	EGFR	3	0	1	0	0	0	0	0	2
EGFR	ERBB2	3	0	1	0	0	0	0	0	2
EDIL3	ITGAV	3	0	0	0	0	0	1	0	2
LIF	LIFR	3	0	0	0	0	0	0	2	2
LGALS3	PTPRC	3	2	0	0	0	0	0	0	2
BDNF	SORT1	3	0	0	1	0	0	0	0	2
BMP3	ACVR2A	1	1	0	0	0	0	0	0	2
SPP1	CD44	0	0	0	0	1	1	0	0	2
IL33	IL1RL1	0	0	0	0	1	1	0	0	2
TNC	ITGA9	0	0	1	0	0	0	1	0	2
FGG	ITGAM	0	4	1	0	0	0	0	0	2
FGG	ITGB3	0	4	1	0	0	0	0	0	2
LCN2	LRP2	0	0	0	1	0	1	0	0	2
SERPINE1	LRP2	0	0	0	2	1	0	0	0	2
TTR	LRP2	0	0	0	1	0	1	0	0	2
COL14A1	CD44	25	0	0	0	0	0	0	0	1
FGF1	FGFR1	24	0	0	0	0	0	0	0	1
CCL7	CCR3	21	0	0	0	0	0	0	0	1
CRLF1	CNTFR	20	0	0	0	0	0	0	0	1
FGF2	FGFR1	20	0	0	0	0	0	0	0	1
NCAM1	FGFR1	20	0	0	0	0	0	0	0	1
COL8A1	ITGA2	16	0	0	0	0	0	0	0	1
NRG1	ERBB2	15	0	0	0	0	0	0	0	1
IFNG	IFNGR2	12	0	0	0	0	0	0	0	1
LAMA4	ITGA3	10	0	0	0	0	0	0	0	1
BDNF	NGFR	9	0	0	0	0	0	0	0	1
COL4A6	CD93	8	0	0	0	0	0	0	0	1
FGFR1	EPHA4	8	0	0	0	0	0	0	0	1
LAMA4	ITGB1	8	0	0	0	0	0	0	0	1
LAMA4	ITGB3	8	0	0	0	0	0	0	0	1
PDGFA	PDGFRA	8	0	0	0	0	0	0	0	1
CCL24	CCR3	7	0	0	0	0	0	0	0	1
AGR3	DAG1	6	0	0	0	0	0	0	0	1
TGFA	ERBB2	6	0	0	0	0	0	0	0	1
IFNA1	IFNAR2	6	0	0	0	0	0	0	0	1
COL17A1	ITGB4	6	0	0	0	0	0	0	0	1
FGF1	NRP1	6	0	0	0	0	0	0	0	1
SEMA3F	NRP2	6	0	0	0	0	0	0	0	1
VEGFA	NRP2	6	0	0	0	0	0	0	0	1
NXPH3	NRXN1	6	0	0	0	0	0	0	0	1
TGFB1I1	TGFBR2	6	0	0	0	0	0	0	0	1
NRG1	EGFR	5	0	0	0	0	0	0	0	1
DKK1	LRP5	5	0	0	0	0	0	0	0	1
DKK1	LRP6	5	0	0	0	0	0	0	0	1
FGF2	NRP1	5	0	0	0	0	0	0	0	1
GDF6	BMPR1A	4	0	0	0	0	0	0	0	1
COL4A1	CD93	4	0	0	0	0	0	0	0	1
COL4A2	CD93	4	0	0	0	0	0	0	0	1
KNG1	CD93	4	0	0	0	0	0	0	0	1
FBLN1	ITGB1	4	0	0	0	0	0	0	0	1
SEMA3E	PLXND1	4	0	0	0	0	0	0	0	1
INHBC	ACVR2A	3	0	0	0	0	0	0	0	1
SEMA3D	NRP1	3	0	0	0	0	0	0	0	1
COL4A4	CD93	2	0	0	0	0	0	0	0	1
F12	CD93	2	0	0	0	0	0	0	0	1
SEMA3A	NRP1	2	0	0	0	0	0	0	0	1
SEMA3F	NRP1	2	0	0	0	0	0	0	0	1
VEGFA	NRP1	2	0	0	0	0	0	0	0	1
VEGFB	NRP1	2	0	0	0	0	0	0	0	1
COL3A1	DDR1	1	0	0	0	0	0	0	0	1
BMP7	ACVR2A	0	1	0	0	0	0	0	0	1
ADCYAP1	ADCYAP1R1	0	0	0	1	0	0	0	0	1
TTR	AGER	0	0	0	0	0	1	0	0	1
BMP7	BMPR1A	0	2	0	0	0	0	0	0	1
C4A	C3AR1	0	0	0	1	0	0	0	0	1
CCL7	CCBP2	0	0	0	0	1	0	0	0	1
CCL19	CCRL1	0	0	0	0	0	0	0	1	1
HP	CD163	0	0	0	0	1	0	0	0	1
HP	CD22	0	0	0	0	1	0	0	0	1
CNTF	CNTFR	0	0	0	2	0	0	0	0	1
TTR	DDR1	0	0	0	1	0	0	0	0	1
EDN1	EDNRB	0	0	0	0	0	0	0	1	1
BMP7	ENG	0	1	0	0	0	0	0	0	1
FGF8	FGFR2	0	0	0	0	0	0	0	1	1
FGF2	FGFR4	0	0	0	1	0	0	0	0	1
IGF1	IGF1R	0	1	0	0	0	0	0	0	1
IL22	IL22RA1	0	0	0	0	0	0	0	2	1
IL23A	IL23R	0	0	0	0	0	0	0	1	1
IL7	IL2RG	0	0	0	0	0	0	0	2	1
CYR61	ITGAV	0	0	0	0	0	0	1	0	1
NOV	ITGAV	0	0	0	0	0	2	0	0	1
FN1	ITGB7	0	0	0	1	0	0	0	0	1
SERPINE1	LRP1B	0	0	0	4	0	0	0	0	1
GC	LRP2	0	0	0	0	1	0	0	0	1
TGFB1	LTBP4	0	0	0	1	0	0	0	0	1
NMU	NMUR2	0	0	0	0	0	0	0	1	1
NPY	NPY5R	0	0	0	0	0	0	0	1	1
PDGFC	PDGFRA	0	3	0	0	0	0	0	0	1
PRL	PRLR	0	0	0	0	0	0	0	1	1

SLIT2	ROBO4	0	0	0	1	0	0	0	0	1
RELN	VLDLR	0	0	0	0	1	0	0	0	1

Supplementary Table S6

HFC								
Interaction		Days						No. of days the interaction occurs
Sec	Rec	35	56	77	98	119		
APOB	LRP2	1	0	1	4	1		4
**MDK**	**LRP2**	**1**	**1**	**0**	**6**	**1**		**4**
PLAU	LRP2	1	1	0	4	1		4
APLN	APLNR	3	1	0	2	0		3
FN1	ITGA3	1	0	0	2	1		3
OSM	LIFR	0	0	1	1	1		3
**FGF2**	**FGFR2**	**1**	**0**	**0**	**2**	**1**		**3**
FN1	ITGA4	1	0	0	2	1		3
**NRG1**	**ERBB3**	**0**	**1**	**6**	**3**	**0**		**3**
NRG1	ERBB4	0	0	3	6	4		3

HF75								
Interaction		Days						No. of days the interaction occurs
Sec	Rec	35	56	77	98	119		
**NRG1**	**ERBB3**	**2**	**2**	**2**	**6**	**2**		**5**
**FGF2**	**FGFR2**	**2**	**1**	**2**	**2**	**0**		**4**
BTC	ERBB4	2	0	6	4	0		3
THPO	MPL	2	0	1	0	1		3
NRG1	ERBB4	2	0	3	6	0		3
CRLF1	CNTFR	0	0	2	2	1		3
FGF2	FGFR3	4	0	1	2	0		3
LIFR	CNTFR	4	0	1	4	0		3
**MDK**	**LRP2**	**2**	**0**	**2**	**2**	**0**		**3**
NPNT	ITGA8	2	0	0	4	1		3

Supplementary Table S7

			P-value (two tailed : p1 ≠ p2) [α = 0.05]						
			**14**	**35**	**56**	**77**	**98**	**119**	**140**
**Metabolism Rec (2050)**	Up	HFHSD<br>HFHSD+KAL1	0.3989423	0.0149709	0.3499546	0.3221541	0.0933515	0.0480199	0.0574156
	Down	HFHSD<br>HFHSD+KAL1	0.3989423	0.3864746	0.3562809	0.0053641	0.3240196	0.3513682	0.0799158
**Metabolism Sec (2840)**	Up	HFHSD<br>HFHSD+KAL1	0.3989423	0.0056644	0.3175396	0.3989423	0.3593948	0.0450829	0.1878482
	Down	HFHSD<br>HFHSD+KAL1	0.3989423	0.2803433	0.3969748	0.0104114	0.036441	0.015181	0.3989423
**Inflammatory Rec (620)**	Up	HFHSD<br>HFHSD+KAL1	0.3989423	0.2762484	0.3664414	0.3927805	0.0469191	0.0235771	0.1267583
	Down	HFHSD<br>HFHSD+KAL1	0.3989423	0.3927805	0.3403343	0.2403944	0.3837333	0.3908818	0.3260956
**Inflammatory Sec (1080)**	Up	HFHSD<br>HFHSD+KAL1	0.3989423	0.131395	0.2079956	0.3585399	0.2287863	0.372863	0.3989423
	Down	HFHSD<br>HFHSD+KAL1	0.3989423	0.340759	0.3132605	0.3547135	0.036146	0.0917717	0.3517371

Supplementary Table S8

Correspoding to figure 5A P-value (two tailed : p1 ≠ p2) [α = 0.05]

	Rec		Sec	
	**Up**	**Down**	**Up**	**Down**
**Adipose_BA**	0.374442299	0.186886971	0.383967715	0.259302369
**Adipose_EA**	0.391003429	0.300136722	0.227174788	0.366146099
**Adipose_SA**	0.380908139	0.34370742	0.289691553	0.289691553
**Hippo**	NA	0.319780937	0.291713824	0.253577601
**Liver**	0.123347781	0.381907532	0.273217258	0.06351906
**Skeletal**	0.026168431	0.287689105	0.02830705	0.269758787
**Spleen**	0.343239672	0.39308468	0.087404077	0.304939264
**SCV_BA**	0.05980294	0.180342014	0.381882319	0.397321883
**SVC_EA**	0.39760608	0.39760608	0.136758267	0.265782619
**SVC_SA**	0.396919125	0.236159746	0.394276049	0.392972773

# Corresponding to figure 5B P-value (two tailed : p1 ≠ p2) [α = 0.05]

	Rec		Sec	
	**Sec Up & Rec Down**	**Sec Down & Rec Up**	**Sec Up & Rec Down**	**Sec Down & Rec Up**
**Adipose_BA**	0.371025666	0.376060243	0.38254877	0.38254877
**Adipose_EA**	#VALUE!	0.379057088	0.382323943	0.386319963
**Adipose_SA**	0.395918906	0.370884267	0.384326676	0.398138291
**Hippo**	NA	0.38276998	0.398782478	0.384687703
**Liver**	0.376120665	0.369281403	0.397421333	0.380818456
**Skeletal**	0.398229531	0.392507296	0.370887838	0.368663538
**Spleen**	0.368621897	0.368621897	0.395228996	0.385097499
**SCV_BA**	0.368722508	0.38797117	0.369108244	0.369297645
**SVC_EA**	0.39894228	0.39894228	0.39894228	0.39894228
**SVC_SA**	0.39894228	0.39894228	0.39894228	0.39894228
